# A Novel Gene Family Controls Species-Specific Morphological Traits in *Hydra*

**DOI:** 10.1371/journal.pbio.0060278

**Published:** 2008-11-18

**Authors:** Konstantin Khalturin, Friederike Anton-Erxleben, Sylvia Sassmann, Jörg Wittlieb, Georg Hemmrich, Thomas C. G Bosch

**Affiliations:** Zoological Institute, Christian-Albrechts-University, Am Botanishen Garten 1-9, 24118 Kiel, Germany; University of California, Berkeley, United States of America

## Abstract

Understanding the molecular events that underlie the evolution of morphological diversity is a major challenge in biology. Here, to identify genes whose expression correlates with species-specific morphologies, we compared transcriptomes of two closely related *Hydra* species. We find that species-specific differences in tentacle formation correlate with expression of a taxonomically restricted gene encoding a small secreted protein. We show that gain of function induces changes in morphology that mirror the phenotypic differences observed between species. These results suggest that “novel” genes may be involved in the generation of species-specific morphological traits.

## Author Summary

Closely related animal species share most of their genes, and only minor morphological differences allow us to tell them apart. The genetic basis for these differences may involve minor changes in the spatial and temporal activity of transcription factors—“regulator” genes—which are surprisingly conserved throughout the animal kingdom. However, every group of animals also has a small proportion of genes that are extremely variable among closely related species or even unique. Such genes are referred to as “novel,” “orphan,” or “taxonomically restricted.” Their functions and origins are often obscure. We have found that a family of novel genes is responsible for morphological differences between two closely related species of fresh water polyps called *Hydra*. A secreted protein encoded by a novel gene regulates the way in which tentacles develop. Our data indicate that novel genes may play a role in the creation of novel morphological features, thus representing one way how evolution works at the genus level. Appearance of novel genes may reflect evolutionary processes that allow animals to adapt in the best way to changing environmental conditions and new habitats.

## Abstract 3

"Novel" genes are involved in the evolution of morphological diversity in the basal metazoan*Hydra*.

## Introduction

Understanding the molecular events that underlie the evolution of morphological diversity is a major challenge in biology. Comparative studies have demonstrated that morphological evolution occurs through alterations in development [1–5]. Recent advances in genome sequencing have shown that all eumetazoan animals, from cnidarians to humans, share a handful of highly conserved signal transduction pathways that, together with several hundred conserved transcription factors, make up a molecular toolkit common for all living beings [6,7]. It has been proposed that morphological traits are altered in evolution when these conserved components obtain a novel spatiotemporal mode of expression [8]. For example, pigmentation patterns in insects are thought to evolve by modifications in the *cis*-regulatory elements of pigmentation genes [9–11]. In finches, a relationship has been revealed between species-specific beak morphology and the spatiotemporal expression of calmodulin and bone morphogenetic protein 4 (BMP4) [12,13]. Supporting that observation, heterochronic manipulations of BMP4 expression during chick development can reproduce the different patterns observed among Darwin's finches [14]. Rewiring of regulatory networks is the equally well source for morphological changes on a large scale as well as for inter-specific or even intra-specific variations [15].

There is, however, one much less appreciated source for the creation of morphological novelties. All genome and expressed sequence tag (EST) projects to date in every taxonomic group studied so far have uncovered a substantial fraction of genes that are without known homologs [16–18]. These “orphans” or “taxonomically restricted genes” (TRGs) are defined as being exclusively restricted to a particular taxonomic group [19]. For example, analysis of the phylum *Nematoda* has identified more than 20% of genes that were nematode-unique TRGs [20]. The draft genome of Ciona intestinalis revealed [21] that nearly one-fifth of the genes were orphans. A comparison between the genome sequences of Schizosaccharomyces pombe and Saccharomyces cerevisiae showed [22] about 14% of the predicted proteins to be unique to *Sc. pombe* and 19% unique to *Sa. cerevisiae*. In *Drosophila*, TRGs include indispensable regulators of development such as *bicoid* [23] and *spätzle* [24]. Recent comparative data on the genomes of 12 *Drosophila* species revealed that about 2.5% of genes are not present outside of the genus *Drosophila* and, therefore, have most likely arisen de novo [25]. An even larger proportion of lineage-specific genes have been detected in the genome of *Tribolium* [26]. In bacteria, the cumulative number of orphans identified does not appear to be leveling off, although hundreds of complete genome sequences have been already analyzed [18,19]. Models for evolution of TRGs have been proposed [27] and the significance of their evolutionary contribution to ecological adaptation has been postulated [19]. Despite this, TRGs are poorly studied and little understood, in large part because the lack of homology confounds attempts to determine the putative function of the protein.

Thus, although progress has been made towards understanding the molecular mechanisms controlling the evolution of morphology, many questions remain to be addressed: What genes are differentially expressed in two closely related species and how many? Do the differences occur predominantly in structural or regulatory genes? Do novel genes play a role in generation of morphological novelties? And, ultimately, what is the genetic basis of species-specific morphologies?

**Figure 1:**
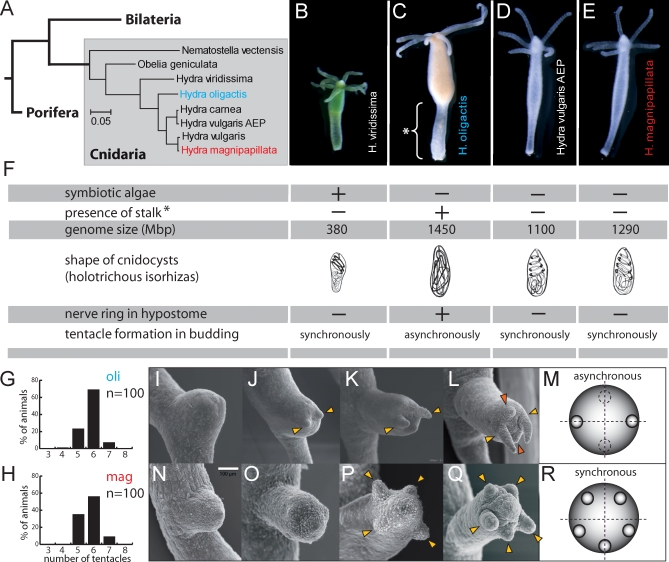
Variation in Morphological Traits among Different *Hydra* Species (A) Phylogenetic relationships at the base of animal evolution. Cnidaria are often regarded as a sister group to all Bilateria. Within the genus *Hydra*, H. viridissima has a basal position. H. oligactis is sister to H. magnipapillata and H. vulgaris [34]. (B–E) Wild-type phenotypes of four species frequently used in laboratories. (F) Summary of morphological and molecular differences between the four species. (G) Number of tentacles per polyp in H. oligactis (oli; *n* = 100). (H) Number of tentacles per polyp in H. magnipapillata (mag; *n* = 100). (I–L) Representative scanning electron micrographs of bud evagination in H. oligactis. (M) Scheme of tentacle formation during budding in H. oligactis. (N–Q) Scanning electron micrographs of bud evagination in wild-type H. magnipapillata line 105. (R) Scheme of tentacle formation during budding in H. magnipapillata.

Here, we address these fundamental questions in a study designed to identify the transcriptional signatures of two closely related, yet morphologically distinct species of the basal metazoan *Hydra*. Freshwater polyps of the genus *Hydra* belong to one of the earliest branches in the animal tree of life, the Cnidaria (Figure 1A), and they represent the simplest animals at the tissue grade of organization. The body plan consists of only two cell layers and a limited number of cell types derived from three distinct stem cell lineages [28]. A head and tentacles are on one end and a foot on the opposite end of a single body axis. Tentacles are used for prey capture and are armed with several types of nematocytes. Proliferation occurs mostly asexually by budding, a process during which a secondary polyp forms in the lower gastric region of the parental animal. Morphological simplicity in cnidarians is accompanied by unexpected molecular complexity. With the continuously growing EST and genomic databases from cnidarians, evidence is accumulating for extensive conservation in gene content, structure and organization between cnidarians and vertebrates [6,7,29–31]. At the same time, systematic approaches to isolate morphogenetically active molecules have identified numerous peptides in *Hydra* that are encoded by novel genes [32].

Here we show that in *Hydra*, a family of novel genes, defined as genus-level TRGs, plays a significant role in controlling phenotypic features that are referred to as species-specific traits. Our data show that morphological diversity at the genus level can be generated through changes in the spatial and temporal deployment of genes that are not highly conserved across long evolutionary distances. We also propose that losses and duplications of those novel genes among closely related species may be one of the driving forces leading to morphological diversification in the genus *Hydra*.

## Results

### Species-Specific Traits in the Genus *Hydra*

*Hydra* species differ in morphology, development, physiology, and ecology [33,34]. As shown in Figure 1B–1F, *Hydra* species differ in the presence or absence of symbionts, in carotenoid metabolism, and in the presence or absence of a conspicuous stalk structure (labeled with an asterisk in Figure 1C). *Hydra* species vary in genome sizes, ranging from 380 to 1,450 Mbp. *Hydra* species also differ in the shapes and sizes of their nematocysts. For example, as schematically shown in Figure 1F, in Hydra oligactis in contrast to other *Hydra* species the inverted tube of holotrichous isorhiza is coiled irregularly. Other species-specific traits include a ring of neurons running circumferentially around the hypostome in H. oligactis but not in other *Hydra* species [35]. *Hydra* species can also be identified by the order in which tentacles arise on young buds. Whereas the mean number of tentacles per polyp does not differ in H. oligactis and H. magnipapillata (Figure 1G and 1H), in H. oligactis two lateral tentacles arise early and are conspicuously longer during bud development (Figure 1I–1M) in contrast to H. magnipapillata and H. vulgaris, where tentacles arise synchronously and are all of the same length (Figure 1N–1R).

### Identification of TRGs in H. oligactis and H. magnipapillata

**Figure 2:**
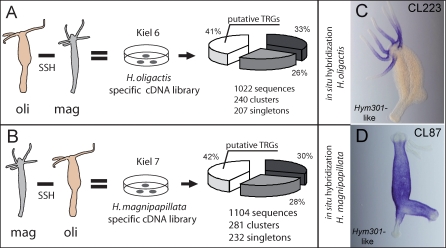
Identification of Species-Specific Genes in *Hydra* by SSH (A) Using H. magnipapillata cDNAs as driver and H. oligactis cDNAs as tester, we generated a library enriched for the transcripts of H. oligactis-specific genes. 1,022 cDNA clones were sequenced and clustered: 33% of the clusters and singletons had strong similarity to known proteins (dark grey segment, *E* value ≤ 1e–20), 26% had weak similarity (light grey segment, 1e–20 < *E* value < 1e–5); 41% had no similarity (white segment, *E* value ≥ 1e–5), representing, therefore, putative TRGs. (B) Using H. oligactis cDNAs as driver and H. magnipapillata cDNAs as tester we generated a library enriched for the transcripts of H. magnipapillata-specific genes. 1,104 cDNA clones were sequenced and clustered: 30% of the clusters and singletons had strong similarity to known proteins (dark grey segment, *E* value ≤ 1e–20), 28% had weak similarity (light grey segment, 1e–20 < *E* value < 1e–5) and 42% had no similarity (white segment, *E* value ≥ 1e–5), representing, therefore, putative TRGs. (C, D) Whole-mount in situ hybridization shows differences in the expression patterns of two homologous *Hydra*-specific genes in H. oligactis and H. magnipapillata. *CL223* and *CL87* have weak similarity to Hym301 protein [37] and exhibit complementary expression patterns that correlate with differences in the mode of tentacle formation between H. oligactis and H. magnipapillata.

To gain insight into the molecular mechanisms controlling phenotypic differences in the genus *Hydra*, we compared the transcriptomes of H. oligactis and H. magnipapillata by suppression subtractive hybridization (SSH) of complimentary DNAs (Figure 2A and 2B). The goal of SSH was to identify quickly evolving genes that are present (and transcriptionally active) in one species and absent (or transcriptionally silent) in the other, as well as to uncover homologous genes that are expressed in both species but have considerably diverged sequences. As shown schematically in Figure 2A and 2B, two subtractive hybridizations were performed using cDNA pools from H. oligactis and H. magnipapillata polyps, resulting in two cDNA libraries enriched for species-specific transcripts. As expected, a considerable proportion of clusters and singletons in each of the library had no homologs in the NCBI protein database (Figure 2A and 2B), representing, therefore, genes putatively restricted to the genus *Hydra* (for clustering and sequence analysis see Tables S1–S3 and Figure S1). The expression of these genes was analyzed by in situ hybridization. Genes expressed in taxonomically informative structures (tentacles, nematocytes, and stalk; see Figure S1) were selected for further analysis. Here, we focus on genes represented by clusters *CL223* and *CL87*. Both genes show no similarity to any gene outside the genus *Hydra*. Both genes, however, are weakly similar (see Tables S1 and S2) to the Hym301 protein, which was identified earlier in a systematic approach to isolate morphogenetically active peptides in H. magnipapillata [36]. Interestingly, as shown in Figure 2C and 2D, in the two species analyzed these *Hym301*-like genes have complementary expression patterns: whereas in H. oligactis expression is restricted to tentacles (Figure 2C), in *H. magnipapillata Hym301*-like transcripts are absent in tentacle tissue but abundant throughout the body column (Figure 2D). Because tentacle development is one of the major morphogenetic processes in *Hydra*, differs between H. oligactis and H. magnipapillata (Figure 1M and 1R), and is under strong selective constraints as tentacles are the only structures by which polyps can take up food, we focused on the identified *Hym301*-like genes to investigate their function.

### Characterization of the TRG Family Hym301

**Figure 3:**
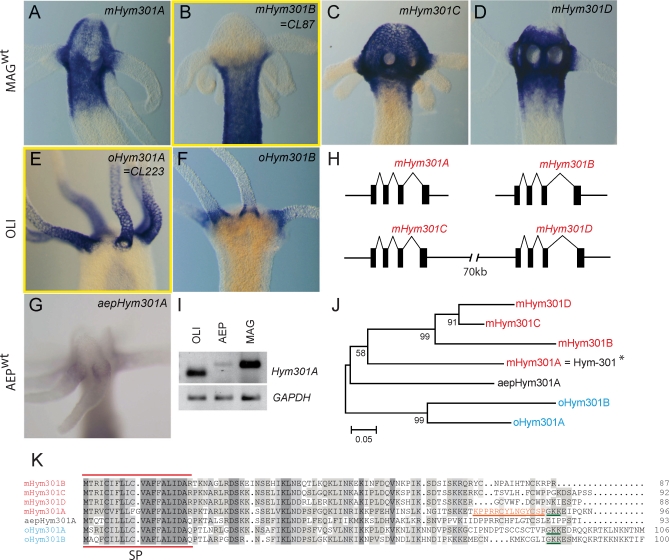
Analysis of the Taxonomically Restricted Gene Family Hym301 in Different *Hydra* Species (A–D) Expression of the four *mHym301* genes in H. magnipapillata. *mHym301B* and *oHym301A* identified in the SSH screening are boxed in yellow. (E–F) Expression of the two *oHym301* genes in H. oligactis. (G) Expression of the single *aepHym301* gene in H. vulgaris AEP. (H) Exon–intron structure of the four *mHym301* genes in H. magnipapillata. Note that *mHym301C* and *mHym301D* are clustered in the genome. (I) RT-PCR confirms the expression data in (G) and shows a much lower level of *Hym301* transcripts in H. vulgaris AEP than in H. oligactis and H. magnipapillata. (J) Phylogenetic tree showing the divergence of the Hym301 proteins in the three *Hydra* species*. H. magnipapillata*, red; H. vulgaris AEP, black; H. oligactis, blue. mHym301A is identical to the previously described Hym301 protein [37] and is labeled with an asterisk. (K) Alignment of the Hym301 amino acid sequences from three species of *Hydra*. The position of the Hym301 peptide described in [37] is underlined and in orange letters. The putative amidation signal (GKK) present in mHym301A, oHym301A, and oHym301B is underlined in green. SP, signal peptide.

Detailed analysis of the *Hym301*-like genes *CL223* (in H. oligactis) and *CL87* (in H. magnipapillata) revealed that they belong to a gene family that exhibits variation in gene number and expression patterns among *Hydra* species (Figure 3). In H. magnipapillata we discovered four *CL223*-related genes (see Materials and Methods for detail). Because one of them has been identified earlier [36] and was described as a gene *Hym301* in H. magnipapillata [37], we named the four genes *mHym301A* to *mHym301D*. In *H. magnipapillata*, the genes *mHym301A* (Figure 3A), *mHym301C* (Figure 3C), and *mHym301D* (Figure 3D) are expressed in the head, whereas *mHym301B* (Figure 3B) is expressed along the body column. None of the four H. magnipapillata genes is expressed in the tentacles. In contrast, H. oligactis has only two *Hym301*-related genes, *oHym301A* and *oHym301B*, with both genes expressed in tentacles (Figure 3E and 3F). In H. vulgaris AEP, only one gene is present (*aepHym301A*) and expressed in the basis of tentacles (Figure 3G). Expression of *aepHym301A* is considerably weaker than expression of the *Hym301*-like genes in the other two species (Figure 3I).

A phylogenetic tree based on the amino acid sequence alignment (Figure 3J) places the members of Hym301 gene family in three distinct groups, which fits with the phylogenetic tree of the genus *Hydra* [34] (see Figure 1A). Predicted proteins from H. oligactis and H. magnipapillata form two distinct clades, whereas the single protein from H. vulgaris AEP forms a separate clade (Figure 3J). The phylogeny clearly indicates that gene duplications occurred separately in the H. oligactis and H. magnipapillata lineages. In H. magnipapillata the available draft genome revealed that all four genes share common genomic organization, with four exons (Figure 3H). Interestingly, *mHym301C* and *mHym301D* are clustered in the genome within 70 kb (Figure 3H), suggesting a recent duplication event. Amino acid alignment (Figure 3K) indicates that all members of the gene family encode short proteins (87–106 amino acids) with a putative signal peptide sequence at the N terminus followed by a relatively conserved region. The C-terminal part of the protein is highly diverged, not only among species, but also among the different Hym301 proteins in H. oligactis and H. magnipapillata. In *mHym301A* the C terminus harbors the peptide (marked in orange in Figure 3K) reported previously [37] to affect tentacle formation in H. magnipapillata. According to the sequence information available, the genes of the Hym301 family have no homologs in eukaryotic organisms outside the genus *Hydra*. They are also not present in the genome of *Nematostella*, which, as a representative of the class Anthozoa, occupies a more basal position in the phylogenetic tree than *Hydra*. Therefore, we consider the members of the Hym301 family as TRGs, which have most likely originated within the class Hydrozoa and expanded in the genus *Hydra*.

### Generation of Transgenic *Hydra* Overexpressing *mHym301A*

**Figure 4:**
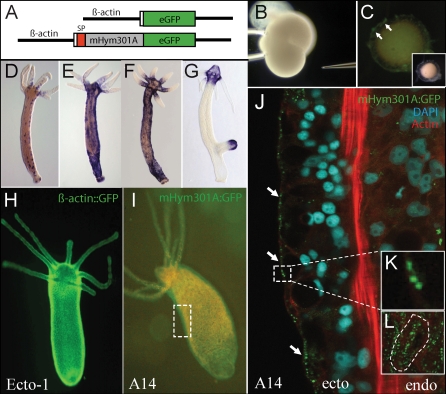
Transgenic H. vulgaris AEP Overexpressing mHym301A (A) Expression constructs for generation of transgenic *Hydra*. Top: control construct with eGFP driven by 1,386 bp actin 5′ flanking region. Below: construct with mHym301A fused to eGFP driven by the actin 5′ flanking region. SP, signal peptide. (B) *Hydra* embryo during the first cleavage stage used for microinjection of expression construct. (C) mHym310A:eGFP expression in *Hydra* embryos at cuticle stage (fluorescence microscopy). Arrows indicate accumulation of mHym310A:eGFP protein at the outer membrane, indicating secretion of the fusion protein. (D–F) RNA in situ hybridization reveals that *mHym301A* expression is activated in ectodermal epithelial cells all over the body column and includes tentacle epithelial cells. Founder polyps (D) are mosaics and were used to generate a mass culture of polyps such as that shown in (F) expressing *mHym301A* in most of the epithelial cells. (G) *mHym301A* expression in wild-type H. magnipapillata polyps is restricted to the head region and tip of the emerging bud. (H) Control transgenic polyp with eGFP expression under control of the β-actin promoter. (I) Transgenic polyp in which most of the ectodermal epithelial cells are expressing mHym301A:eGFP. Note that the eGFP signal is localized in ectoderm. (J) Confocal analysis of a representative transgenic polyp showing mHym301A:eGFP protein localization in secretory vesicles of ectodermal (ecto) epithelial cells (arrows). Green, eGFP protein; blue, DAPI stained nucleus; red, actin filaments. Endo, endodermal epithelium. (K) mHym301A:eGFP protein localizes to secretory vesicles in ectodermal epithelial cells. (L) Top view of ectodermal epithelial cell containing numerous eGFP-positive vesicles (see also Figure S2).

To explore the biological significance of the Hym301 gene family in vivo, we produced transgenic polyps that overexpress *mHym301A* in all ectodermal epithelial cells. As shown in Figure 4, we introduced the *H. magnipapillata mHym301A* gene into polyps of the closely related species H. vulgaris AEP, in which the expression level of the endogenous gene (*aepHym301A*) is low (see Figure 3G and 3I). *Hydra* embryos were microinjected (see Materials and Methods for details) with an expression construct in which *mHym301A* including the sequence encoding the signal peptide was fused in frame to enhanced green fluorescent protein (eGFP) and under the control of the *Hydra* β-actin promoter (Figure 4A). In transgenic animals producing mHym301A:eGFP, secretion of the fusion protein was detectable during embryonic development in the egg shell of the embryo starting 3 d after injection (Figure 4B and 4C). After hatching, fully transgenic animals producing mHym301A:eGFP in all their ectodermal epithelial cells were generated by asexual propagation of founder polyps (Figure 4D–4F). Figure 4G indicates that, in nontransgenic H. magnipapillata polyps, *mHym301A* transcripts are restricted to the head tissue and absent in tentacle cells. Transgenic line H. vulgaris AEP^A14^ was used in all further experiments. As a control, a transgenic line was produced (Ecto-1) using the same construct without the sequences encoding signal peptide and the mHym301A sequence. As shown in Figure 4I, the mHym301A:eGFP fusion protein is visually detectable by fluorescence microscopy in transgenic animals along the whole body column and in the tentacles. Fluorescence, however, is much weaker than in the control Ecto-1 line (Figure 4H). Confocal laser scanning microscopy shows (Fig 4J–4L) the presence of the mHym301A:eGFP fusion protein in secretory vesicles of ectodermal epithelial cells. In contrast, the eGFP protein in control Ecto-1 animals is not secreted and resides in the cytoplasm of epithelial cells (Figure S2). Thus, by a gain-of-function approach we generated H. vulgaris AEP transgenic animals that strongly overexpress the *H. magnipapillata mHym301A* gene in all ectodermal epithelial cells, including the tentacle cells. In these transgenic H. vulgaris AEP animals, the level of *Hym301* expression is not only greatly enhanced compared with wild-type H. vulgaris AEP, but expression also extends to the tentacles—thereby mimicking *oHym301A* transcript localization in H. oligactis.

Do these perturbations in *Hym301* expression affect morphological traits of transgenic H. vulgaris AEP^A14^ polyps? To address this issue, we compared tentacle development in transgenic animals with that in wild-type H. vulgaris AEP, transgenic H. vulgaris AEP line Ecto-1, and wild-type H. magnipapillata and H. oligactis.

### *mHym301A* Overexpression Affects Both the Speed of Tentacle Regeneration and the Pattern in Which Tentacles Arise

**Figure 5:**
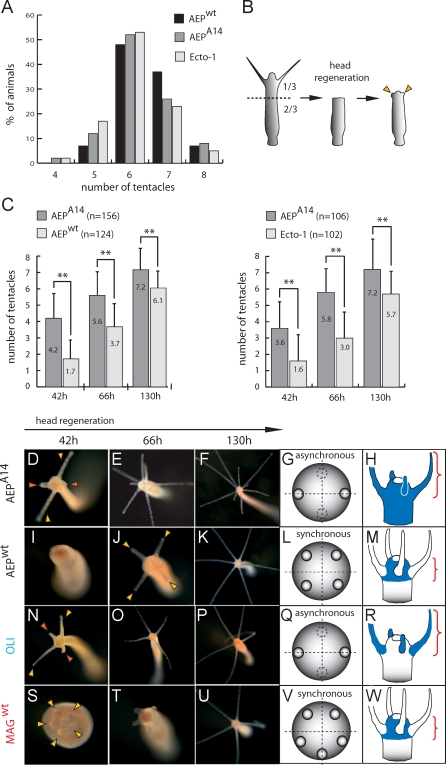
Overexpression of *mHym301A* Alters Timing and Order of Tentacle Development during Regeneration (A) Number of tentacles per polyp in H. vulgaris AEP^wt^, transgenic H. vulgaris AEP^A14^, and transgenic H. vulgaris AEP Ecto-1 lines. In all three lines tentacle number was counted in 100 adult polyps with buds. (B) Experimental scheme for the regeneration experiments. Polyps were cut at 1/3 of the body length. (C) Time kinetics of tentacle formation in transgenic H. vulgaris AEP^A14^ compared with that in wild-type H. vulgaris AEP and transgenic H. vulgaris AEP Ecto-1 animals. Mean number of tentacles per polyp in AEP^A14^, AEP^wt^ and Ecto-1 at 42, 66, and 130 h after decapitation (two asterisks, *p* < 0.01 according to ANOVA test). (D–F) In transgenic H. vulgaris AEP^A14^ newly formed tentacles arise asynchronously. (G) Scheme summarizing the order in which tentacles arise in transgenic H. vulgaris AEP^A14^. (H) Scheme depicting the ectopic expression pattern of *mHym301A* in transgenic H. vulgaris AEP^A14^ (see Figure 4F). (I–K) In control H. vulgaris AEP newly formed tentacles arise synchronously and considerably later than in transgenic animals. (L) Scheme summarizing the order in which tentacles arise in wild-type H. vulgaris AEP^wt^. (M) Scheme depicting the expression pattern of *aepHym301A* in H. vulgaris AEP (see Figure 3G). Note the absence of transcripts in tentacles. (N–P) In H. oligactis newly formed tentacles arise asynchronously. (Q) Scheme summarizing the order in which tentacles arise in H. oligactis. (R) Scheme depicting the expression pattern of *oHym301A* in H. oligactis. Note the presence of the transcript in tentacles (see Figure 3E). (S–U) In H. magnipapillata newly formed tentacles arise synchronously. (V) Scheme summarizing the order in which tentacles arise in H. magnipapillata. (W) Scheme depicting the expression pattern of *mHym301A* in H. magnipapillata. Note the absence of transcripts in tentacles (see Figure 3A).

When a hydra's hypostome and tentacles are removed, the animal promptly develops new ones. “Head regeneration” is a morphallactic process involving the reorganization of existing tissue; it follows species-specific rules, with tentacles arising synchronously in H. magnipapillata and H. vulgaris but asynchronously in *H. oligactis.* Intact transgenic H. vulgaris AEP^A14^ animals exhibit normal morphology without any obvious disturbances (Figure 4I). The number of tentacles seems equal in nontransgenic H. vulgaris AEP^wt^, transgenic H. vulgaris AEP^A14^, and control transgenic line Ecto-1 animals (Figure 5A; see also Materials and Methods). Differences, however, become obvious when a polyp is bisected in the body column (Figure 5B) and a head regenerates at the apical end of the lower half within 96 h. Figure 5C indicates that in H. vulgaris AEP^A14^, tentacles begin to appear much earlier and in larger numbers than in nontransgenic H. vulgaris AEP^wt^ or control transgenic line Ecto-1 (Table S4). Forty-two h after bisection, transgenic H. vulgaris AEP^A14^ had 4.2 ± 1.5 or 3.6 ± 1.61 tentacles compared with only 1.71 ± 1.24 in nontransgenic H. vulgaris AEP^wt^ or 1.6 ± 1.64 in the transgenic Ecto-1 strain (Figure 5C). Figure 5D shows a representative transgenic animal, with well developed tentacles 42 h after decapitation; in the nontransgenic control (Figure 5I), tentacle formation is just at its beginning. By 66 h after decapitation, the mean number of tentacles in transgenic H. vulgaris AEP^A14^ was observed (Figure 5C) to vary from 5.6 ± 1.52 to 5.8 ± 1.44 compared with 3.68 ±1.51 in nontransgenic H. vulgaris AEP^wt^ and 3.0 ± 1.64 in the control Ecto-1 line. The difference in tentacle number was maintained until 130 h after bisection (7.2 ± 1.3 in H. vulgaris AEP^A14^ versus 6.1 ± 1.05 and 5.7 ± 1.31 tentacles, respectively, in nontransgenic and Ecto-1 line controls).

When examining regeneration in nontransgenic H. vulgaris AEP^wt^ and transgenic H. vulgaris AEP^A14^, we noticed differences not only in the time kinetics of tentacle development, but also in the order in which tentacles arise on the regenerating tip (Figure 5D–5M). Unexpectedly, in transgenic H. vulgaris AEP^A14^ (Figure 5D–5F), in contrast to nontransgenic H. vulgaris AEP^wt^ (Figure 5I–5K), tentacles arise asynchronously with two tentacles developing first. This mode of tentacle formation is similar to the order in which tentacles arise in regenerating H. oligactis polyps (Figure 5N–5P) and different from the mode of tentacle formation in the closely related species H. magnipapillata (Figure 5S–5U). Thus, overexpression of *mHym301A* in H. vulgaris AEP body column and tentacle cells (Figure 4I) causes reorganization of the order in which tentacles appear, from the H. vulgaris AEP pattern towards the H. oligactis-specific pattern. Given that in transgenic H. vulgaris AEP^A14^ as in H. oligactis polyps, mHym301A protein is present in tentacle cells (Figures 4I, 5H and 5R), these observations suggest that Hym301 genes are involved in determining the symmetry of tentacle formation.

**Figure 6:**
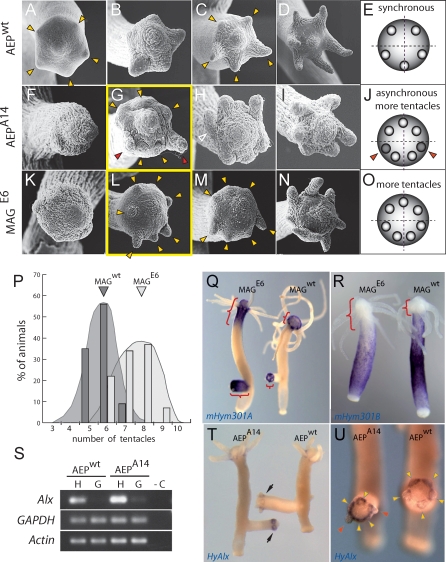
Overexpression of *mHym301A* Alters Tentacle Development during Budding and Mimics a Mutant Phenotype of H. magnipapillata (A–D) Representative scanning electron micrographs of bud evagination in control H. vulgaris AEP^wt^. Yellow arrowheads indicate emerging tentacles. (E) Scheme of tentacle formation during budding in H. vulgaris AEP^wt^. (F–I) Representative scanning electron micrographs of bud evagination in transgenic H. vulgaris AEP^A14^ overexpressing *mHym301A*. Note the slightly staggered order in which tentacles arise (marked with red arrowheads). (J) Scheme of tentacle formation during budding in H. vulgaris AEP^A14^. (K–N) Scheme of tentacle formation during budding in H. magnipapillata mutant E6. Note that more tentacles arise. (O) Scheme of tentacle formation during budding in H. magnipapillata mutant E6. (P) Number of tentacles per polyp in wild-type H. magnipapillata 105 (*n* = 100) and mutant H. magnipapillata E6 (*n* = 100). (Q) Expression of *mHym301A* in mutant E6 and wild-type H. magnipapillata. Note the particularly high level of expression in mutant E6 buds compared with wild type (marked with red brackets). (R) Expression of *mHym301B* in mutant E6 and wild-type H. magnipapillata. (S) RT-PCR analysis indicates higher level of expression of transcription factor *HyAlx* in transgenic H. vulgaris AEP^A14^ overexpressing *mHym301A* than in control H. vulgaris AEP^wt^. H, head region; G, gastric region; –C, negative control. (T) Whole-mount in situ hybridization confirms the expression data in (S) and shows higher level of *HyAlx* transcripts in buds of transgenic H. vulgaris AEP^A14^ than in control buds. (U) Close up of *HyAlx* expression in buds of transgenic H. vulgaris AEP^A14^ and in control H. vulgaris AEP^wt^ buds. Note that, consistent with data shown in (F–I), tentacles in transgenic buds arise asynchronously in contrast to nontransgenic controls (marked with red arrowheads). Fixation conditions and time of development during in situ procedure were identical for mutant E6 and wild-type H. magnipapillata (in Q and R) as well as for AEP^A14^ and AEP^wt^ (in T and U).

To lend further credibility to this hypothesis, we next examined tentacle development during budding, a developmental process by which de novo head formation occurs in an adult hydra (Figure 6). As shown in Figure 6A–6D, in nontransgenic H. vulgaris AEP^wt^ the pattern of tentacle formation is similar to that observed in H. magnipapillata (see Figure 1N–1Q) and different from that in H. oligactis (see Figure 1I–1L). In H. vulgaris AEP^wt^, five tentacle buds appear almost synchronously at the earliest stages of bud formation (arrowheads in Figure 6A) in a regular pentagonal pattern. In contrast, in transgenic H. vulgaris AEP^A14^ overexpressing *mHym301A* (Figure 6F–6I), the tentacles arise in a significantly larger number than in nontransgenic controls and in a slightly staggered sequence. As depicted in Figure 6G and 6H, transgenic H. vulgaris AEP^A14^ develop six to seven tentacle buds in a rather irregular pattern, with some of the tentacles arising even outside the head in the upper part of the body column (open arrowhead in Figure 6H). Given that in adult polyps the number of tentacles is similar (varying from five to eight; Figure 5A) in nontransgenic and transgenic H. vulgaris AEP, the full set of tentacles appears to be established early in transgenic polyps and late (after detachment from the mother polyp) in nontransgenic controls. Taken together, these results substantiate the view that *mHym301A* is a molecule that affects the timing and order in which tentacles arise in *Hydra*.

### Hym301 Expression in a Mutant with Altered Number of Tentacles

To further explore the role of Hym301 in tentacle development, we studied a H. magnipapillata mutant, whose number of tentacles is significantly increased [38]. As shown in Figure 6P, adult polyps of H. magnipapillata line E6 have on average seven to eight tentacles, whereas polyps of the H. magnipapillata wild-type line 105 have five to six tentacles. Analysis of tentacle formation during early stages of budding showed that in the mutant *H. magnipapillata* line E6 tentacles arise earlier and in significantly larger numbers (Figure 6K–6N) than in H. magnipapillata wild-type line 105 (Figure 1N–1Q). In E6 mutant six to seven tentacles appear simultaneously at very early stages of bud development (Figure 6L and 6M), whereas in wild-type H. magnipapillata only four to five tentacles are present at this time point (Figure 1P).

Tentacle development in buds of H. magnipapillata mutant line E6 (Figure 6K–6N) strikingly parallels the pattern of tentacle development in transgenic H. vulgaris AEP^A14^ (Figure 6F–6I). Is Hym301 responsible for the alterations observed in H. magnipapillata mutant line E6? To examine this possibility we investigated the expression of *mHym301A* and *mHym301B* in H. magnipapillata mutant line E6 by in situ hybridization (Figure 6Q and 6R). Given the results described above, we anticipated an increased level of *mHym301A* expression in the mutant line. As predicted, in situ hybridization showed that the expression domain of *mHym301A* (Figure 6Q) in the head is considerably enlarged in the mutant and extends down into the body column. In evaginating buds, the differences in Hym301 expression between wild-type and mutant polyps are even more striking. Whereas in buds of the same developmental stage of wild-type H. magnipapillata line 105 *mHym301A* expression is restricted to the distal part of the evagination, buds in H. magnipapillata mutant line E6 express *mHym301A* throughout the whole cylindrical bud protrusion (Figure 6Q). Interestingly (Figure 6R), the increased expression domain of *mHym301A* is accompanied by reduced expression of *mHym301B*. Taken together, these studies indicate that altered spatiotemporal control of *Hym301* expression strongly correlates with altered tentacle development in H. magnipapillata mutant E6, and suggest that the taxonomically restricted gene family Hym301 is essential in head morphogenesis as it seems to ensure the development of tentacles in a correct number and species-specific order.

How are such novel genes incorporated into conserved signaling pathways? To address this question we examined whether *mHym301A* is interacting with conserved regulatory components such as transcription factor *Aristaless (HyAlx)*. In H. magnipapillata, *HyAlx* is expressed exclusively in the tentacle zone, the lower part of the head from which tentacles emerge. As shown previously by expression and RNA interference (RNAi) analysis, *HyAlx* is directly involved in the specification of tissue for tentacle formation [39]. Figure 6 shows that in nontransgenic H. vulgaris AEP^wt^ (Figure 6S–6U), the expression of *HyAlx* is similar to that described previously [39] in H. magnipapillata. However, in contrast to nontransgenic H. vulgaris AEP^wt^, in transgenic H. vulgaris AEP^A14^, the expression of *HyAlx* is drastically increased (Figure 6S–6U). Reverse transcription PCR (RT-PCR) suggests (Figure 6S) that in polyps overexpressing *mHym301A, HyAlx* transcripts are not only present in higher amounts in the head region compared with wild type, but are detectable even in the gastric region of AEP^A14^ animals. Given that overexpression of *mHym301A* causes an increased expression of *HyAlx*, interaction between novel gene *mHym301A* and a conserved transcription factor seems likely. Interestingly, our expression analysis places *Hym301* upstream of *HyAlx* in the signaling cascade. Previously, Smith et al. have shown [39] that depletion of *HyAlx* RNA drastically delays tentacle appearance in buds, indicating that expression of *HyAlx* is required for this process. We conclude that in transgenic H. vulgaris AEP^A14^ the *mHym301A*-induced increase in the level of *HyAlx* is responsible for the alteration observed in tentacle development compared with controls.

### Silencing of *oHym301A* in H. oligactis Interferes with Tentacle Formation

We have observed drastic disturbances in head morphogenesis, leading to an increased speed of tentacle formation and altered pattern of tentacle distribution in animals overexpressing *mHym301A* (Figures 5C–5M and 6A–6J). We next investigated the role of Hym301 in tentacle formation by silencing *oHym301A* expression in H. oligactis by RNAi.

**Figure 7:**
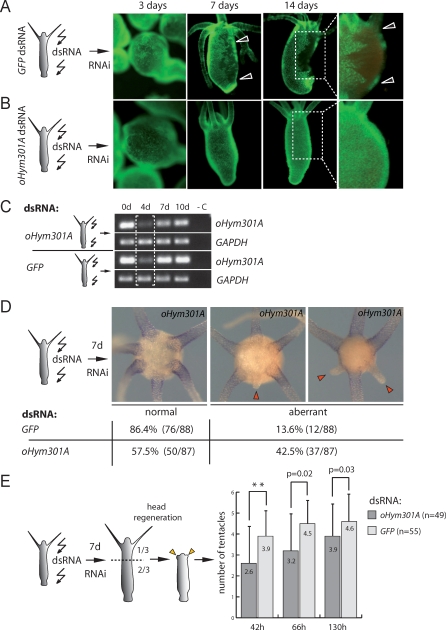
Silencing of *oHym301A* in H. oligactis Interferes with Tentacle Formation (A) Depletion of GFP protein in H. vulgaris AEP Ecto-1 line 3, 7, and 14 d after electroporation with GFP dsRNA. Note dark areas of ectodermal epithelium where GFP transcript and protein are completely depleted. (B) Electroporation of Ecto-1 animals with *oHym301A* dsRNA has no effect on GFP expression. (C) RT-PCR shows depletion of *oHym301A* transcript by RNAi in H. oligactis polyps at various time-points: 0 d, before electroporation and 4–10 d after electroporation; glyceraldehyde 3-phosphate dehydrogenase (GAPDH) was used for equilibration. (D) Disturbed tentacle formation in H. oligactis after electroporation with *oHym301A* dsRNA. In situ hybridization 7 d after electroporation indicates that abnormalities in tentacle length are strongly correlated with the absence of *oHym301A* transcripts. Percentage of animals with normal and aberrant tentacle morphology is shown. Absolute number of experimental animals used is shown in brackets. Three independent RNAi experiments were performed. (E) Time kinetics of tentacle formation in H. oligactis polyps 7 d after electroporation with *oHym301A* or *GFP* dsRNA. Mean number of tentacles per polyp at 42, 66 and 130 h after decapitation (two asterisks, *p* < 0.01 according to ANOVA test).

To optimize the RNAi procedure, we first performed control experiments in which GFP expression was knocked down in H. vulgaris AEP Ecto-1 animals by electroporation of GFP double-stranded RNA (dsRNA). As shown in Figure 7A, introduction of GFP dsRNA causes complete disappearance of GFP protein in large patches of ectodermal epithelial cells covering 10–70% of the polyp's surface. Ecto-1 polyps electroporated with *oHym301A* dsRNA showed no depletion of GFP signal (Figure 7B).

Next we examined the effect of *oHym301A* knock-down on the tentacle formation and morphology of H. oligactis. Polyps were electroporated with *oHym301A* dsRNA. Control polyps were electroporated with GFP dsRNA. The depletion of *oHym301A* was monitored by RT-PCR at various time points and by in situ hybridization 7 d after electroporation. As shown in Figure 7C, by introducing *oHym301A* dsRNA the expression of *oHym301A* was depleted by about 50% (7 d and 10 d in Figure 7C). Electroporation with GFP dsRNA had no effect on the expression of *oHym301A* (Figure 7C). The decrease of Hym301 transcript level 4 d after electroporation in GFP control is due to the loss of tentacles during the electroporation procedure (see Materials and Methods). After electroporation tentacles regenerate within 5 d and in control polyps the normal symmetrical pattern is reestablished.

Intriguingly, in H. oligactis polyps electroporated with *oHym301A* dsRNA, tentacle formation was found to be greatly disturbed. As shown in Figure 7D, 42.5% of polyps electroporated with *oHym301A* dsRNA developed one or two aberrant tentacles, which were considerably shorter than the others (labeled with arrowheads in Figure 7D). In control animals only 13.6% of polyps displayed abnormalities in tentacle morphology. Abnormal length of tentacles strongly correlates with the absence of *oHym301A* transcript as detected by in situ hybridization (Figure 7D). On the basis of the observation shown in Figure 7A, which indicates that RNAi depletion is restricted to patches of cells, we assume that shorter tentacles originate from areas of epithelial cells where *oHym301A* expression was effectively silenced. Taken together, the RNAi experiments indicate that in the absence of *oHym301A* mRNA tentacle development in *Hydra* is retarded.

Head regeneration experiments (Figure 7E) also show that H. oligactis polyps in which the *oHym301A* was silenced by RNAi develop new tentacles considerably more slowly than control polyps that were electroporated with GFP dsRNA (see Table S4). Differences in regeneration kinetics between H. oligactis knock-down and control animals are not as drastic as between animals of H. vulgaris AEP^14^ and Ecto-1 lines (Figure 5C). We assume that the mosaic distribution of cells with complete knock-down of *oHym301A* is the reason for this, as tentacles develop more slowly only in the areas in which *oHym301A* transcript was depleted.

In summary, *oHym301A* expression in tentacles is required for correct development in H. oligactis. Knock-down of *oHym301A* gene does not fully abolish tentacle formation, but slows it down considerably (Figure 7D and 7E). Hence, a “novel” gene belonging to the Hym301 gene family is causally involved in the process of tentacle formation in *Hydra*.

## Discussion

Our results delineate a role for novel gene family Hym301 in tentacle formation in *Hydra*. We show that the species-specific differences in the symmetry of tentacle formation between H. oligactis and H. magnipapillata may be due to the differences in the expression domains of TRGs. Although these results do not determine the precise genetic network responsible for the tentacle formation, they are consistent with previous observations [37,39] and make a significant prediction: conserved regulatory genes and signal transduction cascades alone may not be sufficient to explain the phenotypic differences observed between closely related organisms.

Previous studies have revealed that upstream developmental control mechanisms and regulatory pathways are conserved from Cnidaria to humans [6,7,29–31]. Conserved genetic components and specific changes in *cis*- and *trans*-acting factors were thought to be sufficient for generating novelty [8,15]. So far, the evolutionary significance of TRGs has not been widely recognized [18,19]. However, it has been presumed earlier [4] that at least part of the resolution of the paradox—where the diversity comes from if the genes are highly conserved—may lie in lower levels of conservation of downstream genes. The sequencing of a large number of eukaryotic and bacterial genomes has uncovered an abundance of genes without homologs, classified [19] as TRGs and has shown that new genes have arisen in the genomes of every group of organisms studied so far including humans [40–43]. Here we show that in a basal metazoan group of animals a family of TRGs controls morphological traits in closely related species. The data provide experimental support for the hypothesis [27] that novel genes are involved in specific ecological adaptations that change over time and that such genes serve as the raw material for microevolutionary divergence. The observations also extend earlier findings of an abundance of TRGs in organisms from prokaryotes [18,19] to animals. The observations show that regulatory evolution [8] may act not only by modifying expression domains of conserved genes, but also by spatial and temporal changes in the deployment of TRGs, and that TRGs can be integrated with conserved developmental regulators to form functional signaling cascades.

In *Hydra* it appears that the TRGs belonging to the Hym301 family are specifically required to control the speed of tentacle formation and arrangement of tentacles on buds and regenerating tips. Transgenic manipulations affecting the expression domains of Hym301 genes are responsible for profound effects in tentacle formation, mimicking evolutionary changes. The Hym301 gene family, therefore, is of special importance in *Hydra*. Given that Hym301 genes are without homologs in eukaryotic genomes outside Hydrozoa, they might have been specifically acquired in this animal group. An important step that remains to be demonstrated is the role of natural selection in fine tuning of expression of Hym301 genes or their gene regulators for this lineage-specific adaptation. Tentacles are the only structures allowing *Hydra* polyps to catch and take up food. Differences in their shape, number, and arrangement open different ecological niches and, therefore, are under strict selective constraints. Because pattern formation in *Hydra* is purely morphallactic and the amount of tissue available for the evagination of tentacles is limited, there are only two mechanisms that can produce different arranged tentacles at the developmental level: simultaneous generation of many short tentacles or early generation of few long tentacles before the other tentacles appear. It seems that two subgroups within the genus *Hydra* have adapted different species-specific strategies in this respect and that Hym301 genes play a key role in this morphogenetic process. In H. oligactis, expression of Hym301 genes in the tentacles correlates with formation of two long and functional tentacles before the other tentacles appear. In H. magnipapillata and H. vulgaris, which express Hym301 genes in the tentacle zone but not in the tentacles, four or five short tentacles are built simultaneously. It will be interesting to see whether these rather specific adaptations correlate with differences in the planktonic organisms on which polyps feed in nature.

Differences among *H. oligactis, H. magnipapillata*, and H. vulgaris extend to many other aspects of their morphology and physiology (Figure 1). Therefore, future research on these species may provide novel insights on how TRGs are involved in the evolution of the corresponding adaptive traits. Although it is difficult to generalize from this example, comparing morphogenetic processes in different *Hydra* species seems to promise new perspectives on how nature fine-tunes morphogenesis. Discovering not only the similarities but also the molecular differences between different organisms might yield intriguing clues in the mechanisms responsible for evolutionary changes.

## Materials and Methods

### Animals and culture conditions.

Experiments were carried out with H. oligactis, H. vulgaris strain AEP, H. magnipapillata strain 105, and H. magnipapillata strain E6. Transgenic animals were generated using H. vulgaris strain AEP [44]. Animals where cultured according to standard procedures at 18 °C.

### Molecular techniques.

Nucleic acid isolation, cDNA synthesis, RT-PCR, cloning, and sequencing were done following standard protocols. RT-PCR was performed using the following primer sets: oHYM301A_F(40) and oHYM301A_R(144), AEP301A_F and AEP301A_R, gmHYMA_E1F and gmHYMA_E3R, Alx_F(422) and Alx_R(631) (HyAlx), GAPDH_F and GAPDG_R (GAPDH), ACTIN35 and ACTIN34 (β-actin). Sequencing was done with Li-COR 4200 and Li-COR 4300 sequencers and manually verified using e-Seq V2.0 and e-Seq V3.0 software. Primer sequences are shown in Table S5.

### SSH and cDNA libraries.

For SSH, double-stranded cDNA was synthesized using 2 μg mRNA from H. oligactis and H. magnipapillata strain 105 polyps. SSH was performed in both directions using PCR-Select cDNA Subtraction Kit (Clontech) according to the manufacturer's protocol. Two subtractive libraries were generated: Kiel 6, enriched for H. oligactis specific transcripts and Kiel 7, enriched for H. magnipapillata specific transcripts (see Figure 2). cDNAs were cloned into pGEM-T vector (Promega) and transformed into DH5α Escherichia coli cells. Bacterial clones were picked into 384-well plates using Q-Pix robot and plasmid inserts were sequenced at the Washington University Genome Sequencing Centre (St. Louis, Missouri, United States). Raw sequences were submitted to NCBI (http://www.ncbi.nlm.nih.gov/) under accession numbers CV284311–CV284646 and CV2845050–CV285735 (Kiel 6) and CV284048–CV284310, CV284647–CV285049, and CV285736–CV286173 (Kiel 7).

### Isolation of Hym301-like genes from H. magnipapillata, H. oligactis, and H. vulgaris AEP.

To identify all Hym301-like genes in H. magnipapillata, we searched *Hydra* ESTs at http://blast.ncbi.nlm.nih.gov/Blast.cgi (about 170,000 sequences) and single whole-genome shotgun reads deposited at CompaGen server in Kiel (http://www.compagen.org) using translated BLAST and the mHym301A and mHym301B protein sequences as queries. To isolate Hym301-like genes in H. oligactis we screened macroarrays containing 30,000 cDNA clones with the mixture of *mHym301A*, *mHym301B* and *oHym301A* probes using low stringency washing conditions. Out of eight positive clones, four were identical to the *oHym301A* cDNA already known from the SSH library, two represented transcripts of a new member of a gene family designated *oHym301B*, and the other two clones contained sequences not related to Hym301. To isolate Hym301-like genes in H. vulgaris AEP, we performed 3′ RACE PCR according to the previously published method using primer oHym301_F(32) (Table S5) directed against sequence highly conserved in both H. oligactis and H. magnipapillata. In three independent RACE experiments only one type of Hym301-related cDNA was identified, designated later as *aepHym301A*. Sequences of Hym301 genes were submitted to NCBI under accession numbers EU787492–EU787498.

### Gene expression analysis.

For assessment of gene expression, whole-mount in situ hybridization was carried out as described previously [45].

### Generation of transgenic H. vulgaris AEP expressing mHym301A:eGFP.

Transgenic founder polyps overexpressing the mHym301A:eGFP fusion protein under control of the β-actin promoter (construct ligE) were produced at the University of Kiel Transgenic *Hydra* Facility (http://www.transgenic-hydra.org/). Briefly, a 291 bp fragment of *mHym301A* coding for the full-length protein including the signal peptide was amplified from H. magnipapillata strain 105 cDNA using Platinum High Fidelity Taq polymerase (Invitrogen) and primers Hym301_F(2)Pst and Hym301_R(295)Pst. The cDNA was cloned into the modification of HoTG expression vector using the *Pst*I cutting site (see Figure 4A). The resulting transfection construct was sequenced, plasmid DNA was purified using Qiagen MidiPrep Kit and injected into H. vulgaris AEP embryos as described previously [44]. Embryos began to express the reporter gene 2–3 d after injection. Founder transgenic animals bearing the mHym301A:eGFP construct started to hatch 14 d after microinjection. Out of 24 embryos injected with the LigE construct, two transgenic lines were generated. One of them (line A7) showed stable integration of mHym301A:eGFP in ectodermal and endodermal epithelial cell lineages. Another (line A14) showed integration of the construct in ectodermal epithelial cells only. Initial founder transgenic animals of A14 line were further expanded into a mass culture by clonal propagation by budding and used in all further experiments.

### Sequence analysis.

TIGR Indices Clustering Tools [46] were used for clustering the sequences of Kiel 6 and Kiel 7 libraries. Nucleotide and translated BLAST engines at the NCBI server (http://blast.ncbi.nlm.nih.gov/Blast.cgi) were used for homology searches in public databases. The Seqtools program (S.W. Rasmussen, http://www.seqtools.dk) was used for sequence analysis and batch BLASTX and BLASTN searches. DNA and protein sequences were aligned with DnaMan Version 4.12. Phylogenetic tree analysis was performed by the neighbor-joining method with standard settings and a bootstrap value of 1,000 using the Mega 3.1 software. Local assemblies of H. magnipapillata genome were performed manually using single whole genome shotgun reads deposited at NCBI trace archive (http://www.ncbi.nlm.nih.gov/Traces/) and the Compagen server in Kiel (http://www.compagen.org).

### Microscopic analysis.

Fluorescent images were taken on a Zeiss Axioscope fluorescence microscope with an Axiocam (Zeiss) digital camera. Confocal laser microscopy was done using a LEICA TCS SP1 CLS microscope. A Zeiss S420 microscope was used for scanning electron microscopy.

### Gene silencing by RNAi in *Hydra.*

Double-stranded RNAs for *GFP* and *oHym301A* were synthesized using a MEGAscript RNAi Kit (Ambion). DNA templates for RNA synthesis were generated by PCR using primers T7_GFP_F, T7_GFP_R, o301Ai_T7_F, o301Ai_T7_R (see Table S5). DsRNA was introduced into *Hydra* polyps by electroporation as described previously [47] with minor modifications. For each experiment 30–60 polyps were placed into chilled electroporation cuvettes with a 4 mm gap (Peqlab). Polyps were washed twice with 1 ml sterile ice-cold Millipore water. Electroporation was carried out in 200 μl water containing 15 μg dsRNA. DsRNA was added to polyps just before electroporation and cuvettes were briefly shaken to insure regular distribution of animals and dsRNA. Bio-Rad Gene Pulser (Bio-Rad) was adjusted to an electric field strength of 0.5 kV/cm and 50-μF capacitance. Polyps were electroporated two times successively with each pulse lasting for about 10–12 ms. Immediately after the second pulse polyps were transferred into 10 ml ice-cold hydra medium that was supplemented with 20% hyperosmotic dissociation medium (DM) containing 6 mM CaCl_2_, 1.2 mM MgSO_4_, 3.6 mM KCl, 12.5 mM N-tris-[hydroxymethyl] methyl-2 aminoethanesulfonic acid, 6 mM sodium pyruvate, 6 mM sodium citrate, 6.0 mM glucose, and 50 mg/ml rifampicin, pH 6.9. Electroporation causes cell loss and tissue damage with all the animals losing their tentacles (note the absence of tentacles in Figure 7A and 7B, 3 d after electroporation). To facilitate recovery, polyps were kept at 10 °C for up to 3 d. Hydra medium supplemented with 20% DM was exchanged every 12 h; viable polyps were separated from cell debris and transferred into new Petri dishes. Thirty-six h after electroporation the medium was exchanged for standard hydra medium. Five d after electroporation polyps were fully recovered and used for experimentation. Survival rate for the electroporated polyps varied between 40% and 60%.

Ecto-1 transgenic line which expresses GFP in all ectodermal epithelial cells was used to directly visualize RNAi in vivo. Electroporation of Ecto-1 animals with GFP dsRNA allowed for the first time to observe the disappearance of the RNAi target protein in living *Hydra* polyps. Because of the high stability of the GFP protein, the first areas of completely GFP-negative epithelial cells become clearly visible 5 d after electroporation. Complete down-regulation of the target gene was observed in areas of cells that, in the best case, covered about 70% of the polyp surface. The RNAi effect is stable for the period of over 14 d (Figure 7A).

## Supporting Information

Figure S1 Analysis of the SSH Library Enriched for *H. oligactis–*Specific Transcripts (A) BLASTN search of H. oligactis clusters and singletons (Kiel 6 library) against all H. magnipapillata ESTs. The cut-off value was set to *E* < 1e–10. Pie diagram shows the distribution of the SSH clusters and singletons according to their sequence identity to H. magnipapillata ESTs: 42% have sequence identity of 90–100% (possible false positives); 39% have sequence identity of 80–90% (transcripts of highly diverged genes); 19% have sequence identity below 80% (putative H. oligactis-specific genes).
(B–G) Whole mount in situ hybridization showing differences in the expression patterns of homologous genes between H. oligactis and H. magnipapillata. (B) A member of the Kazal-type family of proteinase inhibitors (CL67 in Table S1, CV284473) is not expressed in the foot (stalk) of H. oligactis, whereas its closest homolog (C) in H. magnipapillata (CL42 in Table S2, CV284784) is expressed in gland cells all over the body column, including the foot. (D,E) A novel secreted protein is expressed in ectodermal epithelial cells exclusively in the stalk (D) of H. oligactis (CL225 in Table S1, EU787491). This gene seems to be taxonomically restricted to H. oligactis as (1) no signal could be detected by in situ hybridization in H. magnipapillata (E) and (2) no homologous sequences are present among the 170,000 ESTs and in the genome of H. magnipapillata. (F,G) *Hydra*-specific genes identified by our approach include also genes expressed in developing nematocytes. Minicollagen-15 (EF624460)-like genes in H. oligactis (CL140, CV285608) and H. magnipapillata (CL173, EU787490) show different expression domains. The minicollagen-15 transcript is absent in the stalk structure of H. oligactis (F) and expands much further down to the foot in H. magnipapillata (G).
(274 KB PDF)

Figure S2 Ectodermal Epithelial Cells of H. vulgaris AEP and Cellular Localization of eGFP and mHym301A:eGFP in Transgenic Lines Ecto-1 and H. vulgaris AEP^A14^
Semi-thin section in H. vulgaris AEP shows localization of vesicles on the periphery of ectodermal epithelial cells. In transgenic H. vulgaris AEP^A14^ polyps the mHym301A:eGFP fusion protein is located in vesicles in ectodermal epithelial cells. In control Ecto-1 polyps transformed with the same expression construct but lacking the mHym301A sequence, the eGFP reporter protein is localized in the cytoplasm. Vesicles appear as black holes on the green background.
(676 KB PDF)

Table S1 Kiel 6 BLASTX Search against the Nonredundant NCBI General information about clusters and singletons of H. oligactis-specific cDNA library (Kiel 6). Results of the BLASTX search against the nonredundant NCBI database. Sequences with *E* value ≥ 1e–5 were referred to as having no significant similarity to the proteins in NCBI database (potential TRGs). The clusters were numbered according to the amount of ESTs comprising them, with cluster 01 (CL01CONTIG1) being the largest. The consensus cluster sequences and singleton sequences are stored as a multi-sequence file (FASTA format) at the COMPAGEN server (http://compagen.zoologie.uni-kiel.de/retrieve.htm).
(78 KB PDF)

Table S2 Kiel 7 BLASTX Search against the Nonredundant NCBI General information about clusters and singletons of H. magnipapillata-specific cDNA library (Kiel 7). Results of the BLASTX search against the nonredundant NCBI database. Sequences with *E* value ≥ 1e–5 were referred to as having no significant similarity to the proteins in NCBI database (potential TRGs). The clusters were numbered according to the amount of ESTs comprising them, with cluster 01 (CL01CONTIG1) being the largest. The consensus cluster sequences and singleton sequences are stored as a multi-sequence file (FASTA format) at the COMPAGEN server (http://compagen.zoologie.uni-kiel.de/retrieve.htm).
(80 KB PDF)

Table S3 Kiel 6 BLASTN Search against *Hydra* ESTs Results of the BLASTN search of H. oligactis clusters and singletons from the Kiel 6 library against all H. magnipapillata ESTs. The cut-off value was set to *E* < 1e–10. Sequences with *E* value ≥ 1e–10 were referred to as having no significant similarity among H. magnipapillata ESTs (transcripts of potential H. oligactis-specific genes).
(68 KB PDF)

Table S4 Regeneration Experiments Mean number of tentacles per polyp at 42, 66, and 130 h after decapitation (mean ± standard deviation) in independent experiments (I, II, III, etc.).
(15 KB PDF)

Table S5 Oligonucleotide Primer Sequences (7 KB PDF)

## Abbreviations

dsRNA: double-stranded RNA
eGFP: enhanced green fluorescent protein
EST: expressed sequence tag
GAPDH: glyceraldehyde 3-phosphate dehydrogenase
GFP: green fluorescent protein
RNAi: RNA interference
SSH: suppression subtractive hybridization
TRG: taxonomically restricted gene

## References

[pbio-0060278-b001] Gould SJ (1977). Ever since Darwin.

[pbio-0060278-b002] Raff RA, Kaufman TC (1983). Embryos, genes and evolution: the developmental genetic basis of evolutionary change.

[pbio-0060278-b003] Raff R (1996). The shape of life.

[pbio-0060278-b004] Arthur W (2002). The emerging conceptual framework of evolutionary developmental biology. Nature.

[pbio-0060278-b005] Hall BK (1992). Evolutionary developmental biology.

[pbio-0060278-b006] Kortschak RD, Samuel G, Saint R, Miller DJ (2003). EST analysis of the cnidarian Acropora millepora reveals extensive gene loss and rapid sequence divergence in the model invertebrates. Curr Biol.

[pbio-0060278-b007] Putnam NH, Srivastava M, Hellsten U, Dirks B, Chapman J (2007). Sea anemone genome reveals ancestral eumetazoan gene repertoire and genomic organization. Science.

[pbio-0060278-b008] Prud'homme B, Gompel N, Carroll SB (2007). Emerging principles of regulatory evolution. Proc Natl Acad Sci USA.

[pbio-0060278-b009] Gompel N, Prud'homme B, Wittkopp PJ, Kassner VA, Carroll SB (2005). Chance caught on the wing: cis-regulatory evolution and the origin of pigment patterns in *Drosophila*. Nature.

[pbio-0060278-b010] Jeong S, Rokas A, Carroll SB (2006). Regulation of body pigmentation by the Abdominal-B Hox protein and its gain and loss in *Drosophila* evolution. Cell.

[pbio-0060278-b011] Prud'homme B, Gompel N, Rokas A, Kassner VA, Williams TM (2006). Repeated morphological evolution through cis-regulatory changes in a pleiotropic gene. Nature.

[pbio-0060278-b012] Abzhanov A, Protas M, Grant BR, Grant PR, Tabin CJ (2004). Bmp4 and morphological variation of beaks in Darwin's finches. Science.

[pbio-0060278-b013] Abzhanov A, Kuo WP, Hartmann C, Grant BR, Grant PR, Tabin CJ (2006). The calmodulin pathway and evolution of elongated beak morphology in Darwin's finches. Nature.

[pbio-0060278-b014] Wu P, Jiang TX, Suksaweang S, Widelitz RB, Chuong CM (2004). Molecular shaping of the beak. Science.

[pbio-0060278-b015] Carroll SB, Grenier JK, Weatherbee SD, Carroll S (2001). From DNA to diversity.

[pbio-0060278-b016] Fischer D, Eisenberg D (1999). Finding families for genomic ORFans. Bioinformatics.

[pbio-0060278-b017] Rubin GM, Yandell MD, Wortman JR, Gabor Miklos GL, Nelson CR (2000). Comparative genomics of the eukaryotes. Science.

[pbio-0060278-b018] Wilson GA, Feil EJ, Lilley AK, Field D (2007). Large-scale comparative genomic ranking of taxonomically restricted genes (TRGs) in bacterial and archaeal genomes. PLoS ONE.

[pbio-0060278-b019] Wilson GA, Bertrand N, Patel Y, Hughes JB, Feil EJ, Field D (2005). Orphans as taxonomically restricted and ecologically important genes. Microbiology.

[pbio-0060278-b020] Parkinson J, Mitreva M, Whitton C, Thomson M, Daub J (2004). A transcriptomic analysis of the phylum Nematoda. Nat Genet.

[pbio-0060278-b021] Dehal P, Satou Y, Campbell RK, Chapman J, Degnan B (2004). The draft genome of Ciona intestinalis: insights into chordate and vertebrate origins. Science.

[pbio-0060278-b022] Wood V, Gwilliam R, Rajandream MA, Lyne M, Lyne R (2002). The genome sequence of Schizosaccharomyces pombe. Nature.

[pbio-0060278-b023] Gibson MC (2007). Bicoid by the numbers: quantifying a morphogen gradient. Cell.

[pbio-0060278-b024] Roth S (2003). The origin of dorsoventral polarity in *Drosophila*. Phil Trans R Soc Lond B Biol Sci.

[pbio-0060278-b025] Clark AG, Eisen MB, Smith DR, Bergman CM, Drosophila 12 Genomes Consortium, (2007). Evolution of genes and genomes on the *Drosophila* phylogeny. *Nature*.

[pbio-0060278-b026] Richards S, Gibbs RA, Weinstock GM, Brown SJ, *Tribolium* Genome Sequencing Consortium, (2008). The genome of the model beetle and pest Tribolium castaneum. Nature.

[pbio-0060278-b027] Domazet-Loso T, Tautz D (2003). An evolutionary analysis of orphan genes in *Drosophila*. Genome Res.

[pbio-0060278-b028] Bosch TCG (2007). Why polyps regenerate and we don't: towards a cellular and molecular framework for *Hydra* regeneration. Dev Biol.

[pbio-0060278-b029] Miller DJ, Ball EE, Technau U (2005). Cnidarians and ancestral genetic complexity in the animal kingdom. Trends Genet.

[pbio-0060278-b030] Technau U, Rudd S, Maxwell P, Gordon PM, Saina M (2005). Maintenance of ancestral complexity and non-metazoan genes in two basal cnidarians. Trends Genet.

[pbio-0060278-b031] Miller DJ, Hemmrich G, Ball EE, Hayward DC, Khalturin K (2007). The innate immune repertoire in cnidaria – ancestral complexity and stochastic gene loss. Genome Biol.

[pbio-0060278-b032] Bosch TCG, Khalturin K (2002). Patterning and cell differentiation in *Hydra*: novel genes and the limits to conservation. Can J Zool.

[pbio-0060278-b033] Campbell RD, Lenhoff HM (1983). Identifying *Hydra* species. *Hydra* Research Methods.

[pbio-0060278-b034] Hemmrich G, Anokhin B, Zacharias H, Bosch TCG (2007). Molecular phylogenetics in *Hydra*, a classical model in evolutionary developmental biology. Mol Phyl Evol.

[pbio-0060278-b035] Koizumi O (2007). Nerve ring of the hypostome in hydra: is it an origin of the central nervous system of bilaterian animals. Brain Behav Evol.

[pbio-0060278-b036] Takahashi T, Muneoka Y, Lohmann J, Lopez de Haro MS, Solleder G (1997). Systematic isolation of peptide signal molecules regulating development in hydra: LWamide and PW families. Proc Natl Acad Sci USA.

[pbio-0060278-b037] Takahashi T, Hatta M, Yum S, Gee L, Ohtani M (2005). Hym-301, a novel peptide, regulates the number of tentacles formed in hydra. Development.

[pbio-0060278-b038] Sugiyama T, Fujisawa T (1977). Genetic analysis of developmental mechanisms in hydra. I. Sexual reproduction of Hydra magnipapillata and isolation of mutants. Dev Growth Differ.

[pbio-0060278-b039] Smith KM, Gee L, Bode HR (2000). *HyAlx*, an aristaless-related gene, is involved in tentacle formation in hydra. Development.

[pbio-0060278-b040] Emerson JJ, Kaessmann H, Betran E, Long M (2004). Extensive gene traffic on the mammalian X chromosome. Science.

[pbio-0060278-b041] Betran E, Bai Y, Motiwale M (2006). Fast protein evolution and germ line expression of a *Drosophila* parental gene and its young retroposed paralog. Mol Biol Evol.

[pbio-0060278-b042] Wang W, Zheng H, Fan C, Li J, Shi J (2006). High rate of chimeric gene origination by retroposition in plant genomes. Plant Cell.

[pbio-0060278-b043] Wang HC, Hickey DA (2007). Rapid divergence of codon usage patterns within the rice genome. BMC Evol Biol 7 Suppl.

[pbio-0060278-b044] Wittlieb J, Khalturin K, Lohmann JU, Anton-Erxleben F, Bosch TCG (2006). Transgenic *Hydra* allow in vivo tracking of individual stem cells during morphogenesis. Proc Natl Acad Sci USA.

[pbio-0060278-b045] Khalturin K, Anton-Erxleben F, Milde S, Plötz C, Wittlieb J (2007). Transgenic stem cells in *Hydra* reveal an early evolutionary origin for key elements controlling self-renewal and differentiation. Dev Biol.

[pbio-0060278-b046] Pertea G, Huang X, Liang F, Antonescu V, Sultana R (2003). TIGR Gene Indices clustering tools (TGICL): a software system for fast clustering of large EST datasets. Bioinformatics.

[pbio-0060278-b047] Lohmann JU, Endl I, Bosch TCG (1999). Silencing of developmental genes in *Hydra*. Dev Biol.

