# Supplementary figures and images for: A Novel Gene Family Controls Species-Specific Morphological Traits in Hydra
Source: PLoS Biol. 2008 Nov 18;6(11):e278. doi: 10.1371/journal.pbio.0060278 (PMC2586386; doi:10.1371/journal.pbio.0060278)

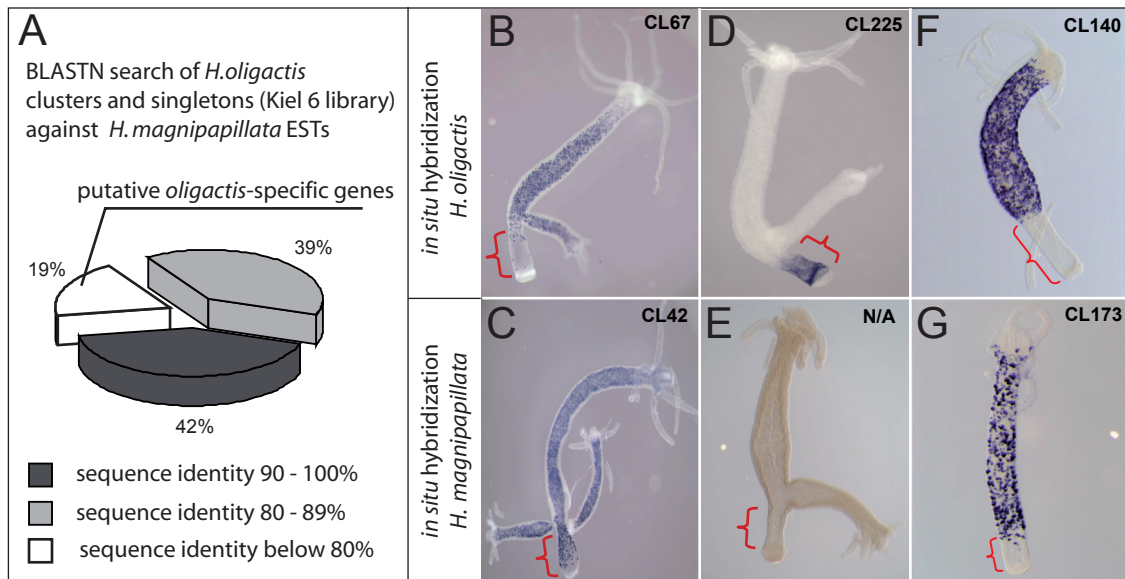

Supplement: Figure S1 — (A) BLASTN search of H. oligactis clusters and singletons (Kiel 6 library) against all H. magnipapillata ESTs. The cut-off value was set to E < 1e–10. Pie diagram shows the distribution of the SSH clusters and singletons according to their sequence identity to H. magnipapillata ESTs: 42% have sequence identity of 90–100% (possible false positives); 39% have sequence identity of 80–90% (transcripts of highly diverged genes); 19% have sequence identity below 80% (putative H. oligactis-specific genes). (B–G) Whole mount in situ hybridization showing differences in the expression patterns of homologous genes between H. oligactis and H. magnipapillata. (B) A member of the Kazal-type family of proteinase inhibitors (CL67 in Table S1, CV284473) is not expressed in the foot (stalk) of H. oligactis, whereas its closest homolog (C) in H. magnipapillata (CL42 in Table S2, CV284784) is expressed in gland cells all over the body column, including the foot. (D,E) A novel secreted protein is expressed in ectodermal epithelial cells exclusively in the stalk (D) of H. oligactis (CL225 in Table S1, EU787491). This gene seems to be taxonomically restricted to H. oligactis as (1) no signal could be detected by in situ hybridization in H. magnipapillata (E) and (2) no homologous sequences are present among the 170,000 ESTs and in the genome of H. magnipapillata. (F,G) Hydra-specific genes identified by our approach include also genes expressed in developing nematocytes. Minicollagen-15 (EF624460)-like genes in H. oligactis (CL140, CV285608) and H. magnipapillata (CL173, EU787490) show different expression domains. The minicollagen-15 transcript is absent in the stalk structure of H. oligactis (F) and expands much further down to the foot in H. magnipapillata (G). (274 KB PDF) [file pbio.0060278.sg001.pdf]

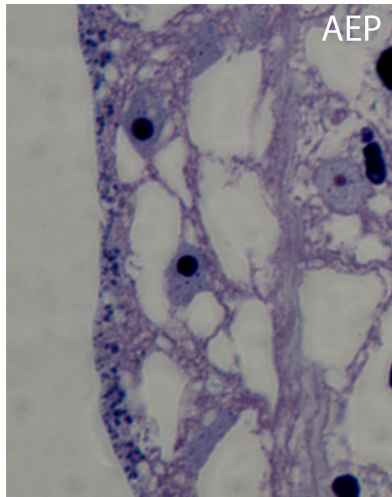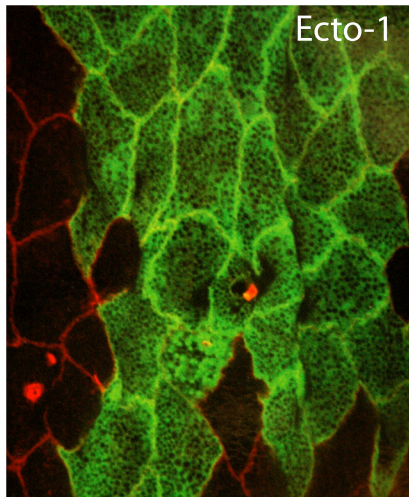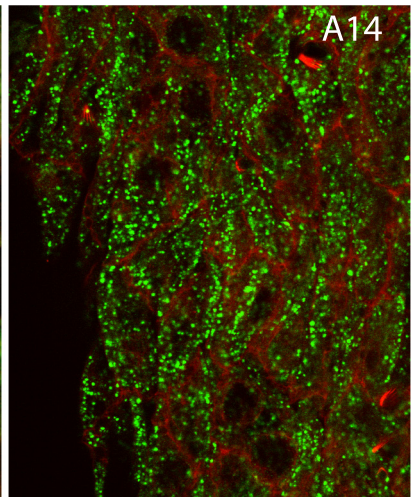

Supplement: Figure S2 — Semi-thin section in H. vulgaris AEP shows localization of vesicles on the periphery of ectodermal epithelial cells. In transgenic H. vulgaris AEPA14 polyps the mHym301A:eGFP fusion protein is located in vesicles in ectodermal epithelial cells. In control Ecto-1 polyps transformed with the same expression construct but lacking the mHym301A sequence, the eGFP reporter protein is localized in the cytoplasm. Vesicles appear as black holes on the green background. (676 KB PDF) [file pbio.0060278.sg002.pdf]
