# Supplementary material for: A Novel Gene Family Controls Species-Specific Morphological Traits in Hydra
Source: PLoS Biol. 2008 Nov 18;6(11):e278. doi: 10.1371/journal.pbio.0060278 (PMC2586386; doi:10.1371/journal.pbio.0060278)
Supplement: Table S1 — General information about clusters and singletons of H. oligactis-specific cDNA library (Kiel 6). Results of the BLASTX search against the nonredundant NCBI database. Sequences with E value ≥ 1e–5 were referred to as having no significant similarity to the proteins in NCBI database (potential TRGs). The clusters were numbered according to the amount of ESTs comprising them, with cluster 01 (CL01CONTIG1) being the largest. The consensus cluster sequences and singleton sequences are stored as a multi-sequence file (FASTA format) at the COMPAGEN server (http://compagen.zoologie.uni-kiel.de/retrieve.htm). (78 KB PDF) [file pbio.0060278.st001.pdf]

Table S1

KIEL 6 (*Hydra oligactis* specific) SSH library. Results of BLASTX search against non-redundant NCBI database

General information about clusters and singletons of *H. oligactis* -specific cDNA library (Kiel 6).
Results of the BLASTX search against non-redundant NCBI database. Sequences with E value ≥ 1e-5 were referred to as having no significant homology to the proteins in NCBI database (potential taxon restricted genes, TGRs).
The clusters were numbered according to the amount of ESTs comprising them, with cluster 01 (CL01CONTIG1) being the largest.
The consensus cluster sequences and singleton sequences are stored as multi-sequence file (FASTA format) at the COMPAGEN server (http://compagen.zoologie.uni-kiel.de/retrieve.htm).

Pct Idnt, % identity between *H.oligactis* amino acid sequence and the best BLASTX match from NCBI
Length (bp), length of consensus cluster sequence or singleton in base pares

N	Name	Best blast match (with E-value < 1e-5)	Accession number	Expect	Pct Idnt	Ratio Idnt
1	CL1CONTIG1	GB|ABC25029.1| ferritin [Hydra vulgaris]	ABC25029.1	9,00E-84	96	153/159
2	CL2CONTIG1	no blast match			0	
3	CL3CONTIG1	EMB|CAM36311.1| hypothetical protein [Thermobia domestica]	CAM36311.1	6,00E-09	62	31/50
4	CL3CONTIG2	EMB|CAM36311.1| hypothetical protein [Thermobia domestica]	CAM36311.1	9,00E-08	60	30/50
5	CL3CONTIG3	no blast match			0	
6	CL4CONTIG1	no blast match			0	
7	CL5CONTIG1	no blast match			0	
8	CL6CONTIG1	GB|AAX48872.1| L37a [Suberites domuncula]	AAX48872.1	9,00E-38	79	73/92
9	CL7CONTIG1	DBJ|BAB33421.1| putative senescence-associated protein [Pisum sa...	BAB33421.1	8,00E-09	73	30/41
10	CL8CONTIG1	REF|XP_001495410.1| PREDICTED: similar to ribosomal protein S5 [...	XP_001495410.1	4,00E-52	86	99/115
11	CL9CONTIG1	no blast match			0	
12	CL10CONTIG1	GB|AAH53776.1| MGC64312 protein [Xenopus laevis]	AAH53776.1	1,00E-19	78	44/56
13	CL11CONTIG1	GB|AAX30301.1| unknown [Schistosoma japonicum]	AAX30301.1	4,00E-22	89	49/55
14	CL12CONTIG1	GB|AAY66953.1| ribosomal protein L36 [Ixodes scapularis]	AAY66953.1	5,00E-22	66	48/72
15	CL13CONTIG1	GB|ABD04168.1| activating transcription factor 4-like protein [A...	ABD04168.1	1,00E-11	34	40/115
16	CL14CONTIG1	REF|NP_001041438.1| hypothetical protein LOC503325 [Rattus norve...	NP_001041438.1	6,00E-11	58	25/43
17	CL15CONTIG1	REF|XP_392691.2| PREDICTED: similar to Elongation factor 2 (EF-2...	XP_392691.2	9,00E-96	77	161/208
18	CL16CONTIG1	EMB|CAJ80765.2| thrombospondin type 1 repeat-containing protein ...	CAJ80765.2	4,00E-18	47	44/92
19	CL17CONTIG1	REF|XP_781777.1| PREDICTED: similar to Ribosomal protein S2 [Str...	XP_781777.1	4,00E-56	83	104/125
20	CL18CONTIG1	no blast match			0	
21	CL18CONTIG2	no blast match			0	
22	CL19CONTIG1	REF|NP_001080051.1| glucose regulated protein, 58kDa [Xenopus la...	NP_001080051.1	1,00E-12	61	36/59
23	CL20CONTIG1	no blast match			0	
24	CL21CONTIG1	GB|AAN05584.1| ribosomal protein L30 [Argopecten irradians]	AAN05584.1	3,00E-31	82	68/82
25	CL22CONTIG1	no blast match			0	
26	CL23CONTIG1	no blast match			0	
27	CL24CONTIG1	REF|XP_780122.1| PREDICTED: hypothetical protein isoform 1 [Stro...	XP_780122.1	5,00E-28	65	54/82
28	CL25CONTIG1	REF|XP_690132.2| PREDICTED: hypothetical protein LOC325699 [Dani...	XP_690132.2	1,00E-21	42	69/163
29	CL25CONTIG2	REF|XP_690132.2| PREDICTED: hypothetical protein LOC325699 [Dani...	XP_690132.2	2,00E-17	39	54/136
30	CL26CONTIG1	REF|NP_001016282.1| heat shock protein 90kDa alpha (cytosolic), ...	NP_001016282.1	4,00E-39	70	78/111
31	CL27CONTIG1	no blast match			0	
32	CL28CONTIG1	GB|AAR31143.1| zebra precursor; HyZebra [Hydra magnipapillata]	AAR31143.1	2,00E-23	44	48/109
33	CL29CONTIG1	REF|XP_001200730.1| PREDICTED: hypothetical protein [Strongyloce...	XP_001200730.1	1,00E-13	92	38/41
34	CL30CONTIG1	no blast match			0	
35	CL31CONTIG1	no blast match			0	
36	CL32CONTIG1	REF|XP_001147795.1| PREDICTED: similar to Ribosomal protein S6 [...	XP_001147795.1	3,00E-39	80	76/95
37	CL33CONTIG1	REF|XP_971685.1| PREDICTED: similar to CG8983-PA, isoform A [Tri...	XP_971685.1	3,00E-97	50	185/368
38	CL34CONTIG1	no blast match			0	
39	CL35CONTIG1	GB|ABC25034.1| ribosomal protein S3 [Hydra vulgaris]	ABC25034.1	4,00E-23	96	50/52
40	CL36CONTIG1	GB|AAX48848.1| L15 [Suberites domuncula]	AAX48848.1	3,00E-43	82	83/101
41	CL37CONTIG1	GB|AAB01531.1| cyclophilin-A >gi|1588025|prf||2207414A cyclophilin	AAB01531.1	1,00E-42	92	81/88
42	CL38CONTIG1	PDB|2IY1|B Chain B, Senp1 (Mutant) Full Length Sumo1 >gi|1147948...	2IY1	6,00E-18	68	44/64
43	CL39CONTIG1	SP|P17126|ACT_HYDAT Actin, non-muscle 6.2 >gi|159254|gb|AAA29205...	P17126	1,00E-80	99	146/147
44	CL40CONTIG1	DBJ|BAA94696.1| calmodulin [Chara corallina] >gi|7594879|dbj|BAA...	BAA94696.1	4,00E-20	94	48/51
45	CL41CONTIG1	REF|NP_001080379.1| cytochrome c-1 [Xenopus laevis] >gi|28175293...	NP_001080379.1	8,00E-20	66	44/66
46	CL42CONTIG1	no blast match			0	
47	CL43CONTIG1	REF|XP_001487046.1| hypothetical protein PGUG_00423 [Pichia guil...	XP_001487046.1	2,00E-09	41	33/80
48	CL44CONTIG1	no blast match			0	
49	CL45CONTIG1	DBJ|BAF37942.1| Similar to ribosomal protein S23 [Oncorhynchus m...	BAF37942.1	1,00E-48	93	93/99
50	CL46CONTIG1	no blast match			0	
51	CL47CONTIG1	no blast match			0	
52	CL48CONTIG1	GB|EAT45815.1| conserved hypothetical protein [Aedes aegypti]	EAT45815.1	7,00E-27	56	56/99
53	CL49CONTIG1	no blast match			0	
54	CL50CONTIG1	REF|XP_796390.1| PREDICTED: hypothetical protein [Strongylocentr...	XP_796390.1	2,00E-10	88	31/35
55	CL51CONTIG1	no blast match			0	
56	CL52CONTIG1	GB|AAX11340.1| ribosomal protein L28 [Haliotis asinina]	AAX11340.1	6,00E-13	56	41/73
57	CL53CONTIG1	SP|P49395|RS3A_APLCA 40S ribosomal protein S3a (Lysine-rich prot...	P49395	1,00E-46	69	90/130
58	CL54CONTIG1	GB|ABA42878.1| small heat shock protein [uncultured cnidarian]	ABA42878.1	2,00E-15	37	52/137
59	CL55CONTIG1	no blast match			0	
60	CL55CONTIG2	no blast match			0	
61	CL56CONTIG1	REF|XP_554103.2| ENSANGP00000025683 [Anopheles gambiae str. PEST...	XP_554103.2	3,00E-08	50	28/55
62	CL57CONTIG1	DBJ|BAE71215.1| putative 60S ribosomal protein L1 [Trifolium pra...	BAE71215.1	1,00E-46	57	99/171
63	CL58CONTIG1	no blast match			0	
64	CL59CONTIG1	SP|P46088|GST_OMMSL Glutathione S-transferase (GST class-sigma) ...	P46088	5,00E-08	35	39/111
65	CL60CONTIG1	REF|XP_967635.1| PREDICTED: similar to CG3395-PA, isoform A isof...	XP_967635.1	8,00E-33	85	69/81
66	CL61CONTIG1	REF|XP_535211.1| PREDICTED: similar to NADH dehydrogenase (ubiqu...	XP_535211.1	7,00E-19	54	42/77
67	CL62CONTIG1	EMB|CAG06843.1| unnamed protein product [Tetraodon nigroviridis]	CAG06843.1	3,00E-10	55	34/61
68	CL63CONTIG1	REF|XP_001500635.1| PREDICTED: similar to KIAA0098 protein [Equu...	XP_001500635.1	5,00E-08	93	30/32
69	CL64CONTIG1	EMB|CAD91421.1| ribosomal protein L5 [Crassostrea gigas]	CAD91421.1	4,00E-25	74	56/75
70	CL65CONTIG1	no blast match			0	
71	CL66CONTIG1	no blast match			0	
72	CL67CONTIG1	EMB|CAJ33888.1| putative serine protease inhibitor [Hydra vulgaris]	CAJ33888.1	1,00E-24	84	49/58
73	CL68CONTIG1	no blast match			0	

74	CL69CONTIG1	REF|XP_001363488.1| PREDICTED: similar to p34-Arc [Monodelphis d...	XP_001363488.1	2,00E-54	80	102/127
75	CL70CONTIG1	REF|XP_001521468.1| PREDICTED: hypothetical protein [Ornithorhyn...	XP_001521468.1	6,00E-13	41	28/67
76	CL71CONTIG1	no blast match			0	
77	CL72CONTIG1	GB|ABC25032.1| ribosomal protein L35A [Hydra vulgaris]	ABC25032.1	7,00E-50	97	97/100
78	CL73CONTIG1	EMB|CAI44701.1| selenoprotein M [Suberites domuncula]	CAI44701.1	2,00E-11	49	34/69
79	CL74CONTIG1	no blast match			0	
80	CL75CONTIG1	EMB|CAG04219.1| unnamed protein product [Tetraodon nigroviridis]	CAG04219.1	7,00E-13	65	36/55
81	CL76CONTIG1	EMB|CAA08791.1| ribosomal protein L18a [Podocoryne carnea]	CAA08791.1	3,00E-30	81	61/75
82	CL77CONTIG1	REF|XP_785610.1| PREDICTED: similar to ribosomal protein S10 iso...	XP_785610.1	3,00E-20	59	47/79
83	CL78CONTIG1	no blast match			0	
84	CL79CONTIG1	no blast match			0	
85	CL80CONTIG1	REF|XP_001483928.1| 60S ribosomal protein L37 [Pichia guilliermo...	XP_001483928.1	1,00E-24	81	49/60
86	CL81CONTIG1	REF|YP_502335.1| hypothetical protein Mhun_0865 [Methanospirillu...	YP_502335.1	1,00E-08	30	33/108
87	CL82CONTIG1	GB|EAZ41147.1| hypothetical protein OsJ_024630 [Oryza sativa (ja...	EAZ41147.1	4,00E-12	42	36/85
88	CL83CONTIG1	REF|XP_684064.1| PREDICTED: hypothetical protein [Danio rerio]	XP_684064.1	5,00E-08	49	28/57
89	CL84CONTIG1	GB|AAX09935.1| GTP-binding protein [Aurelia aurita]	AAX09935.1	2,00E-28	49	69/140
90	CL85CONTIG1	GB|AAW25342.1| SJCHGC02792 protein [Schistosoma japonicum]	AAW25342.1	2,00E-20	67	38/56
91	CL86CONTIG1	no blast match			0	
92	CL87CONTIG1	GB|AAN52389.1| ribosomal protein S17 [Branchiostoma belcheri]	AAN52389.1	3,00E-50	81	96/118
93	CL88CONTIG1	EMB|CAC19490.1| putative ribosomal protein L27 [Stichodactyla he...	CAC19490.1	8,00E-44	73	85/116
94	CL89CONTIG1	EMB|CAG12069.1| unnamed protein product [Tetraodon nigroviridis]	CAG12069.1	4,00E-33	87	68/78
95	CL90CONTIG1	no blast match			0	
96	CL91CONTIG1	REF|NP_001037135.1| ribosomal protein L7 [Bombyx mori] >gi|54609...	NP_001037135.1	1,00E-31	76	66/86
97	CL92CONTIG1	GB|AAN77896.1| ribosomal protein S7 [Petromyzon marinus]	AAN77896.1	4,00E-38	70	80/113
98	CL93CONTIG1	REF|XP_418537.1| PREDICTED: similar to xylulokinase homolog [Gal...	XP_418537.1	1,00E-32	45	78/172
99	CL94CONTIG1	no blast match			0	
100	CL95CONTIG1	no blast match			0	
101	CL96CONTIG1	no blast match			0	
102	CL97CONTIG1	EMB|CAF98037.1| unnamed protein product [Tetraodon nigroviridis]	CAF98037.1	6,00E-22	44	54/121
103	CL98CONTIG1	no blast match			0	
104	CL99CONTIG1	REF|XP_001518180.1| PREDICTED: similar to alpha NAC/1.9.2. prote...	XP_001518180.1	1,00E-35	68	80/116
105	CL100CONTIG1	EMB|CAN70636.1| hypothetical protein [Vitis vinifera]	CAN70636.1	1,00E-37	67	78/115
106	CL101CONTIG1	SP|Q27052|FCA1_TRYRA Flagellar calcium-binding protein >gi|11776...	Q27052	2,00E-07	38	27/70
107	CL102CONTIG1	no blast match			0	
108	CL103CONTIG1	REF|NP_001006472.1| CD151 antigen [Gallus gallus] >gi|53135766|e...	NP_001006472.1	9,00E-09	32	30/91
109	CL104CONTIG1	REF|XP_785807.2| PREDICTED: similar to apical endosomal glycopro...	XP_785807.2	6,00E-07	39	32/81
110	CL105CONTIG1	GB|AAR31143.1| zebra precursor; HyZebra [Hydra magnipapillata]	AAR31143.1	6,00E-25	46	53/114
111	CL106CONTIG1	REF|XP_001201373.1| PREDICTED: hypothetical protein [Strongyloce...	XP_001201373.1	8,00E-13	27	57/205
112	CL107CONTIG1	REF|XP_001196080.1| PREDICTED: similar to vesicle-associated pro...	XP_001196080.1	3,00E-07	36	38/104
113	CL108CONTIG1	REF|XP_780386.2| PREDICTED: hypothetical protein [Strongylocentr...	XP_780386.2	3,00E-09	35	42/118
114	CL109CONTIG1	no blast match			0	
115	CL110CONTIG1	REF|NP_001086867.1| MGC83668 protein [Xenopus laevis] >gi|504155...	NP_001086867.1	4,00E-24	40	57/141
116	CL111CONTIG1	SP|Q08699|RS14_PODCA 40S ribosomal protein S14 >gi|396252|emb|CA...	Q08699	1,00E-68	93	131/140
117	CL112CONTIG1	no blast match			0	
118	CL113CONTIG1	no blast match			0	
119	CL114CONTIG1	REF|NP_001026019.1| integrin beta 4 binding protein [Gallus gall...	NP_001026019.1	3,00E-71	84	129/152
120	CL115CONTIG1	GB|AAQ13347.1|U60651_1 ribosomal protein L10 [Hydra vulgaris]	AAQ13347.1	1,00E-37	98	73/74
121	CL116CONTIG1	GB|AAO86696.1| selenoprotein W1 [Danio rerio]	AAO86696.1	5,00E-19	54	46/84
122	CL117CONTIG1	no blast match			0	
123	CL118CONTIG1	no blast match			0	
124	CL119CONTIG1	no blast match			0	
125	CL120CONTIG1	no blast match			0	
126	CL121CONTIG1	GB|EAT45962.1| coupling factor, putative [Aedes aegypti]	EAT45962.1	5,00E-08	41	35/84
127	CL122CONTIG1	EMB|CAL36995.1| chitinase 2 [Hydractinia echinata]	CAL36995.1	2,00E-14	56	32/57
128	CL123CONTIG1	GB|AAX48842.1| L10a [Suberites domuncula]	AAX48842.1	2,00E-78	76	143/188
129	CL124CONTIG1	no blast match			0	
130	CL125CONTIG1	GB|ABC25025.1| extracellular copper/zinc superoxide dismutase [H...	ABC25025.1	9,00E-16	90	39/43
131	CL126CONTIG1	no blast match			0	
132	CL127CONTIG1	GB|AAA92361.2| metalloproteinase 1 [Hydra vulgaris]	AAA92361.2	1,00E-42	86	80/92
133	CL128CONTIG1	SP|P17126|ACT_HYDAT Actin, non-muscle 6.2 >gi|159254|gb|AAA29205...	P17126	1,00E-42	95	85/89
134	CL129CONTIG1	no blast match			0	
135	CL130CONTIG1	no blast match			0	
136	CL131CONTIG1	SP|Q7TNV0|DEK_MOUSE Protein DEK >gi|33416321|gb|AAH55451.1| DEK ...	Q7TNV0	4,00E-27	42	59/140
137	CL132CONTIG1	no blast match			0	
138	CL133CONTIG1	no blast match			0	
139	CL134CONTIG1	GB|ABM55524.1| vacuolar ATPase G subunit-like protein [Maconelli...	ABM55524.1	8,00E-14	48	40/83
140	CL135CONTIG1	no blast match			0	
141	CL137CONTIG1	no blast match			0	
142	CL138CONTIG1	REF|XP_788351.2| PREDICTED: similar to myb binding protein (P160...	XP_788351.2	4,00E-20	27	66/240
143	CL139CONTIG1	REF|XP_716284.1| putative ADP-ribosylation factor [Candida albic...	XP_716284.1	3,00E-46	52	60/114
144	CL140CONTIG1	GB|ABR19841.1| minicollagen-15 [Hydra vulgaris]	ABR19841.1	8,00E-41	83	72/86
145	CL141CONTIG1	REF|XP_001120773.1| PREDICTED: similar to CG17266-PA [Apis melli...	XP_001120773.1	4,00E-40	80	76/95
146	CL142CONTIG1	REF|XP_001191061.1| PREDICTED: hypothetical protein, partial [St...	XP_001191061.1	1,00E-06	31	26/82
147	CL144CONTIG1	GB|AAN52376.1| ribosomal protein L23a [Branchiostoma belcheri]	AAN52376.1	8,00E-46	74	92/123
148	CL145CONTIG1	no blast match			0	
149	CL146CONTIG1	no blast match			0	
150	CL147CONTIG1	DBJ|BAE31061.1| unnamed protein product [Mus musculus]	BAE31061.1	4,00E-14	35	39/111
151	CL148CONTIG1	no blast match			0	
152	CL149CONTIG1	no blast match			0	
153	CL150CONTIG1	no blast match			0	
154	CL151CONTIG1	REF|XP_001474742.1| PREDICTED: hypothetical protein [Mus musculu...	XP_001474742.1	1,00E-15	75	43/57
155	CL152CONTIG1	REF|NP_001091148.1| hypothetical protein LOC100036901 [Xenopus l...	NP_001091148.1	1,00E-11	32	45/139
156	CL153CONTIG1	GB|AAH45916.1| Zgc:77734 [Danio rerio]	AAH45916.1	2,00E-23	67	53/79
157	CL154CONTIG1	EMB|CAG01631.1| unnamed protein product [Tetraodon nigroviridis]	CAG01631.1	4,00E-27	75	65/86
158	CL155CONTIG1	no blast match			0	
159	CL156CONTIG1	REF|XP_623766.2| PREDICTED: similar to Hsp70/Hsp90 organizing pr...	XP_623766.2	5,00E-66	55	128/230
160	CL157CONTIG1	EMB|CAB88388.1| L12 ribosomal protein [Hydra vulgaris]	CAB88388.1	1,00E-56	97	72/74
161	CL158CONTIG1	GB|EDL81715.1| rCG20808, isoform CRA_b [Rattus norvegicus]	EDL81715.1	1,00E-25	100	59/59
162	CL159CONTIG1	no blast match			0	
163	CL160CONTIG1	REF|XP_001494750.1| PREDICTED: similar to translocation protein ...	XP_001494750.1	1,00E-29	45	72/160

164	CL161CONTIG1	GB|AAX08983.1| peptidylprolyl isomerase B precursor [Bos taurus]...	AAX08983.1	3,00E-63	64	123/192
165	CL162CONTIG1	no blast match			0	
166	CL163CONTIG1	GB|AAO43049.1| 40S ribosomal protein [Perinereis aibuhitensis]	AAO43049.1	3,00E-48	81	91/112
167	CL164CONTIG1	no blast match			0	
168	CL165CONTIG1	REF|NP_001040289.1| prohibitin protein WPH [Bombyx mori] >gi|872...	NP_001040289.1	7,00E-45	70	91/129
169	CL166CONTIG1	no blast match			0	
170	CL167CONTIG1	REF|NP_001079738.1| hypothetical protein LOC379427 [Xenopus laev...	NP_001079738.1	2,00E-07	51	28/54
171	CL168CONTIG1	no blast match			0	
172	CL169CONTIG1	no blast match			0	
173	CL170CONTIG1	no blast match			0	
174	CL171CONTIG1	no blast match			0	
175	CL172CONTIG1	no blast match			0	
176	CL173CONTIG1	no blast match			0	
177	CL174CONTIG1	no blast match			0	
178	CL175CONTIG1	GB|AAO11519.1| ribosomal protein S19 [Chlamys farreri]	AAO11519.1	1,00E-27	70	56/79
179	CL176CONTIG1	GB|AAR19205.1| type II transmembrane C-type lectin [Hydra magnip...	AAR19205.1	2,00E-41	92	75/81
180	CL177CONTIG1	REF|XP_780458.1| PREDICTED: similar to Polymerase (RNA) II (DNA ...	XP_780458.1	2,00E-47	88	83/94
181	CL178CONTIG1	no blast match			0	
182	CL179CONTIG1	no blast match			0	
183	CL180CONTIG1	REF|XP_001377662.1| PREDICTED: hypothetical protein [Monodelphis...	XP_001377662.1	5,00E-08	34	33/95
184	CL182CONTIG1	no blast match			0	
185	CL183CONTIG1	no blast match			0	
186	CL184CONTIG1	no blast match			0	
187	CL185CONTIG1	GB|ABN11959.1| putative chaperonin subunit 6a zeta [Maconellicoc...	ABN11959.1	1,00E-14	75	37/49
188	CL187CONTIG1	no blast match			0	
189	CL188CONTIG1	GB|AAX09924.1| DnaJ-like protein [Aurelia aurita]	AAX09924.1	1,00E-22	72	50/69
190	CL189CONTIG1	no blast match			0	
191	CL190CONTIG1	no blast match			0	
192	CL191CONTIG1	no blast match			0	
193	CL192CONTIG1	REF|XP_312008.3| ENSANGP00000018753 [Anopheles gambiae str. PEST...	XP_312008.3	7,00E-26	61	62/101
194	CL193CONTIG1	no blast match			0	
195	CL194CONTIG1	SP|Q94587|TCTP_HYDAT Translationally-controlled tumor protein ho...	Q94587	1,00E-48	93	91/97
196	CL196CONTIG1	no blast match			0	
197	CL197CONTIG1	no blast match			0	
198	CL198CONTIG1	REF|XP_453843.1| unnamed protein product [Kluyveromyces lactis] ...	XP_453843.1	1,00E-11	55	37/67
199	CL199CONTIG1	REF|XP_001493068.1| PREDICTED: similar to elongation factor SIII...	XP_001493068.1	1,00E-16	78	41/52
200	CL200CONTIG1	REF|NP_001017583.1| hypothetical protein LOC550245 [Danio rerio]...	NP_001017583.1	4,00E-28	54	59/109
201	CL201CONTIG1	no blast match			0	
202	CL202CONTIG1	no blast match			0	
203	CL203CONTIG1	no blast match			0	
204	CL204CONTIG1	REF|NP_001080153.1| actin related protein 2/3 complex, subunit 1...	NP_001080153.1	3,00E-28	61	50/81
205	CL206CONTIG1	no blast match			0	
206	CL207CONTIG1	no blast match			0	
207	CL208CONTIG1	REF|XP_792994.1| PREDICTED: similar to muscle protein 20-like pr...	XP_792994.1	2,00E-18	83	44/53
208	CL209CONTIG1	SP|P38984|RSSA_CHLVR 40S ribosomal protein SA (p40) (33 kDa lami...	P38984	8,00E-52	81	94/115
209	CL210CONTIG1	no blast match			0	
210	CL211CONTIG1	REF|YP_563466.1| hypothetical protein Sden_2463 [Shewanella deni...	YP_563466.1	4,00E-10	43	43/98
211	CL212CONTIG1	GB|AAT97079.1| translocon associated protein gamma subunit-like ...	AAT97079.1	1,00E-14	59	35/59
212	CL213CONTIG1	no blast match			0	
213	CL214CONTIG1	no blast match			0	
214	CL215CONTIG1	REF|XP_697767.2| PREDICTED: similar to procathepsin B [Danio rerio]	XP_697767.2	9,00E-17	81	30/37
215	CL216CONTIG1	REF|NP_001006703.1| four and a half LIM domains 1 [Xenopus tropi...	NP_001006703.1	2,00E-11	43	30/69
216	CL217CONTIG1	no blast match			0	
217	CL218CONTIG1	REF|NP_001039500.1| hypothetical protein LOC509522 [Bos taurus] ...	NP_001039500.1	4,00E-12	41	33/80
218	CL219CONTIG1	EMB|CAJ33888.1| putative serine protease inhibitor [Hydra vulgaris]	CAJ33888.1	2,00E-40	92	75/81
219	CL220CONTIG1	DBJ|BAB22235.1| unnamed protein product [Mus musculus]	BAB22235.1	4,00E-21	61	49/80
220	CL221CONTIG1	REF|XP_318019.3| ENSANGP00000010754 [Anopheles gambiae str. PEST...	XP_318019.3	7,00E-54	71	95/133
221	CL222CONTIG1	no blast match			0	
222	CL223CONTIG1	DBJ|BAC92690.1| epitheliopeptide HYM-301 [Hydra magnipapillata]	BAC92690.1	3,00E-13	54	43/79
223	CL224CONTIG1	REF|XP_783992.2| PREDICTED: similar to sorcin [Strongylocentrotu...	XP_783992.2	6,00E-42	71	87/122
224	CL225CONTIG1	GB|AAA29218.2| tyrosine kinase receptor [Hydra vulgaris]	AAA29218.2	3,00E-07	32	34/105
225	CL226CONTIG1	EMB|CAJ65510.3| HyTSR1 protein [Hydra vulgaris]	CAJ65510.3	2,00E-06	34	33/95
226	CL227CONTIG1	GB|AAX09926.1| putative tyrosine-rich heat shock protein [Aureli...	AAX09926.1	3,00E-17	70	41/58
227	CL228CONTIG1	GB|AAX48841.1| L10e/P0 [Suberites domuncula]	AAX48841.1	4,00E-22	69	50/72
228	CL229CONTIG1	no blast match			0	
229	CL230CONTIG1	no blast match			0	
230	CL231CONTIG1	no blast match			0	
231	CL232CONTIG1	EMB|CAA15924.1| SPAC3G9.15c [Schizosaccharomyces pombe]	CAA15924.1	4,00E-08	52	29/55
232	CL233CONTIG1	GB|ABF18043.1| 60S ribosomal protein L38 [Aedes aegypti] >gi|108...	ABF18043.1	2,00E-17	74	44/59
233	CL234CONTIG1	REF|XP_001338556.1| PREDICTED: hypothetical protein [Danio rerio]	XP_001338556.1	1,00E-13	40	46/115
234	CL235CONTIG1	REF|XP_310323.4| ENSANGP00000015846 [Anopheles gambiae str. PEST...	XP_310323.4	1,00E-49	97	92/94
235	CL236CONTIG1	EMB|CAM25755.1| ribosomal protein L11 [Mus musculus]	CAM25755.1	5,00E-38	81	76/93
236	CL237CONTIG1	no blast match			0	
237	CL238CONTIG1	GB|AAM18885.1|AF391293_2 unknown [Branchiostoma floridae]	AAM18885.1	3,00E-10	80	29/36
238	CL239CONTIG1	GB|AAT00507.1| Kazal-like serine protease inhibitor EPI8 [Phytop...	AAT00507.1	2,00E-08	39	27/68
239	CL240CONTIG1	no blast match			0	
240	CL241CONTIG1	no blast match			0	
241	CV284315.1	REF|NP_001089378.1| hypothetical protein LOC734428 [Xenopus laev...	NP_001089378.1	7,00E-32	60	38/63
242	CV284316.1	REF|NP_001006694.1| malate dehydrogenase 1, NAD (soluble) [Xenop...	NP_001006694.1	2,00E-33	62	72/115
243	CV284320.1	REF|NP_610932.1| Transport and Golgi organization 7 CG8309-PA [D...	NP_610932.1	2,00E-11	38	32/83
244	CV284323.1	REF|XP_001380175.1| PREDICTED: similar to KIAA1611 protein [Mono...	XP_001380175.1	2,00E-25	41	56/135
245	CV284324.1	REF|XP_001366707.1| PREDICTED: hypothetical protein [Monodelphis...	XP_001366707.1	7,00E-24	85	55/64
246	CV284326.1	REF|NP_508154.2| Nematode AStacin protease family member (nas-15...	NP_508154.2	5,00E-06	41	31/75
247	CV284328.1	no blast match			0	
248	CV284329.1	REF|XP_975859.1| PREDICTED: similar to 60S acidic ribosomal prot...	XP_975859.1	6,00E-12	75	40/53
249	CV284335.1	no blast match			0	
250	CV284337.1	DBJ|BAA74509.1| actin-binding protein ABP-280 [Hydra magnipapill...	BAA74509.1	1,00E-11	39	35/88
251	CV284342.1	no blast match			0	
252	CV284344.1	no blast match			0	
253	CV284347.1	REF|NP_001080221.1| coatomer protein complex, subunit beta 2 (be...	NP_001080221.1	4,00E-13	41	44/107

254	CV284349.1	REF|XP_971367.1| PREDICTED: similar to CG15786-PA [Tribolium cas...	XP_971367.1	3,00E-31	45	72/159
255	CV284352.1	no blast match			0	
256	CV284353.1	REF|XP_001331697.1| PREDICTED: hypothetical protein [Danio rerio]	XP_001331697.1	2,00E-10	76	29/38
257	CV284356.1	SP|P37380|RL17_PODCA 60S ribosomal protein L17 (L23) >gi|312500|...	P37380	3,00E-48	84	95/112
258	CV284358.1	GB|AAX62427.1| ribosomal protein L8 [Lysiphlebus testaceipes]	AAX62427.1	1,00E-28	76	62/81
259	CV284359.1	no blast match			0	
260	CV284360.1	REF|XP_970528.1| PREDICTED: similar to CG14210-PA [Tribolium cas...	XP_970528.1	1,00E-09	48	36/74
261	CV284362.1	REF|XP_797203.2| PREDICTED: similar to rhamnose-binding lectin (...	XP_797203.2	2,00E-24	45	60/132
262	CV284370.1	no blast match			0	
263	CV284381.1	REF|XP_001375174.1| PREDICTED: similar to THUMP domain containin...	XP_001375174.1	9,00E-45	45	89/194
264	CV284385.1	no blast match			0	
265	CV284393.1	REF|XP_001507485.1| PREDICTED: similar to mitochondrial complex ...	XP_001507485.1	9,00E-19	81	45/55
266	CV284394.1	REF|XP_536910.2| PREDICTED: similar to major vault protein [Cani...	XP_536910.2	3,00E-23	63	56/88
267	CV284396.1	REF|XP_001265199.1| 60S ribosomal protein L15 [Neosartorya fisch...	XP_001265199.1	8,00E-12	81	31/38
268	CV284397.1	no blast match			0	
269	CV284404.1	SP|Q4PM47|RS29_IXOSC 40S ribosomal protein S29 >gi|67083965|gb|A...	Q4PM47	2,00E-20	75	41/54
270	CV284408.1	GB|EAT45815.1| conserved hypothetical protein [Aedes aegypti]	EAT45815.1	4,00E-13	57	35/61
271	CV284409.1	REF|XP_781777.1| PREDICTED: similar to Ribosomal protein S2 [Str...	XP_781777.1	6,00E-08	49	38/77
272	CV284413.1	no blast match			0	
273	CV284414.1	REF|NP_001026431.1| uridine monophosphate synthase [Gallus gallu...	NP_001026431.1	1,00E-27	50	59/116
274	CV284417.1	GB|AAW27025.1| SJCHGC02793 protein [Schistosoma japonicum]	AAW27025.1	3,00E-18	78	44/56
275	CV284419.1	no blast match			0	
276	CV284425.1	no blast match			0	
277	CV284427.1	REF|NP_001080733.1| nuclear RNA helicase [Xenopus laevis] >gi|28...	NP_001080733.1	1,00E-13	82	38/46
278	CV284430.1	no blast match			0	
279	CV284438.1	EMB|CAJ33888.1| putative serine protease inhibitor [Hydra vulgaris]	CAJ33888.1	2,00E-46	91	82/90
280	CV284439.1	no blast match			0	
281	CV284453.1	GB|AAX48879.1| S3a [Suberites domuncula]	AAX48879.1	2,00E-65	70	119/168
282	CV284455.1	GB|EAU84863.1| hypothetical protein CC1G_00382 [Coprinopsis cine...	EAU84863.1	4,00E-25	67	59/87
283	CV284458.1	SP|Q33845|COX3_ARTSA Cytochrome c oxidase subunit 3 (Cytochrome ...	Q33845	3,00E-12	89	34/38
284	CV284464.1	GB|AAP88387.1| actin [Chlamys farreri]	AAP88387.1	4,00E-72	98	136/138
285	CV284470.1	GB|ABD04179.1| 60S ribosomal protein L26-like protein [Anthopleu...	ABD04179.1	2,00E-23	74	52/70
286	CV284474.1	GB|AAA29918.1| alpha tubulin >gi|56758676|gb|AAW27478.1| SJCHGC0...	AAA29918.1	1,00E-20	92	48/52
287	CV284480.1	SP|Q4VUZ1|RHCG_ONCMY Ammonium transporter Rh type C (Rh family t...	Q4VUZ1	7,00E-11	41	41/100
288	CV284487.1	GB|EAT40263.1| SEC63 protein, putative [Aedes aegypti]	EAT40263.1	7,00E-37	64	76/118
289	CV284488.1	GB|AAW26562.1| SJCHGC09076 protein [Schistosoma japonicum]	AAW26562.1	3,00E-12	58	39/67
290	CV284495.1	REF|NP_511178.1| enoyl Coenzyme A hydratase, short chain, 1, mit...	NP_511178.1	3,00E-19	52	49/94
291	CV284496.1	GB|EAX07023.1| ribosomal protein S8, isoform CRA_a [Homo sapiens]	EAX07023.1	4,00E-33	73	65/89
292	CV284502.1	no blast match			0	
293	CV284508.1	REF|XP_001508639.1| PREDICTED: hypothetical protein [Ornithorhyn...	XP_001508639.1	5,00E-24	50	57/113
294	CV284512.1	no blast match			0	
295	CV284521.1	REF|XP_001199603.1| PREDICTED: similar to Expressed sequence AI4...	XP_001199603.1	2,00E-13	60	33/55
296	CV284523.1	no blast match			0	
297	CV284532.1	GB|EAT36156.1| glutathione s-transferase [Aedes aegypti]	EAT36156.1	1,00E-07	46	30/65
298	CV284537.1	GB|EAY87310.1| hypothetical protein OsI_008543 [Oryza sativa (in...	EAY87310.1	1,00E-08	63	30/47
299	CV284539.1	no blast match			0	
300	CV284543.1	REF|XP_001012860.1| hypothetical protein TTHERM_00094060 [Tetrah...	XP_001012860.1	2,00E-27	57	52/90
301	CV284548.1	EMB|CAE67096.1| Hypothetical protein CBG12507 [Caenorhabditis br...	CAE67096.1	6,00E-17	40	52/128
302	CV284552.1	EMB|CAG04119.1| unnamed protein product [Tetraodon nigroviridis]	CAG04119.1	2,00E-22	51	54/105
303	CV284553.1	GB|EAT39816.1| glutathione peroxidase [Aedes aegypti] >gi|108875...	EAT39816.1	1,00E-09	44	30/67
304	CV284555.1	GB|AAY19458.1| secreted protein [Ciona intestinalis]	AAY19458.1	6,00E-06	28	33/117
305	CV284566.1	no blast match			0	
306	CV284569.1	no blast match			0	
307	CV284585.1	SP|P38984|RSSA_CHLVR 40S ribosomal protein SA (p40) (33 kDa lami...	P38984	2,00E-70	86	116/134
308	CV284597.1	GB|AAW26562.1| SJCHGC09076 protein [Schistosoma japonicum]	AAW26562.1	2,00E-12	59	38/64
309	CV284599.1	REF|NP_198938.1| ATGSTT3 (Arabidopsis thaliana Glutathione S-tra...	NP_198938.1	7,00E-06	34	32/92
310	CV284600.1	no blast match			0	
311	CV284607.1	GB|AAO65603.1| cathepsin L precursor [Hydra vulgaris]	AAO65603.1	2,00E-28	77	57/74
312	CV284633.1	no blast match			0	
313	CV284636.1	no blast match			0	
314	CV284638.1	EMB|CAJ65510.3| HyTSR1 protein [Hydra vulgaris]	CAJ65510.3	3,00E-17	37	49/132
315	CV284639.1	no blast match			0	
316	CV285050.1	EMB|CAA08792.1| ribosomal protein L9 [Podocoryne carnea]	CAA08792.1	1,00E-42	78	84/107
317	CV285054.1	REF|XP_001176123.1| PREDICTED: similar to gelsolin [Strongylocen...	XP_001176123.1	7,00E-37	74	77/103
318	CV285057.1	no blast match			0	
319	CV285062.1	no blast match			0	
320	CV285064.1	REF|NP_001037524.1| ribosomal protein S10 [Bombyx mori] >gi|5460...	NP_001037524.1	2,00E-14	73	39/53
321	CV285066.1	REF|NP_001080713.1| chaperonin containing TCP1, subunit 8 (theta...	NP_001080713.1	6,00E-57	70	104/147
322	CV285067.1	no blast match			0	
323	CV285070.1	no blast match			0	
324	CV285078.1	GB|AAX09934.1| ganglioside M2 activator-like protein [Aurelia au...	AAX09934.1	3,00E-14	37	49/130
325	CV285082.1	REF|XP_788935.2| PREDICTED: similar to Ribosomal protein L14, pa...	XP_788935.2	3,00E-14	50	37/73
326	CV285084.1	GB|AAL55470.1| ubiquitin/ribosomal protein S27a fusion protein [...	AAL55470.1	6,00E-25	77	51/66
327	CV285087.1	no blast match			0	
328	CV285089.1	GB|AAW27674.1| SJCHGC04249 protein [Schistosoma japonicum]	AAW27674.1	2,00E-28	79	57/72
329	CV285092.1	no blast match			0	
330	CV285094.1	no blast match			0	
331	CV285099.1	no blast match			0	
332	CV285106.1	no blast match			0	
333	CV285108.1	EMB|CAN76945.1| hypothetical protein [Vitis vinifera]	CAN76945.1	5,00E-21	34	46/134
334	CV285111.1	REF|XP_001502076.1| PREDICTED: similar to SWI/SNF-related matrix...	XP_001502076.1	5,00E-20	68	50/73
335	CV285117.1	GB|ABF18291.1| multifunctional 14-3-3 family chaperone [Aedes ae...	ABF18291.1	5,00E-17	70	44/62
336	CV285119.1	GB|AAX48887.1| S11 [Suberites domuncula]	AAX48887.1	9,00E-21	90	48/53
337	CV285126.1	REF|XP_001311347.1| hypothetical protein TVAG_264660 [Trichomona...	XP_001311347.1	3,00E-09	33	40/118
338	CV285128.1	no blast match			0	
339	CV285129.1	no blast match			0	
340	CV285131.1	GB|EAW70379.1| NADH dehydrogenase (ubiquinone) Fe-S protein 1, 7...	EAW70379.1	7,00E-37	68	75/109
341	CV285137.1	GB|EAU85127.1| hypothetical protein CC1G_08100 [Coprinopsis cine...	EAU85127.1	3,00E-32	52	71/136
342	CV285138.1	REF|XP_786540.2| PREDICTED: similar to astacin-like protein [Str...	XP_786540.2	3,00E-07	31	35/110
343	CV285146.1	REF|XP_001504089.1| PREDICTED: hypothetical protein [Equus cabal...	XP_001504089.1	3,00E-06	65	19/29

344	CV285147.1	no blast match			0	
345	CV285148.1	no blast match			0	
346	CV285152.1	REF|NP_001077233.1| hypothetical protein LOC614055 [Bos taurus] ...	NP_001077233.1	1,00E-20	78	50/64
347	CV285157.1	GB|EAU84784.1| hypothetical protein CC1G_00303 [Coprinopsis cine...	EAU84784.1	3,00E-11	54	32/59
348	CV285163.1	no blast match			0	
349	CV285165.1	no blast match			0	
350	CV285173.1	GB|AAO65603.1| cathepsin L precursor [Hydra vulgaris]	AAO65603.1	7,00E-19	85	46/54
351	CV285177.1	REF|XP_001135046.1| PREDICTED: similar to RNA binding protein El...	XP_001135046.1	5,00E-22	41	68/162
352	CV285179.1	REF|XP_417405.2| PREDICTED: similar to Rps21-prov protein [Gallu...	XP_417405.2	4,00E-19	54	45/83
353	CV285182.1	no blast match			0	
354	CV285186.1	no blast match			0	
355	CV285187.1	no blast match			0	
356	CV285193.1	no blast match			0	
357	CV285197.1	REF|XP_001370789.1| PREDICTED: similar to KIAA0947 protein [Mono...	XP_001370789.1	3,00E-06	26	33/126
358	CV285198.1	no blast match			0	
359	CV285199.1	no blast match			0	
360	CV285200.1	no blast match			0	
361	CV285202.1	REF|NP_868383.1| hypothetical protein-signal peptide and transme...	NP_868383.1	5,00E-15	48	38/79
362	CV285213.1	no blast match			0	
363	CV285216.1	no blast match			0	
364	CV285222.1	GB|AAO15464.1|AF441167_1 60S ribosomal protein L15 [Spodoptera f...	AAO15464.1	1,00E-31	68	64/94
365	CV285225.1	GB|ABG74924.1| calmodulin [Aegiceras corniculatum]	ABG74924.1	3,00E-29	42	60/142
366	CV285229.1	no blast match			0	
367	CV285237.1	no blast match			0	
368	CV285239.1	REF|XP_001234246.1| PREDICTED: similar to GekBS036P [Gallus gallus]	XP_001234246.1	1,00E-15	53	41/76
369	CV285240.1	REF|XP_001342200.1| PREDICTED: hypothetical protein [Danio rerio]	XP_001342200.1	7,00E-06	32	29/88
370	CV285242.1	no blast match			0	
371	CV285244.1	GB|ABK32077.1| ribosomal protein S21 [Branchiostoma belcheri]	ABK32077.1	7,00E-14	71	38/53
372	CV285249.1	no blast match			0	
373	CV285251.1	no blast match			0	
374	CV285253.1	no blast match			0	
375	CV285254.1	EMB|CAD27733.1| S6 ribosomal protein [Paracentrotus lividus]	CAD27733.1	9,00E-11	62	34/54
376	CV285255.1	no blast match			0	
377	CV285256.1	REF|NP_032329.1| heat shock protein 1 (chaperonin 10) [Mus muscu...	NP_032329.1	4,00E-20	67	52/77
378	CV285257.1	GB|EDL19006.1| actin related protein 2/3 complex, subunit 1A, is...	EDL19006.1	9,00E-32	59	62/104
379	CV285264.1	no blast match			0	
380	CV285266.1	REF|NP_001087319.1| MGC85413 protein [Xenopus laevis] >gi|515931...	NP_001087319.1	1,00E-17	69	39/56
381	CV285268.1	REF|NP_001034418.1| peroxiredoxin 6 [Gallus gallus] >gi|82233783...	NP_001034418.1	3,00E-35	75	57/76
382	CV285270.1	EMB|CAK04297.1| novel protein similar to mouse and human exosome...	CAK04297.1	1,00E-11	34	31/91
383	CV285309.1	GB|AAX09929.1| glutathione peroxidase [Aurelia aurita]	AAX09929.1	6,00E-31	70	58/82
384	CV285313.1	DBJ|BAA85005.1| Hym-355 preprohormone [Hydra magnipapillata]	BAA85005.1	1,00E-39	89	81/91
385	CV285344.1	REF|XP_001340803.1| PREDICTED: similar to 19.9kD myosin light ch...	XP_001340803.1	7,00E-18	52	48/92
386	CV285348.1	no blast match			0	
387	CV285354.1	no blast match			0	
388	CV285358.1	no blast match			0	
389	CV285370.1	REF|XP_001496243.1| PREDICTED: similar to lacrimal androgen-bind...	XP_001496243.1	3,00E-16	64	38/59
390	CV285371.1	PIR||A53221 acidic ribosomal protein P1 - hydromedusa (Polyorchi...		3,00E-10	74	29/39
391	CV285375.1	no blast match			0	
392	CV285378.1	GB|AAN73353.1| ribosomal protein L19 [Petromyzon marinus]	AAN73353.1	6,00E-20	72	42/58
393	CV285385.1	no blast match			0	
394	CV285401.1	REF|XP_970535.1| PREDICTED: similar to CG17636-PA.3 [Tribolium c...	XP_970535.1	1,00E-06	36	35/96
395	CV285404.1	no blast match			0	
396	CV285411.1	no blast match			0	
397	CV285416.1	REF|XP_749492.1| 40S ribosomal protein S21 [Aspergillus fumigatu...	XP_749492.1	3,00E-20	60	44/73
398	CV285419.1	GB|ABG81375.1| actin [Metopus es]	ABG81375.1	6,00E-28	86	64/74
399	CV285420.1	REF|XP_422279.1| PREDICTED: hypothetical protein [Gallus gallus]	XP_422279.1	4,00E-11	50	33/66
400	CV285426.1	no blast match			0	
401	CV285435.1	REF|XP_308336.3| ENSANGP00000010951 [Anopheles gambiae str. PEST...	XP_308336.3	1,00E-36	83	71/85
402	CV285442.1	no blast match			0	
403	CV285448.1	no blast match			0	
404	CV285449.1	no blast match			0	
405	CV285451.1	no blast match			0	
406	CV285458.1	no blast match			0	
407	CV285462.1	GB|ABI52727.1| ADP ribosylation factor 79F [Argas monolakensis]	ABI52727.1	1,00E-27	87	56/64
408	CV285463.1	no blast match			0	
409	CV285464.1	no blast match			0	
410	CV285466.1	EMB|CAE70384.1| Hypothetical protein CBG16945 [Caenorhabditis br...	CAE70384.1	2,00E-79	76	138/180
411	CV285468.1	REF|XP_362626.2| predicted protein [Magnaporthe grisea 70-15] >g...	XP_362626.2	2,00E-09	36	47/128
412	CV285472.1	GB|AAZ99726.1| dickkopf-like protein Dlp-2 precursor [Hydra magn...	AAZ99726.1	5,00E-27	75	53/70
413	CV285485.1	REF|XP_001362150.1| PREDICTED: similar to HECT, UBA and WWE doma...	XP_001362150.1	6,00E-26	62	59/94
414	CV285486.1	GB|ABC25033.1| ribosomal protein L35 [Hydra vulgaris]	ABC25033.1	1,00E-48	96	99/103
415	CV285492.1	no blast match			0	
416	CV285502.1	no blast match			0	
417	CV285507.1	EMB|CAF99476.1| unnamed protein product [Tetraodon nigroviridis]	CAF99476.1	3,00E-15	42	43/101
418	CV285521.1	no blast match			0	
419	CV285522.1	DBJ|BAB47390.1| mitochondrial ATP synthase gamma-subunit [Cyprin...	BAB47390.1	1,00E-13	58	36/62
420	CV285523.1	GB|AAR39410.1| beta tubulin [Chlamys farreri]	AAR39410.1	3,00E-34	92	70/76
421	CV285525.1	GB|ABM55583.1| putative 60S ribosomal protein L23 [Maconellicocc...	ABM55583.1	3,00E-19	95	45/47
422	CV285528.1	no blast match			0	
423	CV285529.1	no blast match			0	
424	CV285537.1	no blast match			0	
425	CV285546.1	GB|AAX48838.1| L7a [Suberites domuncula]	AAX48838.1	2,00E-20	70	50/71
426	CV285551.1	no blast match			0	
427	CV285581.1	no blast match			0	
428	CV285594.1	REF|XP_782614.2| PREDICTED: similar to Ribophorin I [Strongyloce...	XP_782614.2	4,00E-41	46	85/182
429	CV285597.1	GB|AAQ96733.1| bzip transcription factor C/EBP [Podocoryne carnea]	AAQ96733.1	3,00E-35	63	77/122
430	CV285601.1	no blast match			0	
431	CV285621.1	EMB|CAN88468.1| novel protein similar to vertebrate eukaryotic t...	CAN88468.1	6,00E-44	71	93/130
432	CV285628.1	REF|XP_001334181.1| PREDICTED: hypothetical protein [Danio rerio]	XP_001334181.1	6,00E-36	43	77/176
433	CV285629.1	EMB|CAG04053.1| unnamed protein product [Tetraodon nigroviridis]	CAG04053.1	2,00E-17	28	63/221

434	CV285632.1	no blast match			0	
435	CV285636.1	no blast match			0	
436	CV285637.1	no blast match			0	
437	CV285642.1	REF|XP_515260.2| PREDICTED: similar to peroxisomal integral memb...	XP_515260.2	8,00E-39	46	89/190
438	CV285644.1	REF|XP_977138.2| hypothetical protein TTHERM_00037370 [Tetrahyme...	XP_977138.2	2,00E-09	46	28/60
439	CV285653.1	REF|XP_791613.2| PREDICTED: similar to PH-4 protein, partial [St...	XP_791613.2	2,00E-31	46	65/140
440	CV285654.1	no blast match			0	
441	CV285673.1	no blast match			0	
442	CV285684.1	SP|P41114|TPM1_PODCA Tropomyosin-1 >gi|397472|emb|CAA50550.1| Tr...	P41114	3,00E-28	53	73/136
443	CV285701.1	GB|ABF21076.1| theromacin [Aplysia californica]	ABF21076.1	6,00E-12	50	29/57
444	CV285707.1	DBJ|BAF63639.1| calreticulin [Crassostrea gigas]	BAF63639.1	2,00E-54	68	94/137
445	CV285713.1	REF|XP_001120827.1| PREDICTED: similar to Karyopherin 3 CG1059-...	XP_001120827.1	4,00E-28	55	54/97
446	CV285714.1	no blast match			0	
447	CV285722.1	REF|XP_001177508.1| PREDICTED: similar to conserved hypothetical...	XP_001177508.1	5,00E-20	59	47/79
