# Supplementary material for: A Novel Gene Family Controls Species-Specific Morphological Traits in Hydra
Source: PLoS Biol. 2008 Nov 18;6(11):e278. doi: 10.1371/journal.pbio.0060278 (PMC2586386; doi:10.1371/journal.pbio.0060278)
Supplement: Table S2 — General information about clusters and singletons of H. magnipapillata-specific cDNA library (Kiel 7). Results of the BLASTX search against the nonredundant NCBI database. Sequences with E value ≥ 1e–5 were referred to as having no significant similarity to the proteins in NCBI database (potential TRGs). The clusters were numbered according to the amount of ESTs comprising them, with cluster 01 (CL01CONTIG1) being the largest. The consensus cluster sequences and singleton sequences are stored as a multi-sequence file (FASTA format) at the COMPAGEN server (http://compagen.zoologie.uni-kiel.de/retrieve.htm). (80 KB PDF) [file pbio.0060278.st002.pdf]

Table S2
KIEL 7 (*Hydra magnipapillata* specific) SSH library. Results of BLASTX search against non-redundant NCBI database

General information about clusters and singletons of *H. magnipapillata* -specific cDNA library (Kiel 7).
Results of the BLASTX search against non-redundant NCBI database. Sequences with E value ≥ 1e-5 were
referred to as having no significant homology to the proteins in NCBI database (potential taxon restricted genes, TGRs).
The clusters were numbered according to the amount of ESTs comprising them, with cluster 01 (CL01CONTIG1) being the largest.
The consensus cluster sequences and singleton sequences are stored as multi-sequence file (FASTA format) at the
COMPAGEN server (http://compagen.zoologie.uni-kiel.de/retrieve.htm).

Pct Idnt, % identity between *H.magnipapillata* amino acid sequence and the best BLASTX match from NCBI
Length (bp), length of consensus cluster sequence or singleton in base pares

N	Name	Best blast match (with E-value < 1e-5)	Accession number	Expect	Pct Idnt	Ratio Idnt	Length (bp)
1	CL1CONTIG1	DBJ|BAB33421.1| putative senescence-associated protein [Pisum sa...	BAB33421.1	2,00E-09	75	31/41	299
2	CL2CONTIG1	GB|ABC25029.1| ferritin [Hydra vulgaris]	ABC25029.1	3,00E-45	96	87/90	347
3	CL2CONTIG2	GB|ABC25029.1| ferritin [Hydra vulgaris]	ABC25029.1	8,00E-25	91	53/58	233
4	CL3CONTIG1	no blast match			0		581
5	CL3CONTIG2	no blast match			0		397
6	CL4CONTIG1	GB|ABC25029.1| ferritin [Hydra vulgaris]	ABC25029.1	4,00E-38	100	80/80	348
7	CL5CONTIG1	REF|XP_001120521.1| PREDICTED: similar to Ribosomal protein L40 ...	XP_001120521.1	3,00E-65	95	122/128	628
8	CL6CONTIG1	EMB|CAA08792.1| ribosomal protein L9 [Podocoryne carnea]	CAA08792.1	9,00E-30	79	62/78	240
9	CL7CONTIG1	no blast match			0		278
10	CL8CONTIG1	GB|ABC25036.1| ribosomal protein S9 [Hydra vulgaris]	ABC25036.1	5,00E-33	98	72/73	222
11	CL9CONTIG1	GB|ABR27976.1| ribosomal protein L11 [Triatoma infestans]	ABR27976.1	3,00E-79	79	140/176	575
12	CL10CONTIG1	EMB|CAD91420.1| ribosomal protein S3a [Crassostrea gigas]	CAD91420.1	1,00E-77	69	139/199	627
13	CL11CONTIG1	GB|AAN05584.1| ribosomal protein L30 [Argopecten irradians]	AAN05584.1	1,00E-30	81	67/82	279
14	CL12CONTIG1	no blast match			0		139
15	CL13CONTIG1	GB|EAT34547.1| 60S ribosomal protein L10a [Aedes aegypti] >gi|10...	EAT34547.1	2,00E-48	78	94/119	357
16	CL14CONTIG1	REF|XP_788935.2| PREDICTED: similar to Ribosomal protein L14, pa...	XP_788935.2	7,00E-13	49	35/71	283
17	CL15CONTIG1	GB|AAN52376.1| ribosomal protein L23a [Branchiostoma belcheri]	AAN52376.1	1,00E-37	83	77/92	280
18	CL16CONTIG1	EMB|CAN70636.1| hypothetical protein [Vitis vinifera]	CAN70636.1	3,00E-33	67	69/102	432
19	CL17CONTIG1	no blast match			0		466
20	CL18CONTIG1	no blast match			0		315
21	CL19CONTIG1	EMB|CAJ57448.1| astacin 2 [Hydractinia echinata]	CAJ57448.1	1,00E-07	46	29/63	203
22	CL20CONTIG1	no blast match			0		406
23	CL21CONTIG1	GB|ABC25033.1| ribosomal protein L35 [Hydra vulgaris]	ABC25033.1	3,00E-26	98	63/64	279
24	CL22CONTIG1	GB|AAZ99726.1| dickkopf-like protein Dlp-2 precursor [Hydra magn...	AAZ99726.1	3,00E-36	100	71/71	215
25	CL23CONTIG1	SP|P38984|RSSA_CHLVR 40S ribosomal protein SA (p40) (33 kDa lami...	P38984	2,00E-41	85	76/89	268
26	CL24CONTIG1	EMB|CAH92706.1| hypothetical protein [Pongo pygmaeus]	CAH92706.1	8,00E-07	44	29/65	327
27	CL25CONTIG1	GB|ABC25029.1| ferritin [Hydra vulgaris]	ABC25029.1	8,00E-17	86	40/46	337
28	CL26CONTIG1	GB|ABC25036.1| ribosomal protein S9 [Hydra vulgaris]	ABC25036.1	1,00E-11	100	32/32	178
29	CL27CONTIG1	no blast match			0		343
30	CL28CONTIG1	SP|P40122|CAP_CHLVR Adenylyl cyclase-associated protein (CAP) >g...	P40122	1,00E-15	79	46/58	214
31	CL29CONTIG1	no blast match			0		149
32	CL30CONTIG1	REF|XP_570211.1| WD-repeat protein [Cryptococcus neoformans var....	XP_570211.1	3,00E-10	44	30/68	413
33	CL31CONTIG1	no blast match			0		262
34	CL32CONTIG1	SP|Q94587|TCTP_HYDAT Translationally-controlled tumor protein ho...	Q94587	3,00E-27	98	59/60	314
35	CL32CONTIG2	SP|Q94587|TCTP_HYDAT Translationally-controlled tumor protein ho...	Q94587	5,00E-10	93	28/30	125
36	CL33CONTIG1	DBJ|BAA82365.1| chymotrypsinogen 1 [Paralichthys olivaceus]	BAA82365.1	5,00E-12	50	35/70	433
37	CL34CONTIG1	no blast match			0		140
38	CL35CONTIG1	GB|AAI22477.1| LOC734151 protein [Xenopus laevis]	AAI22477.1	1,00E-13	37	45/119	453
39	CL36CONTIG1	REF|ZP_01774245.1| conserved hypothetical protein [Geobacter bem...	ZP_01774245.1	1,00E-12	33	76/228	1098
40	CL37CONTIG1	no blast match			0		110
41	CL37CONTIG2	no blast match			0		203
42	CL38CONTIG1	no blast match			0		812
43	CL39CONTIG1	REF|XP_687566.1| PREDICTED: similar to LOC494737 protein [Danio ...	XP_687566.1	5,00E-30	43	67/153	538
44	CL40CONTIG1	no blast match			0		208
45	CL41CONTIG1	REF|NP_001085512.1| MGC80283 protein [Xenopus laevis] >gi|821845...	NP_001085512.1	3,00E-22	40	50/124	371
46	CL42CONTIG1	EMB|CAJ33888.1| putative serine protease inhibitor [Hydra vulgaris]	CAJ33888.1	4,00E-28	93	58/62	209
47	CL43CONTIG1	EMB|CAA33804.1| unnamed protein product [Drosophila melanogaster]	CAA33804.1	6,00E-28	76	60/78	237
48	CL44CONTIG1	no blast match			0		500
49	CL45CONTIG1	REF|NP_001082099.1| beta-amyloid precursor protein A [Xenopus la...	NP_001082099.1	7,00E-06	43	22/51	403
50	CL46CONTIG1	GB|AAL62469.1| ribosomal protein L7 [Spodoptera frugiperda]	AAL62469.1	2,00E-36	77	49/63	337
51	CL47CONTIG1	GB|AAI18129.1| MGC127900 protein [Bos taurus]	AAI18129.1	2,00E-15	62	42/67	207
52	CL48CONTIG1	SP|P51554|EF1A_HYDAT Elongation factor 1-alpha (EF-1-alpha) >gi|...	P51554	6,00E-23	100	51/51	157
53	CL49CONTIG1	REF|NP_001078074.1| ATPDIL2-1/MEE30/UNE5 (PDI-LIKE 2-1, maternal...	NP_001078074.1	6,00E-29	64	57/88	362
54	CL50CONTIG1	REF|NP_001073505.1| hypothetical protein LOC568021 [Danio rerio]...	NP_001073505.1	6,00E-09	33	44/130	369
55	CL51CONTIG1	GB|AAK68766.1| glycine-rich RNA binding protein [Arabidopsis tha...	AAK68766.1	1,00E-08	65	32/49	252
56	CL52CONTIG1	REF|XP_001176123.1| PREDICTED: similar to gelsolin [Strongylocen...	XP_001176123.1	3,00E-40	83	78/93	299
57	CL53CONTIG1	REF|NP_001087877.1| MGC82038 protein [Xenopus laevis] >gi|519500...	NP_001087877.1	3,00E-22	37	49/132	417
58	CL54CONTIG1	no blast match			0		384
59	CL55CONTIG1	no blast match			0		179
60	CL56CONTIG1	no blast match			0		308
61	CL57CONTIG1	GB|AAF24991.1| astacin family metalloendopeptidase FARM-1 [Hydra...	AAF24991.1	8,00E-54	68	102/149	443
62	CL58CONTIG1	REF|NP_001016537.1| aldehyde dehydrogenase 3 family, member A2 [...	NP_001016537.1	2,00E-26	58	54/92	277
63	CL59CONTIG1	no blast match			0		283
64	CL60CONTIG1	REF|XP_694322.2| PREDICTED: similar to Znf622 protein [Danio rerio]	XP_694322.2	4,00E-30	46	69/148	447
65	CL61CONTIG1	REF|XP_001518513.1| PREDICTED: similar to LRRG00134, partial [Or...	XP_001518513.1	4,00E-10	38	32/84	387
66	CL62CONTIG1	GB|ABC25040.1| probable RNA-dependent helicase p72 [Hydra vulgaris]	ABC25040.1	1,00E-53	77	104/135	442
67	CL63CONTIG1	EMB|CAI11750.1| novel protein (zgc:64104) [Danio rerio] >gi|5620...	CAI11750.1	1,00E-15	36	57/157	430
68	CL64CONTIG1	GB|AAW25262.1| unknown [Schistosoma japonicum]	AAW25262.1	1,00E-15	33	41/124	713
69	CL65CONTIG1	REF|XP_316305.3| ENSANGP00000005976 [Anopheles gambiae str. PEST...	XP_316305.3	1,00E-07	29	49/167	428
70	CL66CONTIG1	REF|XP_967635.1| PREDICTED: similar to CG3395-PA, isoform A isof...	XP_967635.1	8,00E-33	85	69/81	259
71	CL67CONTIG1	REF|ZP_01774245.1| conserved hypothetical protein [Geobacter bem...	ZP_01774245.1	3,00E-07	54	27/50	214
72	CL68CONTIG1	no blast match			0		109

73	CL69CONTIG1	REF|XP_973667.1| PREDICTED: similar to CG1507-PA, isoform A [Tri...	XP_973667.1	7,00E-48	46	97/210	707
74	CL70CONTIG1	no blast match			0		115
75	CL71CONTIG1	REF|NP_956951.1| thymocyte nuclear protein 1 [Danio rerio] >gi|8...	NP_956951.1	8,00E-20	56	49/86	294
76	CL72CONTIG1	GB|AAN87350.1| 14-3-3 protein B [Hydra vulgaris]	AAN87350.1	2,00E-61	100	121/121	375
77	CL73CONTIG1	REF|XP_001369327.1| PREDICTED: similar to polyA binding protein ...	XP_001369327.1	8,00E-34	75	67/89	294
78	CL74CONTIG1	GB|AAQ96733.1| bzip transcription factor C/EBP [Podocoryne carnea]	AAQ96733.1	2,00E-36	66	86/129	361
79	CL75CONTIG1	GB|AAX48887.1| S11 [Suberites domuncula]	AAX48887.1	4,00E-22	79	53/67	202
80	CL76CONTIG1	GB|AAR31143.1| zebra precursor; HyZebra [Hydra magnipapillata]	AAR31143.1	9,00E-19	45	44/97	305
81	CL77CONTIG1	no blast match			0		356
82	CL78CONTIG1	GB|AAQ96653.1| glutamate-cysteine ligase modifier subunit [Branc...	AAQ96653.1	2,00E-16	47	42/88	325
83	CL79CONTIG1	SP|Q9BMX5|RS6_APLCA 40S ribosomal protein S6 >gi|12620237|gb|AAG...	Q9BMX5	1,00E-15	71	38/53	206
84	CL80CONTIG1	no blast match			0		416
85	CL81CONTIG1	EMB|CAG08835.1| unnamed protein product [Tetraodon nigroviridis]	CAG08835.1	2,00E-13	67	31/46	210
86	CL82CONTIG1	GB|AAH53776.1| MGC64312 protein [Xenopus laevis]	AAH53776.1	1,00E-19	78	44/56	236
87	CL83CONTIG1	REF|XP_781288.1| PREDICTED: similar to ATPase, H+ transporting, ...	XP_781288.1	6,00E-36	72	73/101	356
88	CL84CONTIG1	REF|NP_001080153.1| actin related protein 2/3 complex, subunit 1...	NP_001080153.1	8,00E-70	64	127/198	636
89	CL85CONTIG1	REF|XP_783224.1| PREDICTED: similar to NADH dehydrogenase (ubiqu...	XP_783224.1	2,00E-39	66	75/113	368
90	CL86CONTIG1	DBJ|BAF45464.1| ribosomal protein S2 [Solea senegalensis] >gi|12...	BAF45464.1	5,00E-22	80	58/72	241
91	CL87CONTIG1	DBJ|BAC92690.1| epitheliopeptide HYM-301 [Hydra magnipapillata]	BAC92690.1	1,00E-10	46	41/88	440
92	CL88CONTIG1	REF|NP_573139.1| CG9742-PA [Drosophila melanogaster] >gi|2942806...	NP_573139.1	2,00E-14	78	37/47	206
93	CL89CONTIG1	REF|XP_414685.1| PREDICTED: similar to betaine homocysteine meth...	XP_414685.1	9,00E-19	68	41/60	184
94	CL90CONTIG1	REF|XP_001509678.1| PREDICTED: similar to SWI/SNF related, matri...	XP_001509678.1	5,00E-49	80	89/111	337
95	CL91CONTIG1	REF|XP_966334.1| PREDICTED: similar to CG3612-PA isoform 1 [Trib...	XP_966334.1	8,00E-75	97	138/141	486
96	CL92CONTIG1	DBJ|BAA34704.1| cathepsin L-like tick cysteine proteinase B [Hae...	BAA34704.1	1,00E-15	83	36/43	456
97	CL93CONTIG1	GB|EDL46410.1| hypothetical protein, conserved [Plasmodium vivax]	EDL46410.1	8,00E-09	37	44/117	365
98	CL94CONTIG1	GB|AAX48841.1| L10e/P0 [Suberites domuncula]	AAX48841.1	7,00E-40	72	82/113	340
99	CL95CONTIG1	GB|AAW26530.1| SJCHGC02843 protein [Schistosoma japonicum]	AAW26530.1	1,00E-14	45	45/98	416
100	CL96CONTIG1	no blast match			0		521
101	CL97CONTIG1	SP|Q4PM47|RS29_IXOSC 40S ribosomal protein S29 >gi|67083965|gb|A...	Q4PM47	2,00E-20	75	41/54	321
102	CL98CONTIG1	GB|ABR23475.1| 60s ribosomal protein L34 [Ornithodoros parkeri]	ABR23475.1	2,00E-37	72	73/101	330
103	CL99CONTIG1	DBJ|BAB16293.1| glyceraldehyde-3-phosphate dehydrogenase [Anguil...	BAB16293.1	2,00E-65	82	122/148	448
104	CL100CONTIG1	REF|XP_866081.1| PREDICTED: similar to Ornithine aminotransferas...	XP_866081.1	9,00E-14	65	36/55	315
105	CL101CONTIG1	no blast match			0		487
106	CL102CONTIG1	REF|XP_001204254.1| PREDICTED: similar to nuclear matrix protein...	XP_001204254.1	3,00E-53	70	96/136	431
107	CL103CONTIG1	no blast match			0		457
108	CL104CONTIG1	no blast match			0		289
109	CL105CONTIG1	REF|NP_001080633.1| lysyl-tRNA synthetase [Xenopus laevis] >gi|2...	NP_001080633.1	1,00E-26	74	46/62	237
110	CL106CONTIG1	no blast match			0		298
111	CL107CONTIG1	EMB|CAJ01880.1| ubiquitin/ribosomal protein S27Ae fusion protein...	CAJ01880.1	6,00E-41	96	84/87	300
112	CL108CONTIG1	no blast match			0		156
113	CL109CONTIG1	no blast match			0		296
114	CL110CONTIG1	no blast match			0		140
115	CL111CONTIG1	no blast match			0		144
116	CL112CONTIG1	REF|NP_001005020.1| MGC79754 protein [Xenopus tropicalis] >gi|49...	NP_001005020.1	3,00E-31	73	61/83	274
117	CL113CONTIG1	no blast match			0		260
118	CL114CONTIG1	no blast match			0		297
119	CL115CONTIG1	REF|NP_001085559.1| MGC80429 protein [Xenopus laevis] >gi|491191...	NP_001085559.1	3,00E-13	41	40/96	300
120	CL116CONTIG1	no blast match			0		279
121	CL117CONTIG1	DBJ|BAB13535.1| hym-323 [Hydra magnipapillata]	BAB13535.1	2,00E-27	93	58/62	297
122	CL118CONTIG1	no blast match			0		283
123	CL119CONTIG1	DBJ|BAF37942.1| Similar to ribosomal protein S23 [Oncorhynchus m...	BAF37942.1	7,00E-19	86	43/50	179
124	CL120CONTIG1	GB|ABN09816.1| Glycoside hydrolase, family 17; X8 [Medicago trun...	ABN09816.1	2,00E-12	38	37/97	282
125	CL121CONTIG1	no blast match			0		135
126	CL122CONTIG1	REF|XP_624951.2| PREDICTED: similar to eIF-5A CG3186-PA, isoform...	XP_624951.2	2,00E-39	78	75/95	289
127	CL123CONTIG1	GB|ABC25042.1| chitinase [Hydra vulgaris]	ABC25042.1	1,00E-11	100	28/28	110
128	CL124CONTIG1	no blast match			0		399
129	CL125CONTIG1	SP|Q25189|GBLP_HYDAT Guanine nucleotide-binding protein subunit ...	Q25189	2,00E-14	100	38/38	149
130	CL127CONTIG1	no blast match			0		107
131	CL128CONTIG1	no blast match			0		294
132	CL129CONTIG1	EMB|CAA08792.1| ribosomal protein L9 [Podocoryne carnea]	CAA08792.1	5,00E-16	81	39/48	147
133	CL130CONTIG1	no blast match			0		313
134	CL131CONTIG1	no blast match			0		297
135	CL132CONTIG1	no blast match			0		296
136	CL133CONTIG1	REF|XP_001177412.1| PREDICTED: similar to interferon, gamma-indu...	XP_001177412.1	3,00E-20	48	46/94	289
137	CL134CONTIG1	no blast match			0		123
138	CL135CONTIG1	no blast match			0		236
139	CL136CONTIG1	no blast match			0		124
140	CL137CONTIG1	EMB|CAA04046.1| actin [Helobdella triserialis]	CAA04046.1	8,00E-28	96	61/63	192
141	CL138CONTIG1	PDB|1SP7|A Chain A, Structure Of The Cys-Rich C-Terminal Domain ...	1SP7	1,00E-06	78	18/23	193
142	CL139CONTIG1	no blast match			0		165
143	CL140CONTIG1	no blast match			0		191
144	CL141CONTIG1	no blast match			0		183
145	CL142CONTIG1	GB|ABM55461.1| ribosomal protein L19 [Xenopsylla cheopis]	ABM55461.1	6,00E-22	72	47/65	442
146	CL143CONTIG1	no blast match			0		428
147	CL144CONTIG1	REF|XP_001022964.1| TPR Domain containing protein [Tetrahymena t...	XP_001022964.1	6,00E-23	28	60/213	756
148	CL145CONTIG1	SP|P17126|ACT_HYDAT Actin, non-muscle 6.2 >gi|159254|gb|AAA29205...	P17126	1,00E-35	100	75/75	228
149	CL146CONTIG1	no blast match			0		195
150	CL147CONTIG1	GB|AAO60428.1| chordin-like protein [Hydra magnipapillata]	AAO60428.1	7,00E-37	100	69/69	211
151	CL148CONTIG1	REF|XP_761629.1| hypothetical protein UM05482.1 [Ustilago maydis...	XP_761629.1	3,00E-20	70	50/71	214
152	CL149CONTIG1	no blast match			0		215
153	CL150CONTIG1	GB|AAG30574.1|AF312857_1 latrophilin-related protein [Hydra vulg...	AAG30574.1	4,00E-06	59	22/37	188
154	CL151CONTIG1	REF|XP_001342834.1| PREDICTED: similar to 60S ribosomal protein ...	XP_001342834.1	3,00E-11	79	35/44	147
155	CL152CONTIG1	REF|NP_001011190.1| hypothetical protein LOC496612 [Xenopus trop...	NP_001011190.1	1,00E-10	45	37/82	270
156	CL153CONTIG1	REF|XP_001497791.1| PREDICTED: similar to ubiquitin conjugating ...	XP_001497791.1	2,00E-38	79	71/89	339
157	CL154CONTIG1	no blast match			0		265
158	CL155CONTIG1	no blast match			0		271
159	CL156CONTIG1	REF|NP_001088541.1| hypothetical protein LOC495415 [Xenopus laev...	NP_001088541.1	4,00E-15	61	41/67	319
160	CL157CONTIG1	no blast match			0		109
161	CL158CONTIG1	no blast match			0		146
162	CL159CONTIG1	GB|AAY66836.1| ribosomal protein L37A [Ixodes scapularis]	AAY66836.1	1,00E-14	79	35/44	169

163	CL160CONTIG1	GB|AAR85887.1| non-muscle actin [Hydra viridis]	AAR85887.1	4,00E-24	98	53/54	162
164	CL161CONTIG1	no blast match			0		138
165	CL162CONTIG1	no blast match			0		218
166	CL163CONTIG1	no blast match			0		304
167	CL164CONTIG1	GB|AAN05585.1| ribosomal protein L22 [Argopecten irradians]	AAN05585.1	3,00E-16	80	38/47	155
168	CL165CONTIG1	no blast match			0		210
169	CL166CONTIG1	no blast match			0		138
170	CL167CONTIG1	no blast match			0		218
171	CL168CONTIG1	no blast match			0		147
172	CL169CONTIG1	REF|XP_001363619.1| PREDICTED: similar to eukaryotic translation...	XP_001363619.1	5,00E-35	72	70/97	339
173	CL170CONTIG1	no blast match			0		391
174	CL171CONTIG1	REF|XP_001175487.1| PREDICTED: similar to peptidylprolyl isomera...	XP_001175487.1	4,00E-41	74	82/110	430
175	CL172CONTIG1	REF|XP_780685.2| PREDICTED: similar to cysteine-rich repeat-cont...	XP_780685.2	9,00E-13	39	34/86	338
176	CL173CONTIG1	GB|ABR19841.1| minicollagen-15 [Hydra vulgaris]	ABR19841.1	5,00E-76	99	126/127	382
177	CL174CONTIG1	no blast match			0		428
178	CL175CONTIG1	no blast match			0		425
179	CL176CONTIG1	no blast match			0		414
180	CL177CONTIG1	GB|EAT43097.1| mitochondrial processing peptidase beta subunit [...	EAT43097.1	9,00E-43	56	81/143	430
181	CL178CONTIG1	no blast match			0		475
182	CL179CONTIG1	DBJ|BAB13308.1| vasa-related protein CnVAS2 [Hydra magnipapillata]	BAB13308.1	2,00E-30	100	61/61	425
183	CL180CONTIG1	GB|AAH24126.1| Dmgdh protein [Mus musculus]	AAH24126.1	3,00E-20	53	43/81	452
184	CL181CONTIG1	GB|ABD15177.1| innexin 1 [Hydra vulgaris]	ABD15177.1	2,00E-27	45	63/137	419
185	CL182CONTIG1	GB|EAT44074.1| preprotein translocase secy subunit (sec61) [Aede...	EAT44074.1	1,00E-51	77	101/131	391
186	CL183CONTIG1	REF|XP_001490432.1| PREDICTED: similar to DnaJ protein [Equus ca...	XP_001490432.1	1,00E-43	59	83/140	418
187	CL184CONTIG1	no blast match			0		371
188	CL185CONTIG1	EMB|CAN67061.1| hypothetical protein [Vitis vinifera]	CAN67061.1	7,00E-06	44	27/61	314
189	CL186CONTIG1	GB|AAS01181.1| Cniwi [Podocoryne carnea]	AAS01181.1	4,00E-39	73	76/104	314
190	CL187CONTIG1	REF|NP_001080575.1| interleukin enhancer binding factor 2, 45kDa...	NP_001080575.1	1,00E-39	62	77/123	369
191	CL188CONTIG1	EMB|CAM37732.1| flagellar calcium-binding protein, putative [Lei...	CAM37732.1	8,00E-07	34	35/102	321
192	CL189CONTIG1	REF|ZP_01303848.1| glycine cleavage system protein H [Sphingomon...	ZP_01303848.1	2,00E-19	63	43/68	320
193	CL190CONTIG1	SP|P38976|RAS2_HYDMA Ras-like protein RAS2 precursor >gi|11140|e...	P38976	1,00E-19	100	50/50	335
194	CL191CONTIG1	REF|XP_001519193.1| PREDICTED: similar to dynein, light chain, L...	XP_001519193.1	2,00E-08	96	27/28	299
195	CL192CONTIG1	no blast match			0		337
196	CL193CONTIG1	no blast match			0		326
197	CL194CONTIG1	no blast match			0		382
198	CL195CONTIG1	EMB|CAG03540.1| unnamed protein product [Tetraodon nigroviridis]	CAG03540.1	1,00E-47	87	94/108	326
199	CL196CONTIG1	no blast match			0		425
200	CL197CONTIG1	REF|NP_001005390.1| calpain, small subunit 1 [Danio rerio] >gi|4...	NP_001005390.1	2,00E-34	39	79/201	578
201	CL198CONTIG1	no blast match			0		494
202	CL199CONTIG1	no blast match			0		577
203	CL200CONTIG1	REF|XP_001373181.1| PREDICTED: hypothetical protein [Monodelphis...	XP_001373181.1	8,00E-28	46	63/135	453
204	CL201CONTIG1	no blast match			0		480
205	CL202CONTIG1	DBJ|BAE60632.1| unnamed protein product [Aspergillus oryzae]	BAE60632.1	5,00E-09	28	35/123	519
206	CL203CONTIG1	REF|XP_001376908.1| PREDICTED: hypothetical protein [Monodelphis...	XP_001376908.1	3,00E-10	30	52/168	568
207	CL204CONTIG1	GB|AAN71256.1| LD34409p [Drosophila melanogaster]	AAN71256.1	4,00E-66	73	129/175	923
208	CL205CONTIG1	no blast match			0		568
209	CL206CONTIG1	REF|XP_797203.2| PREDICTED: similar to rhamnose-binding lectin (...	XP_797203.2	2,00E-33	42	77/183	603
210	CL207CONTIG1	DBJ|BAA88337.1| ORF2 [Platemys spixii]	BAA88337.1	7,00E-09	34	37/107	614
211	CL208CONTIG1	EMB|CAF90372.1| unnamed protein product [Tetraodon nigroviridis]	CAF90372.1	5,00E-50	60	92/151	531
212	CL209CONTIG1	GB|AAC31964.1| plasminogen-related serine protease [Hydra vulgaris]	AAC31964.1	8,00E-26	86	51/59	356
213	CL210CONTIG1	no blast match			0		458
214	CL211CONTIG1	REF|XP_001234035.1| PREDICTED: similar to Alport syndrome, menta...	XP_001234035.1	2,00E-10	75	24/32	434
215	CL212CONTIG1	no blast match			0		410
216	CL213CONTIG1	REF|XP_606010.3| PREDICTED: hypothetical protein [Bos taurus]	XP_606010.3	1,00E-42	59	82/137	414
217	CL214CONTIG1	REF|YP_503988.1| hypothetical protein Mhun_2572 [Methanospirillu...	YP_503988.1	9,00E-08	38	22/57	480
218	CL215CONTIG1	REF|XP_872039.1| PREDICTED: similar to histidase [Bos taurus]	XP_872039.1	1,00E-100	77	178/231	695
219	CL216CONTIG1	REF|XP_797462.2| PREDICTED: similar to beta1-syntrophin [Strongy...	XP_797462.2	4,00E-42	54	77/141	402
220	CL217CONTIG1	REF|NP_997684.1| translocase of outer mitochondrial membrane 70 ...	NP_997684.1	7,00E-32	54	66/121	401
221	CL218CONTIG1	REF|XP_795871.1| PREDICTED: similar to De-etiolated homolog 1 (A...	XP_795871.1	8,00E-47	57	89/156	476
222	CL219CONTIG1	GB|EDN28930.1| predicted protein [Botryotinia fuckeliana B05.10]	EDN28930.1	4,00E-11	37	45/119	474
223	CL220CONTIG1	GB|AAA29218.2| tyrosine kinase receptor [Hydra vulgaris]	AAA29218.2	3,00E-16	32	50/155	524
224	CL221CONTIG1	REF|XP_786637.1| PREDICTED: hypothetical protein [Strongylocentr...	XP_786637.1	9,00E-20	67	40/59	507
225	CL222CONTIG1	no blast match			0		464
226	CL223CONTIG1	no blast match			0		460
227	CL224CONTIG1	no blast match			0		231
228	CL225CONTIG1	GB|AAP92326.1| IAP-associated factor Viaf1 [Branchiostoma belche...	AAP92326.1	3,00E-59	61	110/178	543
229	CL226CONTIG1	EMB|CAD91421.1| ribosomal protein L5 [Crassostrea gigas]	CAD91421.1	5,00E-25	75	56/74	226
230	CL227CONTIG1	no blast match			0		301
231	CL228CONTIG1	GB|EAW49948.1| KIAA0690, isoform CRA_c [Homo sapiens]	EAW49948.1	8,00E-17	68	41/60	208
232	CL229CONTIG1	GB|AAR24460.1| tropomyosin [Nematostella vectensis]	AAR24460.1	3,00E-14	43	41/95	370
233	CL230CONTIG1	GB|AAG30574.1|AF312857_1 latrophilin-related protein [Hydra vulg...	AAG30574.1	1,00E-06	57	23/40	188
234	CL231CONTIG1	REF|XP_794884.2| PREDICTED: similar to putative porin precursor ...	XP_794884.2	5,00E-07	35	28/78	233
235	CL232CONTIG1	no blast match			0		355
236	CL233CONTIG1	GB|AAR31143.1| zebra precursor; HyZebra [Hydra magnipapillata]	AAR31143.1	5,00E-39	100	81/81	255
237	CL234CONTIG1	GB|ABG46423.1| ribosomal protein S26 [Pectinaria gouldii]	ABG46423.1	3,00E-25	91	55/60	181
238	CL235CONTIG1	no blast match			0		232
239	CL236CONTIG1	REF|XP_788786.2| PREDICTED: similar to matrix metalloproteinase ...	XP_788786.2	2,00E-09	56	28/50	166
240	CL237CONTIG1	REF|NP_001089459.1| hypothetical protein LOC734509 [Xenopus laev...	NP_001089459.1	6,00E-33	82	64/78	248
241	CL238CONTIG1	REF|XP_395013.2| PREDICTED: similar to GTP-binding protein CG252...	XP_395013.2	7,00E-48	81	95/116	352
242	CL239CONTIG1	no blast match			0		281
243	CL240CONTIG1	no blast match			0		281
244	CL241CONTIG1	no blast match			0		291
245	CL242CONTIG1	no blast match			0		421
246	CL243CONTIG1	GB|EAT35698.1| activin receptor type I, putative [Aedes aegypti]	EAT35698.1	4,00E-36	52	73/139	442
247	CL244CONTIG1	no blast match			0		307
248	CL245CONTIG1	no blast match			0		217
249	CL246CONTIG1	REF|XP_001364734.1| PREDICTED: similar to glyceraldehyde-3-phosp...	XP_001364734.1	3,00E-21	68	49/72	220
250	CL247CONTIG1	no blast match			0		220
251	CL248CONTIG1	no blast match			0		305
252	CL249CONTIG1	GB|AAO86696.1| selenoprotein W1 [Danio rerio]	AAO86696.1	2,00E-15	53	38/71	312

253	CL250CONTIG1	no blast match			0		231
254	CL251CONTIG1	REF|XP_310465.2| ENSANGP00000007334 [Anopheles gambiae str. PEST...	XP_310465.2	3,00E-34	92	74/80	242
255	CL252CONTIG1	no blast match			0		371
256	CL253CONTIG1	GB|AAN87349.1| 14-3-3 protein A [Hydra vulgaris]	AAN87349.1	2,00E-20	92	51/55	392
257	CL254CONTIG1	REF|NP_001088024.1| hypothetical protein LOC494715 [Xenopus laev...	NP_001088024.1	1,00E-40	63	79/124	378
258	CL255CONTIG1	REF|NP_997766.1| survival motor neuron domain containing 1 [Dani...	NP_997766.1	2,00E-13	56	44/78	368
259	CL256CONTIG1	REF|XP_001376304.1| PREDICTED: similar to transmembrane protease...	XP_001376304.1	5,00E-24	46	55/118	379
260	CL257CONTIG1	no blast match			0		398
261	CL258CONTIG1	GB|AAR09674.1| similar to Drosophila melanogaster RpS25 [Drosoph...	AAR09674.1	4,00E-37	71	83/116	415
262	CL259CONTIG1	REF|NP_001080783.1| transgelin 2 [Xenopus laevis] >gi|28436827|g...	NP_001080783.1	4,00E-20	43	50/116	390
263	CL260CONTIG1	REF|NP_001085851.1| MGC80911 protein [Xenopus laevis] >gi|491155...	NP_001085851.1	2,00E-14	60	36/60	307
264	CL261CONTIG1	REF|XP_797203.2| PREDICTED: similar to rhamnose-binding lectin (...	XP_797203.2	6,00E-17	50	43/85	318
265	CL262CONTIG1	no blast match			0		401
266	CL263CONTIG1	EMB|CAL36995.1| chitinase 2 [Hydractinia echinata]	CAL36995.1	2,00E-68	62	121/194	585
267	CL264CONTIG1	no blast match			0		364
268	CL265CONTIG1	REF|NP_001011456.1| eukaryotic translation initiation factor 2, ...	NP_001011456.1	2,00E-57	84	106/125	379
269	CL266CONTIG1	REF|XP_643401.1| hypothetical protein DDBDRAFT_0167292 [Dictyost...	XP_643401.1	2,00E-16	50	37/73	246
270	CL267CONTIG1	no blast match			0		247
271	CL268CONTIG1	no blast match			0		260
272	CL269CONTIG1	no blast match			0		236
273	CL270CONTIG1	no blast match			0		238
274	CL271CONTIG1	GB|ABC25037.1| ribosomal protein S19 [Hydra vulgaris]	ABC25037.1	1,00E-38	98	80/81	244
275	CL272CONTIG1	no blast match			0		286
276	CL273CONTIG1	REF|XP_967161.1| PREDICTED: similar to CG8053-PA, isoform A [Tri...	XP_967161.1	4,00E-48	90	90/99	347
277	CL274CONTIG1	REF|XP_640451.1| hypothetical protein DDBDRAFT_0204239 [Dictyost...	XP_640451.1	3,00E-11	75	31/41	357
278	CL275CONTIG1	no blast match			0		330
279	CL276CONTIG1	no blast match			0		264
280	CL277CONTIG1	no blast match			0		333
281	CL278CONTIG1	no blast match			0		347
282	CV284052.1	no blast match			0		354
283	CV284056.1	no blast match			0		271
284	CV284060.1	GB|ABC96783.1| carbonic anhydrase FCA-b [Fungia scutaria]	ABC96783.1	9,00E-11	36	40/111	368
285	CV284061.1	no blast match			0		160
286	CV284064.1	no blast match			0		295
287	CV284066.1	no blast match			0		267
288	CV284069.1	REF|XP_214199.3| PREDICTED: similar to RANBP4 [Rattus norvegicus...	XP_214199.3	1,00E-09	43	35/80	326
289	CV284071.1	REF|XP_418682.1| PREDICTED: similar to MGC83241 protein [Gallus ...	XP_418682.1	5,00E-33	50	67/132	545
290	CV284085.1	no blast match			0		140
291	CV284087.1	no blast match			0		125
292	CV284091.1	no blast match			0		349
293	CV284092.1	no blast match			0		359
294	CV284093.1	no blast match			0		232
295	CV284104.1	no blast match			0		111
296	CV284106.1	EMB|CAN70956.1| hypothetical protein [Vitis vinifera]	CAN70956.1	2,00E-13	35	37/103	467
297	CV284110.1	no blast match			0		231
298	CV284115.1	no blast match			0		313
299	CV284116.1	REF|XP_001333195.1| PREDICTED: similar to Adrm1b protein [Danio ...	XP_001333195.1	2,00E-07	52	32/61	212
300	CV284122.1	REF|XP_001178247.1| PREDICTED: hypothetical protein [Strongyloce...	XP_001178247.1	4,00E-08	47	27/57	319
301	CV284126.1	no blast match			0		515
302	CV284127.1	GB|AAO60428.1| chordin-like protein [Hydra magnipapillata]	AAO60428.1	9,00E-08	33	32/95	371
303	CV284136.1	REF|NP_999767.1| metalloproteinase SpAN [Strongylocentrotus purp...	NP_999767.1	1,00E-21	44	60/136	389
304	CV284143.1	REF|XP_795093.2| PREDICTED: hypothetical protein [Strongylocentr...	XP_795093.2	4,00E-13	52	32/61	382
305	CV284144.1	REF|XP_972804.1| PREDICTED: similar to CG30185-PA [Tribolium cas...	XP_972804.1	2,00E-08	30	37/123	438
306	CV284147.1	no blast match			0		248
307	CV284149.1	REF|XP_001335539.1| PREDICTED: similar to mannosyl (alpha-1,6-)-...	XP_001335539.1	2,00E-08	50	27/53	313
308	CV284150.1	REF|NP_718081.1| zinc-dependent metallopeptidase [Shewanella one...	NP_718081.1	6,00E-09	46	26/56	312
309	CV284155.1	no blast match			0		230
310	CV284156.1	GB|ABC25042.1| chitinase [Hydra vulgaris]	ABC25042.1	5,00E-22	82	47/57	255
311	CV284164.1	no blast match			0		353
312	CV284165.1	REF|NP_001069850.1| microsomal glutathione S-transferase 2 [Bos ...	NP_001069850.1	1,00E-17	50	43/86	339
313	CV284166.1	no blast match			0		174
314	CV284167.1	REF|XP_001199439.1| PREDICTED: hypothetical protein [Strongyloce...	XP_001199439.1	7,00E-16	38	41/107	332
315	CV284171.1	REF|NP_001041438.1| hypothetical protein LOC503325 [Rattus norve...	NP_001041438.1	7,00E-07	39	21/53	381
316	CV284172.1	no blast match			0		291
317	CV284175.1	SP|P38977|ANTA_HYDMA Antistasin precursor (ATS) (Blood coagulati...	P38977	1,00E-20	69	44/63	220
318	CV284176.1	no blast match			0		287
319	CV284178.1	no blast match			0		158
320	CV284180.1	SP|P51554|EF1A_HYDAT Elongation factor 1-alpha (EF-1-alpha) >gi|...	P51554	2,00E-47	100	91/91	362
321	CV284181.1	REF|NP_001029440.1| DnaJ (Hsp40) homolog, subfamily B, member 11...	NP_001029440.1	8,00E-06	58	23/39	505
322	CV284182.1	no blast match			0		305
323	CV284183.1	REF|NP_001085392.1| MGC79007 protein [Xenopus laevis] >gi|487346...	NP_001085392.1	2,00E-10	62	31/50	246
324	CV284186.1	REF|XP_001306517.1| variable membrane protein precursor, putativ...	XP_001306517.1	1,00E-06	21	42/198	531
325	CV284187.1	REF|XP_551775.2| ENSANGP00000026077 [Anopheles gambiae str. PEST...	XP_551775.2	2,00E-28	56	60/107	378
326	CV284189.1	REF|XP_791196.1| PREDICTED: similar to 3-hydroxyisobutyryl-Coenz...	XP_791196.1	7,00E-14	44	37/83	249
327	CV284191.1	GB|AAT06123.1| fructose-bisphosphate aldolase [Obelia sp. KJP-2004]	AAT06123.1	4,00E-33	83	69/83	255
328	CV284192.1	GB|AAX48900.1| S24 [Suberites domuncula]	AAX48900.1	4,00E-11	81	30/37	132
329	CV284197.1	no blast match			0		539
330	CV284199.1	no blast match			0		165
331	CV284200.1	REF|XP_001341283.1| PREDICTED: similar to cysteine-rich motor ne...	XP_001341283.1	6,00E-07	46	21/45	291
332	CV284202.1	REF|NP_001007880.1| shmt2 protein [Xenopus tropicalis] >gi|51259...	NP_001007880.1	3,00E-34	70	73/103	309
333	CV284212.1	REF|YP_054549.1| cytochrome c oxidase subunit III [Squilla manti...	YP_054549.1	4,00E-34	66	74/111	334
334	CV284216.1	REF|NP_001032653.1| hypothetical protein LOC641566 [Danio rerio]...	NP_001032653.1	2,00E-53	83	99/118	356
335	CV284219.1	no blast match			0		129
336	CV284222.1	DBJ|BAD92713.1| importin 4 variant [Homo sapiens]	BAD92713.1	2,00E-14	48	30/62	327
337	CV284228.1	no blast match			0		103
338	CV284235.1	REF|XP_312404.2| ENSANGP00000022132 [Anopheles gambiae str. PEST...	XP_312404.2	5,00E-35	68	75/110	329
339	CV284239.1	no blast match			0		443
340	CV284251.1	DBJ|BAF65668.1| tubular mastigoneme protein [Ochromonas danica]	BAF65668.1	8,00E-26	66	56/84	382
341	CV284263.1	no blast match			0		194
342	CV284267.1	GB|AAR01286.1| elongation factor-2 [Ctenolepisma lineata]	AAR01286.1	3,00E-26	86	53/61	190

343	CV284281.1	no blast match			0		211
344	CV284282.1	REF|XP_394752.2| PREDICTED: similar to Actin-related protein 8 i...	XP_394752.2	3,00E-12	62	31/50	394
345	CV284296.1	no blast match			0		279
346	CV284299.1	no blast match			0		194
347	CV284301.1	no blast match			0		156
348	CV284310.1	REF|XP_001518180.1| PREDICTED: similar to alpha NAC/1.9.2. prote...	XP_001518180.1	6,00E-18	84	45/53	160
349	CV284648.1	GB|AAH06719.1| 2900073G15Rik protein [Mus musculus]	AAH06719.1	6,00E-29	76	61/80	433
350	CV284649.1	EMB|CAF94178.1| unnamed protein product [Tetraodon nigroviridis]	CAF94178.1	2,00E-37	72	74/102	395
351	CV284651.1	REF|XP_001342769.1| PREDICTED: hypothetical protein LOC449654 [D...	XP_001342769.1	4,00E-14	56	37/65	267
352	CV284652.1	no blast match			0		305
353	CV284653.1	EMB|CAE57636.1| Hypothetical protein CBG00621 [Caenorhabditis br...	CAE57636.1	9,00E-30	57	61/106	321
354	CV284660.1	REF|XP_865910.1| PREDICTED: similar to Malate dehydrogenase, cyt...	XP_865910.1	5,00E-06	53	26/49	211
355	CV284663.1	GB|ABC25036.1| ribosomal protein S9 [Hydra vulgaris]	ABC25036.1	1,00E-11	100	32/32	260
356	CV284670.1	no blast match			0		472
357	CV284676.1	no blast match			0		277
358	CV284677.1	no blast match			0		215
359	CV284680.1	EMB|CAF91608.1| unnamed protein product [Tetraodon nigroviridis]	CAF91608.1	3,00E-09	56	27/48	147
360	CV284682.1	GB|EAT48185.1| platelet-activating factor acetylhydrolase isofor...	EAT48185.1	2,00E-15	72	36/50	155
361	CV284684.1	no blast match			0		391
362	CV284685.1	no blast match			0		481
363	CV284688.1	REF|NP_001080153.1| actin related protein 2/3 complex, subunit 1...	NP_001080153.1	6,00E-65	63	122/192	574
364	CV284690.1	no blast match			0		452
365	CV284698.1	no blast match			0		256
366	CV284703.1	no blast match			0		249
367	CV284705.1	GB|AAH41538.1| LOC398543 protein [Xenopus laevis]	AAH41538.1	3,00E-09	35	37/104	378
368	CV284706.1	no blast match			0		282
369	CV284707.1	EMB|CAC81066.1| putative cyclosporin A-binding protein [Picea ab...	CAC81066.1	1,00E-25	86	53/61	187
370	CV284708.1	no blast match			0		271
371	CV284712.1	REF|XP_001189047.1| PREDICTED: similar to rhamnose-binding lecti...	XP_001189047.1	2,00E-15	50	43/85	259
372	CV284713.1	no blast match			0		144
373	CV284717.1	REF|XP_001198770.1| PREDICTED: hypothetical protein, partial [St...	XP_001198770.1	7,00E-11	58	35/60	201
374	CV284718.1	EMB|CAJ33888.1| putative serine protease inhibitor [Hydra vulgaris]	CAJ33888.1	9,00E-11	76	30/39	190
375	CV284721.1	REF|XP_783617.2| PREDICTED: similar to membrane alanine aminopep...	XP_783617.2	3,00E-14	35	39/109	328
376	CV284734.1	GB|AAR31143.1| zebra precursor; HyZebra [Hydra magnipapillata]	AAR31143.1	9,00E-07	38	17/44	343
377	CV284741.1	no blast match			0		300
378	CV284745.1	REF|NP_001084775.1| hypothetical protein LOC431811 [Xenopus laev...	NP_001084775.1	1,00E-30	80	62/77	277
379	CV284746.1	no blast match			0		384
380	CV284751.1	no blast match			0		111
381	CV284756.1	SP|Q08699|RS14_PODCA 40S ribosomal protein S14 >gi|396252|emb|CA...	Q08699	5,00E-61	94	119/126	410
382	CV284757.1	EMB|CAJ33888.1| putative serine protease inhibitor [Hydra vulgaris]	CAJ33888.1	4,00E-12	85	30/35	396
383	CV284762.1	no blast match			0		184
384	CV284763.1	EMB|CAG07935.1| unnamed protein product [Tetraodon nigroviridis]	CAG07935.1	9,00E-08	40	26/64	280
385	CV284764.1	GB|AAI30057.1| LOC495430 protein [Xenopus laevis]	AAI30057.1	9,00E-27	58	55/94	308
386	CV284766.1	GB|ABQ22499.1| vacuolar ATP synthase 16 kDa proteolipid subunit-...	ABQ22499.1	9,00E-16	93	44/47	192
387	CV284767.1	REF|XP_696748.2| PREDICTED: hypothetical protein [Danio rerio]	XP_696748.2	6,00E-34	62	61/97	293
388	CV284769.1	no blast match			0		339
389	CV284771.1	GB|AAX48842.1| L10a [Suberites domuncula]	AAX48842.1	1,00E-19	77	47/61	184
390	CV284772.1	no blast match			0		375
391	CV284781.1	no blast match			0		339
392	CV284787.1	no blast match			0		150
393	CV284792.1	no blast match			0		462
394	CV284796.1	no blast match			0		180
395	CV284797.1	no blast match			0		239
396	CV284798.1	no blast match			0		358
397	CV284805.1	GB|ABC25033.1| ribosomal protein L35 [Hydra vulgaris]	ABC25033.1	5,00E-12	78	39/50	297
398	CV284810.1	no blast match			0		433
399	CV284813.1	DBJ|BAB13307.1| vasa-related protein CnVAS1 [Hydra magnipapillata]	BAB13307.1	6,00E-42	100	87/87	264
400	CV284814.1	no blast match			0		337
401	CV284819.1	no blast match			0		160
402	CV284820.1	SP|P38984|RSSA_CHLVR 40S ribosomal protein SA (p40) (33 kDa lami...	P38984	3,00E-79	94	144/153	476
403	CV284827.1	REF|XP_677983.1| nucleoside diphosphate kinase b; [Plasmodium be...	XP_677983.1	4,00E-11	71	32/45	227
404	CV284828.1	REF|NP_001085935.1| MGC82844 protein [Xenopus laevis] >gi|492561...	NP_001085935.1	3,00E-06	80	24/30	113
405	CV284829.1	REF|XP_001491740.1| PREDICTED: similar to DEAH (Asp-Glu-Ala-His)...	XP_001491740.1	3,00E-41	84	83/98	299
406	CV284831.1	no blast match			0		445
407	CV284834.1	REF|NP_001075331.1| ubiquitin [Equus caballus] >gi|21070215|gb|A...	NP_001075331.1	5,00E-19	66	46/69	208
408	CV284839.1	no blast match			0		566
409	CV284843.1	REF|XP_356642.1| PREDICTED: similar to ribosomal protein L10a [M...	XP_356642.1	4,00E-12	82	39/47	358
410	CV284846.1	GB|EAT46335.1| DEAD box ATP-dependent RNA helicase [Aedes aegypti]	EAT46335.1	4,00E-19	56	43/76	404
411	CV284848.1	no blast match			0		228
412	CV284855.1	no blast match			0		122
413	CV284856.1	DBJ|BAF45891.1| ribosomal protein S3a [Solea senegalensis]	BAF45891.1	2,00E-10	54	31/57	179
414	CV284858.1	REF|XP_797404.1| PREDICTED: similar to C20orf18 [Strongylocentro...	XP_797404.1	2,00E-06	36	30/83	384
415	CV284879.1	PIR||B41132 collagen-related protein 2 - Hydra magnipapillata (f...		4,00E-14	100	38/38	123
416	CV284880.1	no blast match			0		145
417	CV284885.1	no blast match			0		120
418	CV284894.1	no blast match			0		111
419	CV284914.1	REF|NP_001040276.1| ribosomal protein L7Ae [Bombyx mori] >gi|872...	NP_001040276.1	9,00E-11	82	33/40	123
420	CV284922.1	EMB|CAJ33888.1| putative serine protease inhibitor [Hydra vulgaris]	CAJ33888.1	3,00E-26	72	52/72	217
421	CV284943.1	EMB|CAG10369.1| unnamed protein product [Tetraodon nigroviridis]	CAG10369.1	1,00E-11	36	28/76	231
422	CV284948.1	no blast match			0		118
423	CV284953.1	no blast match			0		253
424	CV284957.1	EMB|CAJ33888.1| putative serine protease inhibitor [Hydra vulgaris]	CAJ33888.1	6,00E-50	71	87/122	421
425	CV284963.1	no blast match			0		247
426	CV284988.1	REF|XP_001371488.1| PREDICTED: hypothetical protein [Monodelphis...	XP_001371488.1	9,00E-07	51	25/49	191
427	CV284996.1	no blast match			0		113
428	CV285005.1	no blast match			0		429
429	CV285012.1	no blast match			0		500
430	CV285022.1	SP|Q98SN8|S61A2_ONCMY Protein transport protein Sec61 subunit al...	Q98SN8	2,00E-09	90	30/33	100
431	CV285030.1	no blast match			0		187
432	CV285046.1	REF|XP_001520202.1| PREDICTED: similar to RBM25 protein [Ornitho...	XP_001520202.1	4,00E-11	73	33/45	205

433	CV285047.1	no blast match			0		141
434	CV285742.1	REF|XP_597181.3| PREDICTED: similar to vacuolar proton pump 116 ...	XP_597181.3	2,00E-39	75	77/102	310
435	CV285754.1	no blast match			0		199
436	CV285766.1	no blast match			0		153
437	CV285771.1	no blast match			0		398
438	CV285792.1	REF|NP_733249.1| Heterogeneous nuclear ribonucleoprotein at 98DE...	NP_733249.1	3,00E-08	40	32/80	310
439	CV285804.1	no blast match			0		157
440	CV285805.1	no blast match			0		277
441	CV285815.1	GB|AAX48838.1| L7a [Suberites domuncula]	AAX48838.1	5,00E-15	71	42/59	194
442	CV285823.1	REF|XP_001197762.1| PREDICTED: similar to NAD(P)H:quinone oxidor...	XP_001197762.1	1,00E-20	37	56/150	530
443	CV285824.1	GB|AAX09926.1| putative tyrosine-rich heat shock protein [Aureli...	AAX09926.1	8,00E-15	63	33/52	178
444	CV285825.1	no blast match			0		242
445	CV285826.1	no blast match			0		361
446	CV285828.1	REF|XP_001203886.1| PREDICTED: similar to Conserved oligomeric G...	XP_001203886.1	2,00E-23	63	58/91	294
447	CV285829.1	REF|ZP_01774245.1| conserved hypothetical protein [Geobacter bem...	ZP_01774245.1	4,00E-15	42	72/170	816
448	CV285834.1	EMB|CAG02225.1| unnamed protein product [Tetraodon nigroviridis]	CAG02225.1	2,00E-47	73	88/120	390
449	CV285840.1	no blast match			0		187
450	CV285841.1	no blast match			0		375
451	CV285844.1	no blast match			0		191
452	CV285845.1	no blast match			0		103
453	CV285846.1	DBJ|BAB13308.1| vasa-related protein CnVAS2 [Hydra magnipapillata]	BAB13308.1	3,00E-43	96	88/91	277
454	CV285868.1	GB|AAX30301.1| unknown [Schistosoma japonicum]	AAX30301.1	9,00E-19	88	44/50	215
455	CV285881.1	GB|EAT44394.1| oligosaccharyl transferase [Aedes aegypti]	EAT44394.1	4,00E-58	88	104/117	359
456	CV285885.1	no blast match			0		413
457	CV285887.1	no blast match			0		291
458	CV285892.1	GB|AAY66953.1| ribosomal protein L36 [Ixodes scapularis]	AAY66953.1	2,00E-22	69	49/71	253
459	CV285896.1	no blast match			0		397
460	CV285898.1	REF|XP_966465.1| PREDICTED: similar to CG13391-PA, isoform A [Tr...	XP_966465.1	1,00E-26	62	59/94	284
461	CV285908.1	REF|XP_857983.1| PREDICTED: similar to N-acetyltransferase-like ...	XP_857983.1	1,00E-06	42	32/75	324
462	CV285912.1	EMB|CAG08643.1| unnamed protein product [Tetraodon nigroviridis]	CAG08643.1	1,00E-07	47	39/82	570
463	CV285920.1	REF|NP_001016282.1| heat shock protein 90kDa alpha (cytosolic), ...	NP_001016282.1	4,00E-27	65	59/90	341
464	CV285922.1	no blast match			0		126
465	CV285934.1	REF|NP_787020.1| NADH dehydrogenase (ubiquinone) 1 alpha subcomp...	NP_787020.1	2,00E-21	45	49/108	604
466	CV285937.1	REF|XP_001478155.1| PREDICTED: hypothetical protein [Mus musculus]	XP_001478155.1	2,00E-08	33	49/146	944
467	CV285942.1	GB|AAF24991.1| astacin family metalloendopeptidase FARM-1 [Hydra...	AAF24991.1	3,00E-29	60	60/99	326
468	CV285945.1	REF|NP_001016978.1| archain [Xenopus tropicalis] >gi|89271953|em...	NP_001016978.1	6,00E-29	61	57/92	278
469	CV285947.1	REF|NP_001014734.1| cacophony CG1522-PH, isoform H [Drosophila m...	NP_001014734.1	1,00E-08	55	32/58	229
470	CV285951.1	no blast match			0		334
471	CV285952.1	no blast match			0		278
472	CV285953.1	GB|ABA42878.1| small heat shock protein [uncultured cnidarian]	ABA42878.1	2,00E-08	37	33/87	353
473	CV285956.1	REF|YP_776995.1| hypothetical protein Bamb_5112 [Burkholderia ce...	YP_776995.1	2,00E-25	41	69/165	496
474	CV285963.1	no blast match			0		233
475	CV285977.1	no blast match			0		185
476	CV285989.1	GB|AAT00507.1| Kazal-like serine protease inhibitor EPI8 [Phytop...	AAT00507.1	3,00E-07	43	22/51	320
477	CV285995.1	GB|AAD43811.1|AF159157_1 head-activator binding protein precurso...	AAD43811.1	1,00E-74	99	117/118	435
478	CV286004.1	no blast match			0		182
479	CV286005.1	REF|NP_001016677.1| nuclease sensitive element binding protein 1...	NP_001016677.1	3,00E-17	61	46/75	311
480	CV286006.1	REF|XP_001232777.1| PREDICTED: similar to stromelysin-3 [Gallus ...	XP_001232777.1	2,00E-17	53	51/95	287
481	CV286009.1	REF|NP_001050950.1| Os03g0690000 [Oryza sativa (japonica cultiva...	NP_001050950.1	3,00E-16	75	41/54	269
482	CV286012.1	REF|XP_792994.1| PREDICTED: similar to muscle protein 20-like pr...	XP_792994.1	3,00E-11	52	37/70	223
483	CV286014.1	REF|XP_392465.3| PREDICTED: similar to squid CG16901-PC, isoform...	XP_392465.3	1,00E-14	35	55/155	538
484	CV286016.1	REF|XP_391917.2| PREDICTED: similar to Adenosylhomocysteinase at...	XP_391917.2	8,00E-23	67	55/82	247
485	CV286027.1	REF|XP_512771.2| PREDICTED: similar to calmodulin [Pan troglodytes]	XP_512771.2	3,00E-10	88	32/36	152
486	CV286028.1	no blast match			0		186
487	CV286031.1	no blast match			0		614
488	CV286033.1	no blast match			0		189
489	CV286048.1	SP|P26256|ANX12_HYDAT Annexin-B12 (Annexin-12) (Annexin XII) >gi...	P26256	2,00E-49	100	100/100	303
490	CV286050.1	no blast match			0		118
491	CV286051.1	no blast match			0		145
492	CV286052.1	REF|XP_656030.1| diaphanous protein [Entamoeba histolytica HM-1:...	XP_656030.1	4,00E-08	40	36/89	322
493	CV286060.1	DBJ|BAE90280.1| unnamed protein product [Macaca fascicularis]	BAE90280.1	9,00E-35	81	72/88	343
494	CV286065.1	REF|XP_612678.3| PREDICTED: hypothetical protein [Bos taurus]	XP_612678.3	7,00E-06	92	24/26	342
495	CV286066.1	GB|AAW26622.1| SJCHGC01809 protein [Schistosoma japonicum]	AAW26622.1	1,00E-08	41	26/62	199
496	CV286068.1	REF|XP_001373032.1| PREDICTED: hypothetical protein [Monodelphis...	XP_001373032.1	6,00E-25	55	61/109	401
497	CV286070.1	no blast match			0		163
498	CV286071.1	no blast match			0		306
499	CV286074.1	EMB|CAK10829.1| novel protein similar to vertebrate CD151 antige...	CAK10829.1	5,00E-19	38	49/126	383
500	CV286098.1	no blast match			0		307
501	CV286110.1	GB|EAW96596.1| ovochymase 1, isoform CRA_b [Homo sapiens]	EAW96596.1	3,00E-10	47	37/78	253
502	CV286112.1	REF|NP_001069195.1| hypothetical protein LOC515723 [Bos taurus] ...	NP_001069195.1	3,00E-38	48	72/150	487
503	CV286115.1	GB|EAW61158.1| polypyrimidine tract binding protein 1, isoform C...	EAW61158.1	6,00E-21	52	45/86	260
504	CV286120.1	GB|AAA92361.2| metalloproteinase 1 [Hydra vulgaris]	AAA92361.2	8,00E-47	97	89/91	274
505	CV286125.1	GB|ABL67655.1| putative cyclophilin [Citrus cv. Shiranuhi]	ABL67655.1	7,00E-14	72	35/48	161
506	CV286128.1	REF|XP_001353695.1| GA18578-PA [Drosophila pseudoobscura] >gi|54...	XP_001353695.1	1,00E-19	87	48/55	169
507	CV286129.1	no blast match			0		202
508	CV286133.1	no blast match			0		191
509	CV286135.1	no blast match			0		296
510	CV286138.1	no blast match			0		105
511	CV286141.1	no blast match			0		295
512	CV286151.1	no blast match			0		120
513	CV286156.1	no blast match			0		286
