# Supplementary material for: A Novel Gene Family Controls Species-Specific Morphological Traits in Hydra
Source: PLoS Biol. 2008 Nov 18;6(11):e278. doi: 10.1371/journal.pbio.0060278 (PMC2586386; doi:10.1371/journal.pbio.0060278)
Supplement: Table S3 — Results of the BLASTN search of H. oligactis clusters and singletons from the Kiel 6 library against all H. magnipapillata ESTs. The cut-off value was set to E < 1e–10. Sequences with E value ≥ 1e–10 were referred to as having no significant similarity among H. magnipapillata ESTs (transcripts of potential H. oligactis-specific genes). (68 KB PDF) [file pbio.0060278.st003.pdf]

Table S3

Kiel 6 (*Hydra oligactis* specific) SSH library. Results of BLASTN search against *Hydra* ESTs

Results of the BLASTN search of *H.oligactis* clusters and singletons from Kiel 6 library against all *H.magnipapillata ESTs* . The cut-off value was set to E < 1e-10. Sequences with E value ≥ 1e-10 were referred to as having no significant homology among *H.magnipapillata* ESTs (transcripts of potential *Hydra oligactis* -specific genes).

Pct Idnt, % identity between sequence in *H.oligactis* SSH library and best BLASTN match from *H.magnipapillata* ESTs

Name	Best blast match (with E-value < 1e-10)	Accession number	Expect	Pct Idnt
CL9CONTIG1	no blast match			0
CL34CONTIG1	no blast match			0
CL44CONTIG1	no blast match			0
CL47CONTIG1	no blast match			0
CL51CONTIG1	no blast match			0
CL55CONTIG2	no blast match			0
CL58CONTIG1	no blast match			0
CL68CONTIG1	no blast match			0
CL74CONTIG1	no blast match			0
CL78CONTIG1	no blast match			0
CL93CONTIG1	no blast match			0
CL95CONTIG1	no blast match			0
CL98CONTIG1	no blast match			0
CL106CONTIG1	no blast match			0
CL109CONTIG1	no blast match			0
CL112CONTIG1	no blast match			0
CL117CONTIG1	no blast match			0
CL118CONTIG1	no blast match			0
CL137CONTIG1	no blast match			0
CL150CONTIG1	no blast match			0
CL152CONTIG1	no blast match			0
CL159CONTIG1	no blast match			0
CL164CONTIG1	no blast match			0
CL168CONTIG1	no blast match			0
CL170CONTIG1	no blast match			0
CL171CONTIG1	no blast match			0
CL174CONTIG1	no blast match			0
CL179CONTIG1	no blast match			0
CL183CONTIG1	no blast match			0
CL190CONTIG1	no blast match			0
CL191CONTIG1	no blast match			0
CL196CONTIG1	no blast match			0
CL211CONTIG1	no blast match			0
CL223CONTIG1	no blast match			0
CL225CONTIG1	no blast match			0
CL234CONTIG1	no blast match			0
CL240CONTIG1	no blast match			0
CV284328.1	no blast match			0
CV284335.1	no blast match			0
CV284337.1	no blast match			0
CV284359.1	no blast match			0
CV284419.1	no blast match			0
CV284425.1	no blast match			0
CV284430.1	no blast match			0
CV284439.1	no blast match			0
CV284458.1	no blast match			0
CV284539.1	no blast match			0
CV284555.1	no blast match			0
CV284569.1	no blast match			0
CV284636.1	no blast match			0
CV284638.1	no blast match			0
CV284639.1	no blast match			0
CV285062.1	no blast match			0
CV285070.1	no blast match			0
CV285087.1	no blast match			0
CV285128.1	no blast match			0
CV285163.1	no blast match			0
CV285186.1	no blast match			0
CV285197.1	no blast match			0
CV285198.1	no blast match			0
CV285199.1	no blast match			0
CV285216.1	no blast match			0
CV285237.1	no blast match			0
CV285240.1	no blast match			0
CV285249.1	no blast match			0
CV285253.1	no blast match			0
CV285354.1	no blast match			0
CV285358.1	no blast match			0
CV285375.1	no blast match			0
CV285401.1	no blast match			0
CV285404.1	no blast match			0

CV285426.1	no blast match			0
CV285442.1	no blast match			0
CV285449.1	no blast match			0
CV285451.1	no blast match			0
CV285468.1	no blast match			0
CV285528.1	no blast match			0
CV285529.1	no blast match			0
CV285537.1	no blast match			0
CV285581.1	no blast match			0
CV285601.1	no blast match			0
CV285654.1	no blast match			0
CV285673.1	no blast match			0
CV285714.1	no blast match			0
CV285485.1	GB|CN772184.1|CN772184 tad80g01.x1 Hydra EST Darmstadt I Hydra m...	CN772184.1	1,00E-28	80
CL16CONTIG1	GB|CN628929.1|CN628929 taf46h12.x1 Hydra EST Darmstadt I Hydra m...	CN628929.1	7,00E-34	81
CL101CONTIG1	GB|DT617998.1|DT617998 ACAH-aaa33b03.g1 Hydra_EST_UCI-10 Hydra m...	DT617998.1	2,00E-28	81
CL147CONTIG1	GB|CN768700.1|CN768700 taf18c02.x1 Hydra EST Darmstadt I Hydra m...	CN768700.1	3,00E-14	81
CL149CONTIG1	GB|BP505420.1|BP505420 BP505420 Hydra magnipapillata cDNA librar...	BP505420.1	3,00E-18	81
CV285202.1	GB|CD266159.1|CD266159 tab14b07.x1 Hydra EST -III Hydra magnipap...	CD266159.1	1,00E-24	81
CL57CONTIG1	GB|DN137229.2|DN137229 ACAE-aaa06k17.b3 Hydra EST UCI 5 Hydra ma...	DN137229.2	3,00E-36	82
CL162CONTIG1	GB|BP522756.1|BP522756 BP522756 Hydra magnipapillata cDNA librar...	BP522756.1	3,00E-21	82
CV284344.1	GB|CD268186.1|CD268186 tab76g06.x1 Hydra EST -III Hydra magnipap...	CD268186.1	4,00E-25	82
CV285106.1	GB|CO374774.1|CO374774 tah19a04.y2 Hydra EST -Kiel 5 Hydra magni...	CO374774.1	4,00E-44	82
CV285472.1	GB|CN560028.1|CN560028 tag35b08.y1 Hydra EST -Kiel 2 Hydra magni...	CN560028.1	2,00E-26	82
CL20CONTIG1	GB|CV042373.1|CV042373 tai55c03.y2 Hydra EST UCI 5 ALP Hydra mag...	CV042373.1	2,00E-23	83
CL25CONTIG2	GB|BP518180.1|BP518180 BP518180 Hydra magnipapillata cDNA librar...	BP518180.1	4,00E-39	83
CL129CONTIG1	GB|CN551817.1|CN551817 tad78g02.x2 Hydra EST Darmstadt I Hydra m...	CN551817.1	2,00E-46	83
CL210CONTIG1	GB|CB935670.1|CB935670 tab94d08.x1 Hydra EST -III Hydra magnipap...	CB935670.1	3,00E-38	83
CV284362.1	GB|DN243642.2|DN243642 ACAE-aaa46e05.b1 Hydra EST UCI 5 Hydra ma...	DN243642.2	5,00E-59	83
CV284600.1	GB|DN814925.2|DN814925 ACAC-aac44f16.g1 Hydra EST UCI 7 Hydra ma...	DN814925.2	2,00E-19	83
CV285054.1	GB|BP507210.1|BP507210 BP507210 Hydra magnipapillata cDNA librar...	BP507210.1	6,00E-55	83
CV285458.1	GB|CN625099.1|CN625099 tae66a02.y1 Hydra EST Darmstadt I Hydra m...	CN625099.1	1,00E-15	83
CL25CONTIG1	GB|BP518180.1|BP518180 BP518180 Hydra magnipapillata cDNA librar...	BP518180.1	2,00E-26	84
CL30CONTIG1	GB|CV223226.1|CV223226 taj59b11.y1 Hydra EST UCI 6 Hydra magnipa...	CV223226.1	2,00E-36	84
CL31CONTIG1	GB|BP505951.1|BP505951 BP505951 Hydra magnipapillata cDNA librar...	BP505951.1	2,00E-51	84
CL70CONTIG1	GB|DT616761.1|DT616761 ACAH-aaa90b08.g1 Hydra_EST_UCI-10 Hydra m...	DT616761.1	2,00E-32	84
CL102CONTIG1	GB|CD566381.1|CD566381 tac04f05.x1 Hydra EST -III Hydra magnipap...	CD566381.1	5,00E-16	84
CL124CONTIG1	GB|CN628043.1|CN628043 tae87d09.y1 Hydra EST Darmstadt I Hydra m...	CN628043.1	3,00E-23	84
CL187CONTIG1	GB|CF778597.1|CF778597 tad19h03.x1 Hydra EST -IV Hydra magnipapi...	CF778597.1	4,00E-43	84
CL213CONTIG1	GB|CN631613.1|CN631613 taf42c02.y1 Hydra EST Darmstadt I Hydra m...	CN631613.1	2,00E-37	84
CL217CONTIG1	GB|CN774468.1|CN774468 tae81f08.x1 Hydra EST Darmstadt I Hydra m...	CN774468.1	1,00E-16	84
CV284397.1	GB|DT617287.1|DT617287 ACAH-aaa48g12.g1 Hydra_EST_UCI-10 Hydra m...	DT617287.1	6,00E-77	84
CV284607.1	GB|CO376392.1|CO376392 tah34d11.y1 Hydra EST -Kiel 5 Hydra magni...	CO376392.1	4,00E-40	84
CV285200.1	GB|CB889894.1|CB889894 taa62h04.x1 Hydra EST -III Hydra magnipap...	CB889894.1	1,00E-11	84
CL18CONTIG1	GB|CN552883.1|CN552883 tae41f09.y1 Hydra EST Darmstadt I Hydra m...	CN552883.1	4,00E-84	85
CL18CONTIG2	GB|CN552883.1|CN552883 tae41f09.y1 Hydra EST Darmstadt I Hydra m...	CN552883.1	2,00E-12	85
CL22CONTIG1	GB|CB888887.1|CB888887 taa43b09.x3 Hydra EST -III Hydra magnipap...	CB888887.1	4,00E-29	85
CL42CONTIG1	GB|CB888788.1|CB888788 taa41f05.x3 Hydra EST -III Hydra magnipap...	CB888788.1	1,00E-25	85
CL55CONTIG1	GB|CF655458.1|CF655458 tac89f12.y1 Hydra EST -IV Hydra magnipapi...	CF655458.1	2,00E-15	85
CL71CONTIG1	GB|CB271376.1|CB271376 taa22d03.x2 Hydra EST -II Hydra magnipapi...	CB271376.1	9,00E-35	85
CL81CONTIG1	GB|CD285656.1|CD285656 tab26d11.x1 Hydra EST -III Hydra magnipap...	CD285656.1	1,00E-19	85
CL105CONTIG1	GB|CD567489.1|CD567489 tab78c01.x1 Hydra EST -III Hydra magnipap...	CD567489.1	4,00E-75	85
CL110CONTIG1	GB|CA302256.1|CA302256 taa12d01.y1 Hydra cDNA library Hydra magn...	CA302256.1	3,00E-67	85
CL130CONTIG1	GB|CN633559.1|CN633559 taf12d03.y1 Hydra EST Darmstadt I Hydra m...	CN633559.1	4,00E-31	85
CL142CONTIG1	GB|CO372020.1|CO372020 tah15h01.x2 Hydra EST -Kiel 5 Hydra magni...	CO372020.1	1,00E-124	85
CL193CONTIG1	GB|CD268370.1|CD268370 tab83g12.x1 Hydra EST -III Hydra magnipap...	CD268370.1	2,00E-25	85
CV284413.1	GB|CD267301.1|CD267301 tab71h04.x1 Hydra EST -III Hydra magnipap...	CD267301.1	4,00E-53	85
CV284495.1	GB|CA301617.1|CA301617 taa05f09.y1 Hydra cDNA library Hydra magn...	CA301617.1	2,00E-45	85
CV284543.1	GB|BP518093.1|BP518093 BP518093 Hydra magnipapillata cDNA librar...	BP518093.1	1,00E-74	85
CV284552.1	GB|BP512568.1|BP512568 BP512568 Hydra magnipapillata cDNA librar...	BP512568.1	7,00E-67	85
CV285099.1	GB|CB888772.1|CB888772 taa41d04.x3 Hydra EST -III Hydra magnipap...	CB888772.1	3,00E-14	85
CV285147.1	GB|CN628311.1|CN628311 tae94e07.x1 Hydra EST Darmstadt I Hydra m...	CN628311.1	2,00E-36	85
CV285179.1	GB|CB073137.1|CB073137 taa28g09.y1 Hydra EST -II Hydra magnipapi...	CB073137.1	3,00E-69	85
CV285225.1	GB|BP509924.1|BP509924 BP509924 Hydra magnipapillata cDNA librar...	BP509924.1	2,00E-93	85
CV285255.1	GB|BP514276.1|BP514276 BP514276 Hydra magnipapillata cDNA librar...	BP514276.1	2,00E-36	85
CV285344.1	GB|CX054754.1|CX054754 taj05e02.x2 Hydra EST UCI 5 ALP Hydra mag...	CX054754.1	9,00E-68	85
CV285416.1	GB|BP517561.1|BP517561 BP517561 Hydra magnipapillata cDNA librar...	BP517561.1	7,00E-67	85
CV285448.1	GB|DT607170.1|DT607170 ACAG-aaa53h04.g1 Hydra_EST_UCI-9 Hydra ma...	DT607170.1	3,00E-98	85
CV285636.1	GB|BP510951.1|BP510951 BP510951 Hydra magnipapillata cDNA librar...	BP510951.1	5,00E-26	85
CL59CONTIG1	GB|CN552569.1|CN552569 tae20h09.y1 Hydra EST Darmstadt I Hydra m...	CN552569.1	1,00E-77	86
CL62CONTIG1	GB|CN552014.1|CN552014 tae18d06.x1 Hydra EST Darmstadt I Hydra m...	CN552014.1	3,00E-54	86
CL67CONTIG1	GB|CF653876.1|CF653876 tac77e07.y1 Hydra EST -IV Hydra magnipapi...	CF653876.1	1,00E-36	86
CL116CONTIG1	GB|BP507389.1|BP507389 BP507389 Hydra magnipapillata cDNA librar...	BP507389.1	1,00E-84	86
CL133CONTIG1	GB|CA301714.1|CA301714 taa07a10.y1 Hydra cDNA library Hydra magn...	CA301714.1	8,00E-33	86
CL135CONTIG1	GB|BP522679.1|BP522679 BP522679 Hydra magnipapillata cDNA librar...	BP522679.1	3,00E-21	86
CL178CONTIG1	GB|CD567717.1|CD567717 tab81c08.x1 Hydra EST -III Hydra magnipap...	CD567717.1	6,00E-34	86
CV284326.1	GB|CX833966.2|CX833966 ACAC-aaa64g06.g1 Hydra EST UCI 7 Hydra ma...	CX833966.2	1,00E-87	86
CV284349.1	GB|CB271348.1|CB271348 taa21g08.x2 Hydra EST -II Hydra magnipapi...	CB271348.1	1,00E-112	86
CV284427.1	GB|DN244722.2|DN244722 ACAE-aaa46m03.b1 Hydra EST UCI 5 Hydra ma...	DN244722.2	1,00E-31	86
CV284512.1	GB|CV863275.2|CV863275 ACAB-aaa09c07.g1 Hydra UCI6- barcoded EST...	CV863275.2	8,00E-70	86
CV284532.1	GB|CN552516.1|CN552516 tae20c11.y1 Hydra EST Darmstadt I Hydra m...	CN552516.1	9,00E-63	86
CV284599.1	GB|CN561990.1|CN561990 tag27g01.x1 Hydra EST -Kiel 2 Hydra magni...	CN561990.1	6,00E-64	86
CV285057.1	GB|DN242121.2|DN242121 ACAD-aaa14c16.g1 Hydra_EST_UCI-8 Hydra ma...	DN242121.2	4,00E-65	86

CV285094.1	GB|BP505262.1|BP505262 BP505262 Hydra magnipapillata cDNA librar...	BP505262.1	3,00E-69	86
CV285126.1	GB|CN551939.1|CN551939 tae17e08.x1 Hydra EST Darmstadt I Hydra m...	CN551939.1	3,00E-70	86
CV285138.1	GB|CB888510.1|CB888510 taa77e06.x1 Hydra EST -III Hydra magnipap...	CB888510.1	5,00E-44	86
CV285165.1	GB|BP504763.1|BP504763 BP504763 Hydra magnipapillata cDNA librar...	BP504763.1	3,00E-21	86
CV285213.1	GB|CV184773.1|CV184773 taj06b02.y1 Hydra EST UCI 5 ALP Hydra mag...	CV184773.1	3,00E-63	86
CV285222.1	GB|CA302368.1|CA302368 taa14a11.y1 Hydra cDNA library Hydra magn...	CA302368.1	2,00E-64	86
CV285411.1	GB|CB889053.1|CB889053 taa45e11.x1 Hydra EST -III Hydra magnipap...	CB889053.1	2,00E-54	86
CV285632.1	GB|DR435210.1|DR435210 ACAB-aaa27a08.g1 Hydra UCI6- barcoded EST...	DR435210.1	4,00E-22	86
CL5CONTIG1	GB|CN623264.1|CN623264 tae08h09.x1 Hydra EST Darmstadt I Hydra m...	CN623264.1	1,00E-28	87
CL26CONTIG1	GB|CA302757.1|CA302757 taa01c04.x1 Hydra cDNA library Hydra magn...	CA302757.1	1,00E-111	87
CL27CONTIG1	GB|BP511210.1|BP511210 BP511210 Hydra magnipapillata cDNA librar...	BP511210.1	2,00E-64	87
CL28CONTIG1	GB|CF656681.1|CF656681 tac67b05.x1 Hydra EST -IV Hydra magnipapi...	CF656681.1	2,00E-89	87
CL82CONTIG1	GB|BP509924.1|BP509924 BP509924 Hydra magnipapillata cDNA librar...	BP509924.1	2,00E-43	87
CL86CONTIG1	GB|CV863794.2|CV863794 ACAB-aaa12d05.g1 Hydra UCI6- barcoded EST...	CV863794.2	1,00E-48	87
CL90CONTIG1	GB|CX834146.2|CX834146 ACAC-aaa77f03.g1 Hydra EST UCI 7 Hydra ma...	CX834146.2	6,00E-39	87
CL108CONTIG1	GB|DN636637.2|DN636637 ACAC-aab47n19.g1 Hydra EST UCI 7 Hydra ma...	DN636637.2	3,00E-88	87
CL140CONTIG1	GB|CB888945.1|CB888945 taa44a07.x3 Hydra EST -III Hydra magnipap...	CB888945.1	9,00E-66	87
CL145CONTIG1	GB|CA302180.1|CA302180 taa11c04.y1 Hydra cDNA library Hydra magn...	CA302180.1	9,00E-40	87
CL169CONTIG1	GB|BP515977.1|BP515977 BP515977 Hydra magnipapillata cDNA librar...	BP515977.1	4,00E-42	87
CL180CONTIG1	GB|CN772532.1|CN772532 tad82h02.y1 Hydra EST Darmstadt I Hydra m...	CN772532.1	2,00E-73	87
CL182CONTIG1	GB|CD567323.1|CD567323 tab51c12.x1 Hydra EST -III Hydra magnipap...	CD567323.1	4,00E-28	87
CL189CONTIG1	GB|CB887977.1|CB887977 taa85e09.x1 Hydra EST -III Hydra magnipap...	CB887977.1	8,00E-52	87
CL201CONTIG1	GB|DR435252.1|DR435252 ACAB-aaa27e08.g1 Hydra UCI6- barcoded EST...	DR435252.1	1,00E-106	87
CL204CONTIG1	GB|CO374411.1|CO374411 tah16c05.x2 Hydra EST -Kiel 5 Hydra magni...	CO374411.1	3,00E-81	87
CL218CONTIG1	GB|DN137134.2|DN137134 ACAB-aaa81n04.g1 Hydra UCI6- barcoded EST...	DN137134.2	2,00E-64	87
CL229CONTIG1	GB|DN138057.2|DN138057 ACAE-aaa12d01.b3 Hydra EST UCI 5 Hydra ma...	DN138057.2	3,00E-38	87
CL233CONTIG1	GB|CX771156.2|CX771156 ACAD-aab35j07.b1 Hydra_EST_UCI-8 Hydra ma...	CX771156.2	9,00E-47	87
CV284408.1	GB|CN626380.1|CN626380 tae98a10.x1 Hydra EST Darmstadt I Hydra m...	CN626380.1	1,00E-37	87
CV284455.1	GB|CA302201.1|CA302201 taa11e05.y1 Hydra cDNA library Hydra magn...	CA302201.1	7,00E-73	87
CV284548.1	GB|CN552370.1|CN552370 tae18f04.y1 Hydra EST Darmstadt I Hydra m...	CN552370.1	1,00E-106	87
CV285129.1	GB|CB889628.1|CB889628 taa39b06.x3 Hydra EST -III Hydra magnipap...	CB889628.1	1,00E-55	87
CV285148.1	GB|CN556658.1|CN556658 tae39d12.x1 Hydra EST Darmstadt I Hydra m...	CN556658.1	2,00E-28	87
CV285229.1	GB|CV514446.1|CV514446 taj61b03.x2 Hydra EST UCI 6 Hydra magnipa...	CV514446.1	2,00E-48	87
CV285463.1	GB|BP506085.1|BP506085 BP506085 Hydra magnipapillata cDNA librar...	BP506085.1	1,00E-33	87
CV285551.1	GB|BP518451.1|BP518451 BP518451 Hydra magnipapillata cDNA librar...	BP518451.1	7,00E-11	87
CV285642.1	GB|CF656881.1|CF656881 tac69h10.x1 Hydra EST -IV Hydra magnipapi...	CF656881.1	1,00E-143	87
CL4CONTIG1	GB|BP509943.1|BP509943 BP509943 Hydra magnipapillata cDNA librar...	BP509943.1	3,00E-47	88
CL13CONTIG1	GB|BP514687.1|BP514687 BP514687 Hydra magnipapillata cDNA librar...	BP514687.1	4,00E-67	88
CL21CONTIG1	GB|CB073911.1|CB073911 taa21h09.y1 Hydra EST -II Hydra magnipapi...	CB073911.1	7,00E-76	88
CL38CONTIG1	GB|BP505453.1|BP505453 BP505453 Hydra magnipapillata cDNA librar...	BP505453.1	1,00E-115	88
CL65CONTIG1	GB|CB889122.1|CB889122 taa46f09.x1 Hydra EST -III Hydra magnipap...	CB889122.1	2,00E-54	88
CL75CONTIG1	GB|CF601357.1|CF601357 tac36c08.y1 Hydra EST -IV Hydra magnipapi...	CF601357.1	4,00E-58	88
CL91CONTIG1	GB|DT610598.1|DT610598 ACAG-aab13d12.g1 Hydra_EST_UCI-9 Hydra ma...	DT610598.1	2,00E-72	88
CL104CONTIG1	GB|DN813419.2|DN813419 ACAC-aac49c09.g1 Hydra EST UCI 7 Hydra ma...	DN813419.2	1,00E-103	88
CL113CONTIG1	GB|CF674092.1|CF674092 tad01h11.x1 Hydra EST -IV Hydra magnipapi...	CF674092.1	8,00E-41	88
CL123CONTIG1	GB|BP516354.1|BP516354 BP516354 Hydra magnipapillata cDNA librar...	BP516354.1	1,00E-165	88
CL125CONTIG1	GB|CN554301.1|CN554301 tae35d05.y1 Hydra EST Darmstadt I Hydra m...	CN554301.1	6,00E-33	88
CL126CONTIG1	GB|CA301791.1|CA301791 taa08b03.y1 Hydra cDNA library Hydra magn...	CA301791.1	2,00E-42	88
CL127CONTIG1	GB|BP506640.1|BP506640 BP506640 Hydra magnipapillata cDNA librar...	BP506640.1	2,00E-70	88
CL172CONTIG1	GB|CN551870.1|CN551870 tad79f09.x2 Hydra EST Darmstadt I Hydra m...	CN551870.1	1,00E-86	88
CL185CONTIG1	GB|CA303257.1|CA303257 taa05g04.x1 Hydra cDNA library Hydra magn...	CA303257.1	2,00E-54	88
CL188CONTIG1	GB|CD266535.1|CD266535 tab19g05.x1 Hydra EST -III Hydra magnipap...	CD266535.1	1,00E-55	88
CL207CONTIG1	GB|CF674033.1|CF674033 tad01a06.x1 Hydra EST -IV Hydra magnipapi...	CF674033.1	6,00E-13	88
CL231CONTIG1	GB|CB073712.1|CB073712 taa20c08.y1 Hydra EST -II Hydra magnipapi...	CB073712.1	1,00E-15	88
CL232CONTIG1	GB|CN624739.1|CN624739 tae65d12.x1 Hydra EST Darmstadt I Hydra m...	CN624739.1	1,00E-42	88
CL237CONTIG1	GB|CB272074.1|CB272074 taa30e07.x2 Hydra EST -II Hydra magnipapi...	CB272074.1	1,00E-21	88
CL239CONTIG1	GB|CN774472.1|CN774472 tae81g01.x1 Hydra EST Darmstadt I Hydra m...	CN774472.1	3,00E-23	88
CV284394.1	GB|CD567720.1|CD567720 tab81c11.x1 Hydra EST -III Hydra magnipap...	CD567720.1	4,00E-81	88
CV284409.1	GB|CB888612.1|CB888612 taa79a10.x1 Hydra EST -III Hydra magnipap...	CB888612.1	6,00E-55	88
CV284438.1	GB|CF653876.1|CF653876 tac77e07.y1 Hydra EST -IV Hydra magnipapi...	CF653876.1	1,00E-77	88
CV284480.1	GB|CF778958.1|CF778958 tad24h07.x1 Hydra EST -IV Hydra magnipapi...	CF778958.1	7,00E-42	88
CV284585.1	GB|CF654579.1|CF654579 tac83d09.y1 Hydra EST -IV Hydra magnipapi...	CF654579.1	1,00E-165	88
CV285078.1	GB|CA302244.1|CA302244 taa12b06.y1 Hydra cDNA library Hydra magn...	CA302244.1	4,00E-17	88
CV285108.1	GB|CN552933.1|CN552933 tae42c02.y1 Hydra EST Darmstadt I Hydra m...	CN552933.1	1,00E-105	88
CV285137.1	GB|BP504854.1|BP504854 BP504854 Hydra magnipapillata cDNA librar...	BP504854.1	1,00E-133	88
CV285309.1	GB|CO537847.1|CO537847 tah79d03.y1 Hydra EST UCI 5 Hydra magnipa...	CO537847.1	1,00E-74	88
CV285348.1	GB|CN552100.1|CN552100 tae19d10.x1 Hydra EST Darmstadt I Hydra m...	CN552100.1	1,00E-32	88
CV285701.1	GB|CO374929.1|CO374929 tah25c05.x1 Hydra EST -Kiel 5 Hydra magni...	CO374929.1	2,00E-20	88
CL19CONTIG1	GB|CN625966.1|CN625966 tae53c12.y1 Hydra EST Darmstadt I Hydra m...	CN625966.1	4,00E-77	89
CL35CONTIG1	GB|CD268002.1|CD268002 tab13f10.x1 Hydra EST -III Hydra magnipap...	CD268002.1	7,00E-60	89
CL63CONTIG1	GB|CB889756.1|CB889756 taa40h03.x3 Hydra EST -III Hydra magnipap...	CB889756.1	1,00E-36	89
CL64CONTIG1	GB|CA302441.1|CA302441 taa15a10.y1 Hydra cDNA library Hydra magn...	CA302441.1	2,00E-69	89
CL96CONTIG1	GB|BP508041.1|BP508041 BP508041 Hydra magnipapillata cDNA librar...	BP508041.1	1,00E-115	89
CL99CONTIG1	GB|CO371949.1|CO371949 tah14g10.x2 Hydra EST -Kiel 5 Hydra magni...	CO371949.1	1,00E-138	89
CL131CONTIG1	GB|CN628840.1|CN628840 tae96f11.y1 Hydra EST Darmstadt I Hydra m...	CN628840.1	1,00E-174	89
CL138CONTIG1	GB|CX832420.2|CX832420 ACAC-aaa35f03.g1 Hydra EST UCI 7 Hydra ma...	CX832420.2	6,00E-51	89
CL144CONTIG1	GB|CA302769.1|CA302769 taa01e03.x1 Hydra cDNA library Hydra magn...	CA302769.1	1,00E-133	89
CL151CONTIG1	GB|BP506847.1|BP506847 BP506847 Hydra magnipapillata cDNA librar...	BP506847.1	3,00E-43	89
CL153CONTIG1	GB|BP508518.1|BP508518 BP508518 Hydra magnipapillata cDNA librar...	BP508518.1	4,00E-69	89
CL154CONTIG1	GB|CN772339.1|CN772339 tad80c06.y1 Hydra EST Darmstadt I Hydra m...	CN772339.1	4,00E-99	89
CL203CONTIG1	GB|CA302481.1|CA302481 taa15f07.y1 Hydra cDNA library Hydra magn...	CA302481.1	4,00E-97	89
CL219CONTIG1	GB|CF653876.1|CF653876 tac77e07.y1 Hydra EST -IV Hydra magnipapi...	CF653876.1	1,00E-70	89
CL238CONTIG1	GB|CN551548.1|CN551548 tad74c11.x2 Hydra EST Darmstadt I Hydra m...	CN551548.1	2,00E-32	89

CV284329.1	GB|CO537307.1|CO537307 tah69e11.x1 Hydra EST UCI 5 Hydra magnipa...	CO537307.1	2,00E-40	89
CV284347.1	GB|DN811598.2|DN811598 ACAC-aab42g04.g1 Hydra EST UCI 7 Hydra ma...	DN811598.2	1,00E-101	89
CV284358.1	GB|CD268647.1|CD268647 taa98a07.x1 Hydra EST -III Hydra magnipap...	CD268647.1	1,00E-77	89
CV284360.1	GB|BP519387.1|BP519387 BP519387 Hydra magnipapillata cDNA librar...	BP519387.1	2,00E-98	89
CV284414.1	GB|CB271664.1|CB271664 taa20b01.x2 Hydra EST -II Hydra magnipapi...	CB271664.1	1,00E-119	89
CV284474.1	GB|CB888747.1|CB888747 taa41a08.x3 Hydra EST -III Hydra magnipap...	CB888747.1	6,00E-45	89
CV284502.1	GB|CB073950.1|CB073950 taa22e10.y1 Hydra EST -II Hydra magnipapi...	CB073950.1	4,00E-38	89
CV284508.1	GB|CO745725.1|CO745725 tah87h10.x1 Hydra EST UCI 5 Hydra magnipa...	CO745725.1	1,00E-104	89
CV284521.1	GB|CF657943.1|CF657943 tac78b08.x1 Hydra EST -IV Hydra magnipapi...	CF657943.1	9,00E-51	89
CV284553.1	GB|CD680270.1|CD680270 tac20a08.y1 Hydra EST -IV Hydra magnipapi...	CD680270.1	4,00E-78	89
CV285111.1	GB|CN626657.1|CN626657 tae98b05.y1 Hydra EST Darmstadt I Hydra m...	CN626657.1	6,00E-59	89
CV285146.1	GB|CN559738.1|CN559738 tag30h08.y1 Hydra EST -Kiel 2 Hydra magni...	CN559738.1	4,00E-39	89
CV285239.1	GB|CB888710.1|CB888710 taa80e07.x1 Hydra EST -III Hydra magnipap...	CB888710.1	2,00E-85	89
CV285268.1	GB|CF654214.1|CF654214 tac82a01.x1 Hydra EST -IV Hydra magnipapi...	CF654214.1	1,00E-123	89
CV285313.1	GB|CA302984.1|CA302984 taa01f02.y1 Hydra cDNA library Hydra magn...	CA302984.1	4,00E-87	89
CV285486.1	GB|CA302981.1|CA302981 taa01e11.y1 Hydra cDNA library Hydra magn...	CA302981.1	1,00E-104	89
CV285522.1	GB|CB888652.1|CB888652 taa79f11.x1 Hydra EST -III Hydra magnipap...	CB888652.1	1,00E-54	89
CV285629.1	GB|DN603585.2|DN603585 ACAC-aac12p24.g1 Hydra EST UCI 7 Hydra ma...	DN603585.2	1,00E-79	89
CV285644.1	GB|CN551380.1|CN551380 tad72a06.x2 Hydra EST Darmstadt I Hydra m...	CN551380.1	8,00E-13	89
CV285713.1	GB|CF655237.1|CF655237 tac86g03.y1 Hydra EST -IV Hydra magnipapi...	CF655237.1	2,00E-88	89
CL8CONTIG1	GB|CA301602.1|CA301602 taa05e01.y1 Hydra cDNA library Hydra magn...	CA301602.1	1,00E-120	90
CL15CONTIG1	GB|CF777577.1|CF777577 tad12g02.x1 Hydra EST -IV Hydra magnipapi...	CF777577.1	0	90
CL33CONTIG1	GB|CV185198.1|CV185198 taj10b03.y1 Hydra EST UCI 5 ALP Hydra mag...	CV185198.1	0	90
CL36CONTIG1	GB|CA302368.1|CA302368 taa14a11.y1 Hydra cDNA library Hydra magn...	CA302368.1	1,00E-100	90
CL43CONTIG1	GB|CA303111.1|CA303111 taa03d01.y1 Hydra cDNA library Hydra magn...	CA303111.1	1,00E-139	90
CL48CONTIG1	GB|CN631025.1|CN631025 taf50e07.y1 Hydra EST Darmstadt I Hydra m...	CN631025.1	3,00E-54	90
CL61CONTIG1	GB|BP516406.1|BP516406 BP516406 Hydra magnipapillata cDNA librar...	BP516406.1	3,00E-81	90
CL72CONTIG1	GB|CA301700.1|CA301700 taa06h02.y1 Hydra cDNA library Hydra magn...	CA301700.1	1,00E-99	90
CL83CONTIG1	GB|CV463900.1|CV463900 taj32g02.y1 Hydra EST UCI 5 ALP Hydra mag...	CV463900.1	4,00E-77	90
CL84CONTIG1	GB|CN552824.1|CN552824 tae41a05.y1 Hydra EST Darmstadt I Hydra m...	CN552824.1	1,00E-108	90
CL97CONTIG1	GB|CD285672.1|CD285672 tab26f09.x1 Hydra EST -III Hydra magnipap...	CD285672.1	1,00E-121	90
CL103CONTIG1	GB|CN623355.1|CN623355 tae06a07.y1 Hydra EST Darmstadt I Hydra m...	CN623355.1	2,00E-12	90
CL111CONTIG1	GB|CD268634.1|CD268634 taa96h05.x1 Hydra EST -III Hydra magnipap...	CD268634.1	1,00E-149	90
CL134CONTIG1	GB|CA302600.1|CA302600 taa13e05.x1 Hydra cDNA library Hydra magn...	CA302600.1	1,00E-115	90
CL139CONTIG1	GB|DN602704.2|DN602704 ACAB-aab00b07.g1 Hydra UCI6- barcoded EST...	DN602704.2	1,00E-168	90
CL161CONTIG1	GB|DN604257.2|DN604257 ACAC-aab80n10.g1 Hydra EST UCI 7 Hydra ma...	DN604257.2	0	90
CL166CONTIG1	GB|CA301640.1|CA301640 taa06a10.y1 Hydra cDNA library Hydra magn...	CA301640.1	1,00E-41	90
CL167CONTIG1	GB|CB073034.1|CB073034 taa27c06.y1 Hydra EST -II Hydra magnipapi...	CB073034.1	6,00E-54	90
CL173CONTIG1	GB|BP512038.1|BP512038 BP512038 Hydra magnipapillata cDNA librar...	BP512038.1	2,00E-27	90
CL176CONTIG1	GB|CD606933.1|CD606933 tac22c05.x1 Hydra EST -IV Hydra magnipapi...	CD606933.1	9,00E-72	90
CL192CONTIG1	GB|CX832124.2|CX832124 ACAC-aaa49a03.g1 Hydra EST UCI 7 Hydra ma...	CX832124.2	1,00E-111	90
CL197CONTIG1	GB|DR435312.1|DR435312 ACAB-aaa28e01.g1 Hydra UCI6- barcoded EST...	DR435312.1	9,00E-63	90
CL200CONTIG1	GB|CV514347.1|CV514347 taj58a08.x2 Hydra EST UCI 6 Hydra magnipa...	CV514347.1	1,00E-123	90
CL214CONTIG1	GB|CN774120.1|CN774120 taf58e03.y2 Hydra EST Darmstadt I Hydra m...	CN774120.1	1,00E-15	90
CV284342.1	GB|CX832269.2|CX832269 ACAC-aaa17e04.g1 Hydra EST UCI 7 Hydra ma...	CX832269.2	7,00E-45	90
CV284356.1	GB|CA301883.1|CA301883 taa09e11.x1 Hydra cDNA library Hydra magn...	CA301883.1	1,00E-117	90
CV284370.1	GB|CN552624.1|CN552624 tae41e10.x1 Hydra EST Darmstadt I Hydra m...	CN552624.1	1,00E-26	90
CV284385.1	GB|BP508644.1|BP508644 BP508644 Hydra magnipapillata cDNA librar...	BP508644.1	2,00E-47	90
CV284396.1	GB|CA302368.1|CA302368 taa14a11.y1 Hydra cDNA library Hydra magn...	CA302368.1	2,00E-33	90
CV284470.1	GB|BP510630.1|BP510630 BP510630 Hydra magnipapillata cDNA librar...	BP510630.1	6,00E-73	90
CV284488.1	GB|CV659889.1|CV659889 tak19g10.y1 Hydra EST UCI 6 Hydra magnipa...	CV659889.1	3,00E-60	90
CV284597.1	GB|CV565070.1|CV565070 taj71b10.y1 Hydra EST UCI 6 Hydra magnipa...	CV565070.1	1,00E-43	90
CV285092.1	GB|CN768851.1|CN768851 taf20b08.x1 Hydra EST Darmstadt I Hydra m...	CN768851.1	4,00E-90	90
CV285173.1	GB|CB888492.1|CB888492 taa77c02.x1 Hydra EST -III Hydra magnipap...	CB888492.1	7,00E-44	90
CV285187.1	GB|CN630900.1|CN630900 taf53a02.x1 Hydra EST Darmstadt I Hydra m...	CN630900.1	2,00E-26	90
CV285242.1	GB|BP508809.1|BP508809 BP508809 Hydra magnipapillata cDNA librar...	BP508809.1	1,00E-74	90
CV285419.1	GB|CF657773.1|CF657773 tac75f06.x1 Hydra EST -IV Hydra magnipapi...	CF657773.1	2,00E-57	90
CV285462.1	GB|CN558940.1|CN558940 tad77h06.y1 Hydra EST Darmstadt I Hydra m...	CN558940.1	1,00E-131	90
CV285523.1	GB|CB271959.1|CB271959 taa28f09.x3 Hydra EST -II Hydra magnipapi...	CB271959.1	6,00E-70	90
CV285594.1	GB|CN632490.1|CN632490 taf02c09.x1 Hydra EST Darmstadt I Hydra m...	CN632490.1	0	90
CV285653.1	GB|CF655927.1|CF655927 tac49d11.y1 Hydra EST -IV Hydra magnipapi...	CF655927.1	1,00E-154	90
CV285684.1	GB|BP517656.1|BP517656 BP517656 Hydra magnipapillata cDNA librar...	BP517656.1	1,00E-142	90
CL1CONTIG1	GB|CD266973.1|CD266973 tab06e10.x1 Hydra EST -III Hydra magnipap...	CD266973.1	1,00E-172	91
CL2CONTIG1	GB|CN622838.1|CN622838 tad96c06.x1 Hydra EST Darmstadt I Hydra m...	CN622838.1	2,00E-46	91
CL37CONTIG1	GB|DT617203.1|DT617203 ACAH-aaa47e03.g1 Hydra_EST_UCI-10 Hydra m...	DT617203.1	1,00E-95	91
CL52CONTIG1	GB|CB073034.1|CB073034 taa27c06.y1 Hydra EST -II Hydra magnipapi...	CB073034.1	6,00E-73	91
CL54CONTIG1	GB|CD567616.1|CD567616 tab79h05.x1 Hydra EST -III Hydra magnipap...	CD567616.1	1,00E-148	91
CL89CONTIG1	GB|CB888636.1|CB888636 taa79d12.x1 Hydra EST -III Hydra magnipap...	CB888636.1	2,00E-80	91
CL119CONTIG1	GB|CN553590.1|CN553590 tae26d09.y1 Hydra EST Darmstadt I Hydra m...	CN553590.1	1,00E-62	91
CL121CONTIG1	GB|BP510682.1|BP510682 BP510682 Hydra magnipapillata cDNA librar...	BP510682.1	1,00E-131	91
CL156CONTIG1	GB|CV181119.1|CV181119 tai73c08.x1 Hydra EST UCI 5 ALP Hydra mag...	CV181119.1	0	91
CL215CONTIG1	GB|CB888166.1|CB888166 taa88c07.x1 Hydra EST -III Hydra magnipap...	CB888166.1	5,00E-52	91
CV284316.1	GB|CN556646.1|CN556646 tae39c05.x1 Hydra EST Darmstadt I Hydra m...	CN556646.1	1,00E-133	91
CV284323.1	GB|CX830338.2|CX830338 ACAC-aaa23b01.g1 Hydra EST UCI 7 Hydra ma...	CX830338.2	5,00E-73	91
CV284324.1	GB|BP506492.1|BP506492 BP506492 Hydra magnipapillata cDNA librar...	BP506492.1	5,00E-80	91
CV284353.1	GB|CB889824.1|CB889824 taa61h04.x1 Hydra EST -III Hydra magnipap...	CB889824.1	8,00E-86	91
CV284453.1	GB|CV184859.1|CV184859 taj07e09.y1 Hydra EST UCI 5 ALP Hydra mag...	CV184859.1	1,00E-176	91
CV284464.1	GB|DN242497.2|DN242497 ACAD-aaa62o18.g1 Hydra_EST_UCI-8 Hydra ma...	DN242497.2	1,00E-157	91
CV284487.1	GB|CN772064.1|CN772064 tad94h02.y1 Hydra EST Darmstadt I Hydra m...	CN772064.1	1,00E-101	91
CV284523.1	GB|CN552141.1|CN552141 tae20a03.x1 Hydra EST Darmstadt I Hydra m...	CN552141.1	1,00E-30	91
CV284537.1	GB|CV514820.1|CV514820 taj63h04.x2 Hydra EST UCI 6 Hydra magnipa...	CV514820.1	7,00E-69	91
CV284566.1	GB|BP516806.1|BP516806 BP516806 Hydra magnipapillata cDNA librar...	BP516806.1	2,00E-22	91
CV285050.1	GB|CN777769.1|CN777769 tad84b04.y2 Hydra EST Darmstadt I Hydra m...	CN777769.1	1,00E-119	91

CV285066.1	GB|DT610319.1|DT610319 ACAG-aaa49c06.g1 Hydra_EST_UCI-9 Hydra ma...	DT610319.1	1,00E-167	91
CV285084.1	GB|CA302164.1|CA302164 taa11a11.y1 Hydra cDNA library Hydra magn...	CA302164.1	5,00E-67	91
CV285251.1	GB|CO509638.1|CO509638 tai57b10.x1 Hydra EST UCI 5 ALP Hydra mag...	CO509638.1	9,00E-72	91
CV285264.1	GB|CO374409.1|CO374409 tah16b12.x2 Hydra EST -Kiel 5 Hydra magni...	CO374409.1	1,00E-22	91
CV285266.1	GB|BP518675.1|BP518675 BP518675 Hydra magnipapillata cDNA librar...	BP518675.1	4,00E-59	91
CV285270.1	GB|DN245619.2|DN245619 ACAE-aaa28l03.b1 Hydra EST UCI 5 Hydra ma...	DN245619.2	1,00E-106	91
CV285371.1	GB|CD566338.1|CD566338 tac04a08.x1 Hydra EST -III Hydra magnipap...	CD566338.1	1,00E-38	91
CV285502.1	GB|CO376150.1|CO376150 tah35b10.x1 Hydra EST -Kiel 5 Hydra magni...	CO376150.1	2,00E-13	91
CV285507.1	GB|CV514602.1|CV514602 taj67h12.y1 Hydra EST UCI 6 Hydra magnipa...	CV514602.1	1,00E-110	91
CV285546.1	GB|CD680489.1|CD680489 tab54a01.x1 Hydra EST -III Hydra magnipap...	CD680489.1	5,00E-75	91
CV285722.1	GB|CB272068.1|CB272068 taa30d10.x2 Hydra EST -II Hydra magnipapi...	CB272068.1	6,00E-73	91
CL6CONTIG1	GB|BP509264.1|BP509264 BP509264 Hydra magnipapillata cDNA librar...	BP509264.1	1,00E-124	92
CL12CONTIG1	GB|BP508959.1|BP508959 BP508959 Hydra magnipapillata cDNA librar...	BP508959.1	5,00E-89	92
CL17CONTIG1	GB|CB888612.1|CB888612 taa79a10.x1 Hydra EST -III Hydra magnipap...	CB888612.1	1,00E-169	92
CL24CONTIG1	GB|CN774768.1|CN774768 tae73g05.y1 Hydra EST Darmstadt I Hydra m...	CN774768.1	3,00E-94	92
CL29CONTIG1	GB|CV565246.1|CV565246 taj73f04.y1 Hydra EST UCI 6 Hydra magnipa...	CV565246.1	3,00E-55	92
CL45CONTIG1	GB|CB073332.1|CB073332 taa31e01.y1 Hydra EST -II Hydra magnipapi...	CB073332.1	1,00E-115	92
CL53CONTIG1	GB|CN774922.1|CN774922 tae75e04.y1 Hydra EST Darmstadt I Hydra m...	CN774922.1	1,00E-160	92
CL56CONTIG1	GB|CN557797.1|CN557797 tae47d09.x1 Hydra EST Darmstadt I Hydra m...	CN557797.1	3,00E-63	92
CL66CONTIG1	GB|CA302647.1|CA302647 taa14f08.x1 Hydra cDNA library Hydra magn...	CA302647.1	6,00E-73	92
CL73CONTIG1	GB|BP506573.1|BP506573 BP506573 Hydra magnipapillata cDNA librar...	BP506573.1	4,00E-77	92
CL100CONTIG1	GB|CB888639.1|CB888639 taa79e04.x1 Hydra EST -III Hydra magnipap...	CB888639.1	1,00E-139	92
CL107CONTIG1	GB|CV151480.1|CV151480 tai69h06.x2 Hydra EST UCI 5 ALP Hydra mag...	CV151480.1	1,00E-111	92
CL114CONTIG1	GB|CV863817.2|CV863817 ACAB-aaa12f07.g1 Hydra UCI6- barcoded EST...	CV863817.2	1,00E-159	92
CL122CONTIG1	GB|BP520757.1|BP520757 BP520757 Hydra magnipapillata cDNA librar...	BP520757.1	4,00E-77	92
CL128CONTIG1	GB|CB271252.1|CB271252 taa17b12.x2 Hydra EST -II Hydra magnipapi...	CB271252.1	1,00E-100	92
CL141CONTIG1	GB|CN770372.1|CN770372 tae69c08.x1 Hydra EST Darmstadt I Hydra m...	CN770372.1	1,00E-108	92
CL146CONTIG1	GB|CD268504.1|CD268504 taa93g10.x1 Hydra EST -III Hydra magnipap...	CD268504.1	2,00E-34	92
CL157CONTIG1	GB|CA303015.1|CA303015 taa02a04.y1 Hydra cDNA library Hydra magn...	CA303015.1	1,00E-136	92
CL163CONTIG1	GB|CD681237.1|CD681237 tac23f06.y1 Hydra EST -IV Hydra magnipapi...	CD681237.1	1,00E-142	92
CL165CONTIG1	GB|CV465165.1|CV465165 taj23b07.x1 Hydra EST UCI 5 ALP Hydra mag...	CV465165.1	0	92
CL184CONTIG1	GB|BP515497.1|BP515497 BP515497 Hydra magnipapillata cDNA librar...	BP515497.1	1,00E-79	92
CL199CONTIG1	GB|CN632535.1|CN632535 taf02h06.x1 Hydra EST Darmstadt I Hydra m...	CN632535.1	3,00E-62	92
CL202CONTIG1	GB|CD266172.1|CD266172 tab14d01.x1 Hydra EST -III Hydra magnipap...	CD266172.1	5,00E-65	92
CL209CONTIG1	GB|CA302543.1|CA302543 taa16e04.y1 Hydra cDNA library Hydra magn...	CA302543.1	1,00E-148	92
CL220CONTIG1	GB|CN627638.1|CN627638 tae86g02.x1 Hydra EST Darmstadt I Hydra m...	CN627638.1	1,00E-101	92
CL224CONTIG1	GB|CD567612.1|CD567612 tab79g12.x1 Hydra EST -III Hydra magnipap...	CD567612.1	1,00E-141	92
CL226CONTIG1	GB|CB890627.1|CB890627 taa75f09.x1 Hydra EST -III Hydra magnipap...	CB890627.1	1,00E-120	92
CL235CONTIG1	GB|CF654425.1|CF654425 tac81b07.y1 Hydra EST -IV Hydra magnipapi...	CF654425.1	1,00E-111	92
CV284315.1	GB|BP506412.1|BP506412 BP506412 Hydra magnipapillata cDNA librar...	BP506412.1	1,00E-103	92
CV284320.1	GB|CO373915.1|CO373915 tah20a02.x2 Hydra EST -Kiel 5 Hydra magni...	CO373915.1	3,00E-93	92
CV284381.1	GB|DN813514.2|DN813514 ACAC-aac50k24.g1 Hydra EST UCI 7 Hydra ma...	DN813514.2	3,00E-89	92
CV284417.1	GB|CA302044.1|CA302044 taa09d10.y1 Hydra cDNA library Hydra magn...	CA302044.1	7,00E-88	92
CV284633.1	GB|CB072984.1|CB072984 taa26e11.y1 Hydra EST -II Hydra magnipapi...	CB072984.1	8,00E-21	92
CV285067.1	GB|CO372030.1|CO372030 tah15h11.x2 Hydra EST -Kiel 5 Hydra magni...	CO372030.1	2,00E-17	92
CV285131.1	GB|DT610033.1|DT610033 ACAG-aaa58h06.g1 Hydra_EST_UCI-9 Hydra ma...	DT610033.1	1,00E-113	92
CV285177.1	GB|CV182751.1|CV182751 tai87h08.y1 Hydra EST UCI 5 ALP Hydra mag...	CV182751.1	1,00E-154	92
CV285244.1	GB|CB073850.1|CB073850 taa21b11.y1 Hydra EST -II Hydra magnipapi...	CB073850.1	2,00E-55	92
CV285254.1	GB|CD266940.1|CD266940 tab06b05.x1 Hydra EST -III Hydra magnipap...	CD266940.1	1,00E-57	92
CV285257.1	GB|BP505798.1|BP505798 BP505798 Hydra magnipapillata cDNA librar...	BP505798.1	1,00E-135	92
CV285466.1	GB|CV464828.1|CV464828 taj26e10.y1 Hydra EST UCI 5 ALP Hydra mag...	CV464828.1	0	92
CV285628.1	GB|DN603328.2|DN603328 ACAC-aac09g21.g1 Hydra EST UCI 7 Hydra ma...	DN603328.2	0	92
CV285637.1	GB|CA302590.1|CA302590 taa13c10.x1 Hydra cDNA library Hydra magn...	CA302590.1	1,00E-22	92
CV285707.1	GB|DT616439.1|DT616439 ACAH-aaa14f12.g1 Hydra_EST_UCI-10 Hydra m...	DT616439.1	1,00E-165	92
CL32CONTIG1	GB|CO375019.1|CO375019 tah26e08.x1 Hydra EST -Kiel 5 Hydra magni...	CO375019.1	1,00E-136	93
CL39CONTIG1	GB|CV042287.1|CV042287 tai54b04.y2 Hydra EST UCI 5 ALP Hydra mag...	CV042287.1	0	93
CL40CONTIG1	GB|BP508232.1|BP508232 BP508232 Hydra magnipapillata cDNA librar...	BP508232.1	4,00E-64	93
CL60CONTIG1	GB|CO376584.1|CO376584 tah42b03.x1 Hydra EST -Kiel 5 Hydra magni...	CO376584.1	1,00E-101	93
CL69CONTIG1	GB|CF674708.1|CF674708 tac86a05.x1 Hydra EST -IV Hydra magnipapi...	CF674708.1	1,00E-158	93
CL76CONTIG1	GB|CA303219.1|CA303219 taa04g05.y1 Hydra cDNA library Hydra magn...	CA303219.1	2,00E-89	93
CL85CONTIG1	GB|DT615928.1|DT615928 ACAH-aab12c07.g1 Hydra_EST_UCI-10 Hydra m...	DT615928.1	4,00E-86	93
CL88CONTIG1	GB|CD606798.1|CD606798 tac20a04.x1 Hydra EST -IV Hydra magnipapi...	CD606798.1	1,00E-141	93
CL92CONTIG1	GB|CB073343.1|CB073343 taa31f03.y1 Hydra EST -II Hydra magnipapi...	CB073343.1	1,00E-159	93
CL115CONTIG1	GB|CA302093.1|CA302093 taa10b03.y1 Hydra cDNA library Hydra magn...	CA302093.1	2,00E-94	93
CL148CONTIG1	GB|CA302115.1|CA302115 taa10d09.y1 Hydra cDNA library Hydra magn...	CA302115.1	8,00E-49	93
CL160CONTIG1	GB|CN558007.1|CN558007 tae46f06.y1 Hydra EST Darmstadt I Hydra m...	CN558007.1	1,00E-170	93
CL177CONTIG1	GB|BP518542.1|BP518542 BP518542 Hydra magnipapillata cDNA librar...	BP518542.1	1,00E-138	93
CL194CONTIG1	GB|CB073201.1|CB073201 taa29g07.y1 Hydra EST -II Hydra magnipapi...	CB073201.1	1,00E-142	93
CL208CONTIG1	GB|CD267611.1|CD267611 tab91e04.x1 Hydra EST -III Hydra magnipap...	CD267611.1	1,00E-68	93
CL216CONTIG1	GB|CO537490.1|CO537490 tah77h12.x1 Hydra EST UCI 5 Hydra magnipa...	CO537490.1	1,00E-103	93
CL230CONTIG1	GB|CD268917.1|CD268917 tab23a05.x1 Hydra EST -III Hydra magnipap...	CD268917.1	3,00E-64	93
CL236CONTIG1	GB|DT612109.1|DT612109 ACAG-aaa73g02.g1 Hydra_EST_UCI-9 Hydra ma...	DT612109.1	1,00E-114	93
CV284404.1	GB|CB073120.1|CB073120 taa28e10.y1 Hydra EST -II Hydra magnipapi...	CB073120.1	2,00E-69	93
CV285082.1	GB|CA302579.1|CA302579 taa13b05.x1 Hydra cDNA library Hydra magn...	CA302579.1	5,00E-95	93
CV285089.1	GB|CA301952.1|CA301952 taa11e06.x1 Hydra cDNA library Hydra magn...	CA301952.1	4,00E-92	93
CV285117.1	GB|CD268910.1|CD268910 tab22h08.x1 Hydra EST -III Hydra magnipap...	CD268910.1	7,00E-66	93
CV285119.1	GB|CO377730.1|CO377730 tah58c03.x1 Hydra EST -Kiel 5 Hydra magni...	CO377730.1	5,00E-64	93
CV285193.1	GB|CD606832.1|CD606832 tac20f09.x1 Hydra EST -IV Hydra magnipapi...	CD606832.1	3,00E-11	93
CV285378.1	GB|CA301778.1|CA301778 taa07h11.y1 Hydra cDNA library Hydra magn...	CA301778.1	6,00E-66	93
CV285621.1	GB|CN554588.1|CN554588 tae30h02.x1 Hydra EST Darmstadt I Hydra m...	CN554588.1	1,00E-124	93
CL7CONTIG1	GB|CF653933.1|CF653933 tac78d03.y1 Hydra EST -IV Hydra magnipapi...	CF653933.1	1,00E-120	94
CL10CONTIG1	GB|BP505283.1|BP505283 BP505283 Hydra magnipapillata cDNA librar...	BP505283.1	3,00E-99	94
CL41CONTIG1	GB|CB889752.1|CB889752 taa40g10.x3 Hydra EST -III Hydra magnipap...	CB889752.1	5,00E-83	94

CL50CONTIG1	GB|CV463937.1|CV463937 taj33c02.y1 Hydra EST UCI 5 ALP Hydra mag...	CV463937.1	2,00E-45	94
CL77CONTIG1	GB|CB073969.1|CB073969 taa22h03.y1 Hydra EST -II Hydra magnipapi...	CB073969.1	5,00E-92	94
CL79CONTIG1	GB|CD268953.1|CD268953 tab23g03.x1 Hydra EST -III Hydra magnipap...	CD268953.1	9,00E-15	94
CL80CONTIG1	GB|CA303163.1|CA303163 taa04a06.y1 Hydra cDNA library Hydra magn...	CA303163.1	2,00E-75	94
CL175CONTIG1	GB|CA302050.1|CA302050 taa09e04.y1 Hydra cDNA library Hydra magn...	CA302050.1	1,00E-98	94
CL212CONTIG1	GB|CV182623.1|CV182623 tai86b12.y1 Hydra EST UCI 5 ALP Hydra mag...	CV182623.1	1,00E-89	94
CL221CONTIG1	GB|CD267642.1|CD267642 tab92b05.x1 Hydra EST -III Hydra magnipap...	CD267642.1	1,00E-139	94
CL228CONTIG1	GB|CA302590.1|CA302590 taa13c10.x1 Hydra cDNA library Hydra magn...	CA302590.1	3,00E-90	94
CL241CONTIG1	GB|CA303216.1|CA303216 taa04g01.y1 Hydra cDNA library Hydra magn...	CA303216.1	4,00E-14	94
CV284496.1	GB|CA301765.1|CA301765 taa07g09.y1 Hydra cDNA library Hydra magn...	CA301765.1	1,00E-117	94
CV285064.1	GB|CB888259.1|CB888259 taa89f11.x1 Hydra EST -III Hydra magnipap...	CB888259.1	3,00E-74	94
CV285256.1	GB|CD567717.1|CD567717 tab81c08.x1 Hydra EST -III Hydra magnipap...	CD567717.1	1,00E-102	94
CV285435.1	GB|CV042103.1|CV042103 tai66b12.x1 Hydra EST UCI 5 ALP Hydra mag...	CV042103.1	1,00E-109	94
CV285464.1	GB|CB888099.1|CB888099 taa87c10.x1 Hydra EST -III Hydra magnipap...	CB888099.1	3,00E-27	94
CV285597.1	GB|DT605264.1|DT605264 ACAH-aaa06c05.g1 Hydra_EST_UCI-10 Hydra m...	DT605264.1	1,00E-151	94
CL23CONTIG1	GB|CA301963.1|CA301963 taa11g09.x1 Hydra cDNA library Hydra magn...	CA301963.1	2,00E-39	95
CL46CONTIG1	GB|CO376856.1|CO376856 tah44a10.x1 Hydra EST -Kiel 5 Hydra magni...	CO376856.1	2,00E-65	95
CL132CONTIG1	GB|BP504792.1|BP504792 BP504792 Hydra magnipapillata cDNA librar...	BP504792.1	5,00E-21	95
CL158CONTIG1	GB|BP504674.1|BP504674 BP504674 Hydra magnipapillata cDNA librar...	BP504674.1	1,00E-70	95
CV284393.1	GB|BP505024.1|BP505024 BP505024 Hydra magnipapillata cDNA librar...	BP505024.1	5,00E-71	95
CV285157.1	GB|CB072632.1|CB072632 taa25a04.x1 Hydra EST -II Hydra magnipapi...	CB072632.1	1,00E-101	95
CV285370.1	GB|CB888893.1|CB888893 taa43c04.x3 Hydra EST -III Hydra magnipap...	CB888893.1	2,00E-79	95
CL14CONTIG1	GB|CB888746.1|CB888746 taa41a05.x3 Hydra EST -III Hydra magnipap...	CB888746.1	0	96
CL49CONTIG1	GB|CF656403.1|CF656403 tac54a06.y1 Hydra EST -IV Hydra magnipapi...	CF656403.1	2,00E-24	96
CL87CONTIG1	GB|CN626309.1|CN626309 tae97c03.x1 Hydra EST Darmstadt I Hydra m...	CN626309.1	1,00E-175	96
CL198CONTIG1	GB|CA301982.1|CA301982 taa12c04.x1 Hydra cDNA library Hydra magn...	CA301982.1	1,00E-117	96
CL206CONTIG1	GB|CN776141.1|CN776141 tae77h01.y1 Hydra EST Darmstadt I Hydra m...	CN776141.1	1,00E-123	96
CL222CONTIG1	GB|DT614996.1|DT614996 ACAH-aaa13d05.g1 Hydra_EST_UCI-10 Hydra m...	DT614996.1	1,00E-114	96
CL227CONTIG1	GB|CF657083.1|CF657083 tac68f10.y1 Hydra EST -IV Hydra magnipapi...	CF657083.1	7,00E-91	96
CV284352.1	GB|CN553162.1|CN553162 tae25c08.x1 Hydra EST Darmstadt I Hydra m...	CN553162.1	1,00E-40	96
CV285152.1	GB|CB073174.1|CB073174 taa29d06.y1 Hydra EST -II Hydra magnipapi...	CB073174.1	3,00E-71	96
CV285420.1	GB|CN775969.1|CN775969 tae79g05.x1 Hydra EST Darmstadt I Hydra m...	CN775969.1	3,00E-94	96
CV285525.1	GB|CA301771.1|CA301771 taa07h04.y1 Hydra cDNA library Hydra magn...	CA301771.1	2,00E-62	96
CL94CONTIG1	GB|CA303335.1|CA303335 taa07e07.x1 Hydra cDNA library Hydra magn...	CA303335.1	4,00E-60	98
CL120CONTIG1	GB|DN248282.2|DN248282 ACAE-aaa25b10.b1 Hydra EST UCI 5 Hydra ma...	DN248282.2	3,00E-19	98
CV285385.1	GB|CV887927.1|CV887927 tak92c10.y1 Hydra EST UCI 6 Hydra magnipa...	CV887927.1	8,00E-93	98
CV285492.1	GB|CA303158.1|CA303158 taa03h11.y1 Hydra cDNA library Hydra magn...	CA303158.1	3,00E-21	98
CV285521.1	GB|CV465102.1|CV465102 taj21g05.x1 Hydra EST UCI 5 ALP Hydra mag...	CV465102.1	1,00E-160	98
CL3CONTIG2	GB|CB889334.1|CB889334 taa34f06.x3 Hydra EST -III Hydra magnipap...	CB889334.1	0	99
CL3CONTIG3	GB|CF599902.1|CF599902 tac29f09.x1 Hydra EST -IV Hydra magnipapi...	CF599902.1	1,00E-179	99
CL11CONTIG1	GB|CD565913.1|CD565913 tab87g12.x1 Hydra EST -III Hydra magnipap...	CD565913.1	0	99
CV285182.1	GB|CA301678.1|CA301678 taa06e12.y1 Hydra cDNA library Hydra magn...	CA301678.1	2,00E-58	99
CL3CONTIG1	GB|CF655470.1|CF655470 tac89h03.y1 Hydra EST -IV Hydra magnipapi...	CF655470.1	1,00E-125	100
CL155CONTIG1	GB|CA301882.1|CA301882 taa09e10.x1 Hydra cDNA library Hydra magn...	CA301882.1	5,00E-14	100
