# Supplementary material for: A Novel Gene Family Controls Species-Specific Morphological Traits in Hydra
Source: PLoS Biol. 2008 Nov 18;6(11):e278. doi: 10.1371/journal.pbio.0060278 (PMC2586386; doi:10.1371/journal.pbio.0060278)
Supplement: Table S4 — Mean number of tentacles per polyp at 42, 66, and 130 h after decapitation (mean ± standard deviation) in independent experiments (I, II, III, etc.). (15 KB PDF) [file pbio.0060278.st004.pdf]

Table S4:

Tentacle regeneration experiments. Mean number of tentacles per polyp at 42, 66 and 130 hours after decapitation (mean±s.d.) in independent experiments (I, II, III, …).

(1) Tentacle regeneration in transgenic *Hydra vulgaris* AEP *A14 vs. Hydra vulgaris* AEP wild-type (**Fig. 5 c**). Total number of animals: AEP^{A14}, n=156; AEP^{WT}, n=124.

	42 hours		66 hours		130 hours	
	AEP^{A14}	AEP^{WT}	AEP^{A14}	AEP^{WT}	AEP^{A14}	AEP^{WT}
I)	2,36±1,67	1±1,25	3,64±1,69	2,46±1,2	6,08±1,47	5,81±0,94
II)	5,44±1,58	2,23±1,6	7,39±1,55	4,82±1,71	8,61±1,39	6,38±1,07
III)	4,8±1,27	1,9±0,86	5,77±1,33	3,77±1,63	6,78±1,05	5,97±1,12
mean±s.d.	4,20±1,5	1,71±1,24	5,60±1,52	3,68±1,51	7,16±1,3	6,05±1,04

(2) Tentacle regeneration in transgenic *Hydra vulgaris* AEP *A14 vs. Hydra vulgaris* AEP Ecto-1 transgenic line (**Fig. 5 c**). Total number of animals: AEP^{A14}, n=106; Ecto-1, n=102

	42 hours		66 hours		130 hours	
	AEP^{A14}	Ecto-1	AEP^{A14}	Ecto-1	AEP^{A14}	Ecto-1
I)	3,8±1.0	1,2±1,17	5,9±0,85	2,3±1,65	6,9±0,93	5,7±0,95
II)	3,0±0,95	1,5±1,68	5,5±1,06	2,9±1,73	7,9±1,55	5,0±0,49
III)	3,4±1,86	1,4±1,58	5,5±1,48	3,1±1,68	7,1±1,66	5,9±1,42
IV)	4,2±1,77	2,2±1,79	6,1±1,78	3,4±1,43	7,1±2,03	5,9±1,62
mean±s.d.	3,6±1,61	1,6±1,64	5,8±1,44	3,0±1,64	7,2±1,70	5,7±1,31

(3) Tentacle regeneration in *H.oligactis* polyps electroporated with *oHYM301A* dsRNA vs. polyps electroporated with *GFP* dsRNA (**Fig. 7 e**). Total number of animals: *oHYM301A* dsRNA, n=49; *GFP* dsRNA, n=55.

	42 hours		66 hours		130 hours	
	oHYM301A	***GFP***	***oHYM301A***	***GFP***	***oHYM301A***	***GFP***
I)	2,5±1,51	3,9±1,06	3,0±1,58	4,3±1.0	3,2±1,57	4,3±1.0
II)	2,4±1,85	4,0±1,3	3,1±1,92	4,3±1,03	4,0±1,37	4,5±1,23
III)	2,7±1,91	3,8±1,21	3,6±1,79	5,1±1,22	4,2±1,52	5,2±1,21
mean±s.d.	2,6±1,76	3,9±1,18	3,2±1,77	4,5±1,14	3,9±1,51	4,6±1,21
