# Supplementary material for: A Novel Gene Family Controls Species-Specific Morphological Traits in Hydra
Source: PLoS Biol. 2008 Nov 18;6(11):e278. doi: 10.1371/journal.pbio.0060278 (PMC2586386; doi:10.1371/journal.pbio.0060278)
Supplement: Table S5 — (7 KB PDF) [file pbio.0060278.st005.pdf]

Table S5: Primers used in this study

Primer name	Sequence (5` to 3`)
GAPDH_F	GCC TTA TGA CAA CCA TTC AT
GAPDH_R	TCA ACA ACA GAA ACA TCT GC
ACTIN35	CCA AAA TAG ATC CTC CGA TCC
ACTIN34	AAG CTC TTC CCT CGA GAA ATC
Hym301_F(2)Pst	GTC ACT GCA GCC CCT AAA ATG ACA CGT GTT TGC G
Hym301_R(295)Pst	GGG GCTGCA GCA TTT TTT TGC GTA ATT TCT TTT TTA CC
oHYM301A_F(40)	GAA TTT GTA TAT TAT TAC TTT GTG TAG
oHYM301A_R(144)	CAC TTT GAA CGA ATG AAG GGT C
AEP301A_F	GAC TGC TCT ATC GAG AGA TTC
AEP301A_R	TAG ATG GAG GAA TTT CAA GAG AG
gmHYMA_E1F	CAC ATA CCC TAA AAT GAC ACG TG
gmHYMA_E3R	CCA TTT TTT AAA CTA TTG ATC ACA TGT TC
Alx_F(422)	CCC CTA GTA ACT CAA TAT GTA G
Alx_R(631)	GTA CAA GCA AGA TCT TGC AGT G
oHYM301_F(32)	CTT TGC ATT GAT TGA TGC GCA ACC
o301Ai_T7_F	CAT TAA TAC GAC TCA CTA TAG GGG ATC GTA AAA CAA TAT TTA CTT C
o301Ai_T7_R	TAC TAA TAC GAC TCA CTA TAG GGA CAT TGT TTA AAC ATA TTA TCA TC
T7_GFP_F	TAA TAC GAC TCA CTA TAG GGA CAG TGG AGA GGG TGA AGG TGA TGC
T7_GFP_R	TAA TAC GAC TCA CTA TAG GGA GGC AGA TTG TGT GGA CAG GTA ATG G
